# New Prenylated Isoindolinone Alkaloids and Polyketides from the Mangrove-Derived Fungus *Aspergillus* sp. SCSIO 41440

**DOI:** 10.3390/md24090297

**Published:** 2026-08-25

**Authors:** Chun Yang, Yi Chen, Zhifei Xiong, Jian Cai, Chunmei Chen, Bingling Liu, Yonghong Liu, Xuefeng Zhou, Huaming Tao

**Affiliations:** 1 School of Traditional Chinese Medicine, Southern Medical University, Guangzhou 510515, China; 3201008013@smu.edu.cn (C.Y.); xiongzhifei@i.smu.edu.cn (Z.X.); qazwsx256930@163.com (B.L.); 2 State Key Laboratory of Tropical Oceanography/Guangdong Key Laboratory of Marine Materia Medica, South China Sea Institute of Oceanology, Chinese Academy of Sciences, Guangzhou 510301, China; chenyi221@mails.ucas.ac.cn (Y.C.); caijian@scsio.ac.cn (J.C.); yonghongliu@scsio.ac.cn (Y.L.); xfzhou@scsio.ac.cn (X.Z.); 3 University of Chinese Academy of Sciences, Beijing 100049, China; 4 School of Chemistry, South China Normal University, Guangzhou 510006, China; chencm@m.scnu.edu.cn

**Keywords:** mangrove-derived fungus, *Aspergillus* sp., alkaloids, polyketides, acetylcholinesterase

## Abstract

New prenylated isoindolinone alkaloids, aspernidines C-E (**1**–**3**), a new diketopiperazine alkaloid, protubonine C (**4**), and new polyketides, (±)-asperisocoumarin J and asperisocoumarin K (**5** and **6**), were isolated from the mangrove-derived fungus *Aspergillus* sp. SCSIO 41440, together with six known compounds (**7**–**12**). Comprehensive NMR and mass spectrometric analysis, single-crystal X-ray diffraction, and calculated electronic circular dichroism (ECD) were employed for the structural elucidation and stereochemical assignment of these compounds. All compounds were evaluated for their acetylcholinesterase (AChE) inhibitory activity. Compound **10** exhibited potent AChE inhibition with an IC_50_ value of 3.16 ± 1.16 μM. Molecular docking analysis further revealed that **10** binds strongly to the AChE active site (PDB: 4EY7). These findings suggest that compound **10** represents a candidate scaffold for further investigation as an AChE inhibitor.

## 1. Introduction

Mangroves, defined as plant communities inhabiting the intertidal zones of tropical and subtropical coastlines, constitute one of the most productive and biologically diverse ecosystems globally, providing a unique habitat for endophytic fungi. The extreme and dynamic environmental conditions prevalent in mangrove habitats impose unique selective pressures on endophytic fungi, driving the evolution of specialized metabolic pathways. Consequently, secondary metabolites produced by mangrove-derived fungi often exhibit novel scaffolds and pronounced pharmacological activities, positioning them as invaluable resources for natural product-based drug discovery [1,2,3].

*Aspergillus* species are widely distributed in habitats, including plants and soil sediments, and their species diversity is second only to that of *Penicillium* species [4]. Among studies on novel natural products from marine sources, *Aspergillus* species are the most extensively investigated fungi [5]. They play an important role in natural product discovery, as do *Penicillium* fungi. The secondary metabolites produced by these fungi are structurally diverse and biologically significant, encompassing multiple chemical classes, including polyketides, alkaloids, peptides, and anthraquinones [6]. Studies have shown that these metabolites exhibit significant AChE inhibitory activity, suggesting their potential for the treatment of neurodegenerative diseases such as Alzheimer’s disease [7]. In addition, broad-spectrum antibacterial activities of these metabolites have attracted widespread attention, and they might be of great significance for the prevention and control of clinically drug-resistant strains [6].

**Figure 1:**
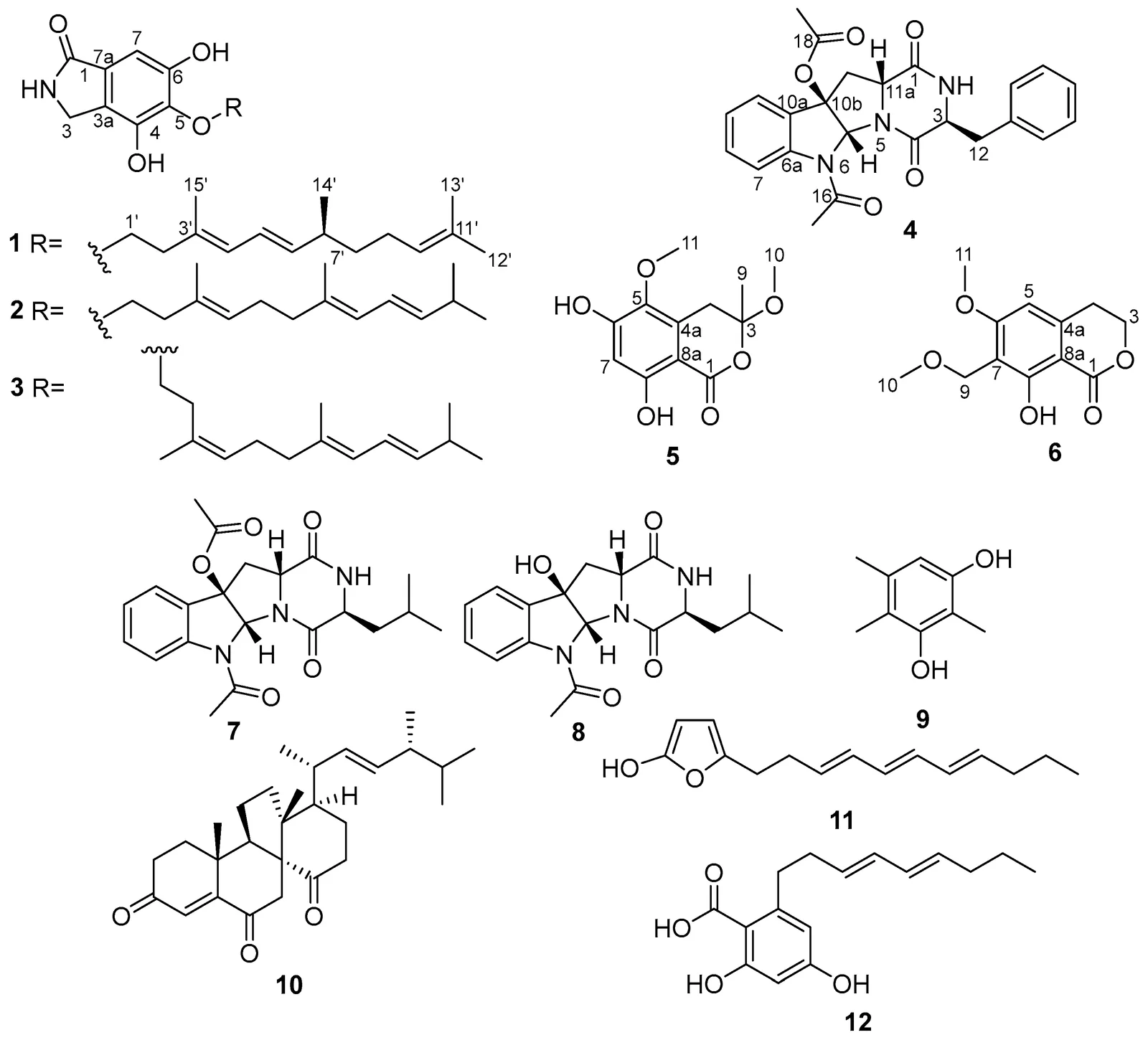
Chemical structures of compounds **1**–**12**.

In an ongoing effort to discover bioactive metabolites from marine-derived fungi, the *Aspergillus* sp. strain SCSIO 41440, isolated from mangrove sediments in Sri Lanka, was systematically investigated. As a result, six new compounds were isolated and identified, together with six known compounds (Figure 1). This paper reports the isolation and structural elucidation of new compounds **1**–**6**, along with the biological evaluation, including enzyme inhibitory and antimicrobial properties.

## 2. Results and Discussion

### 2.1. Structural Determination

**Figure 2:**
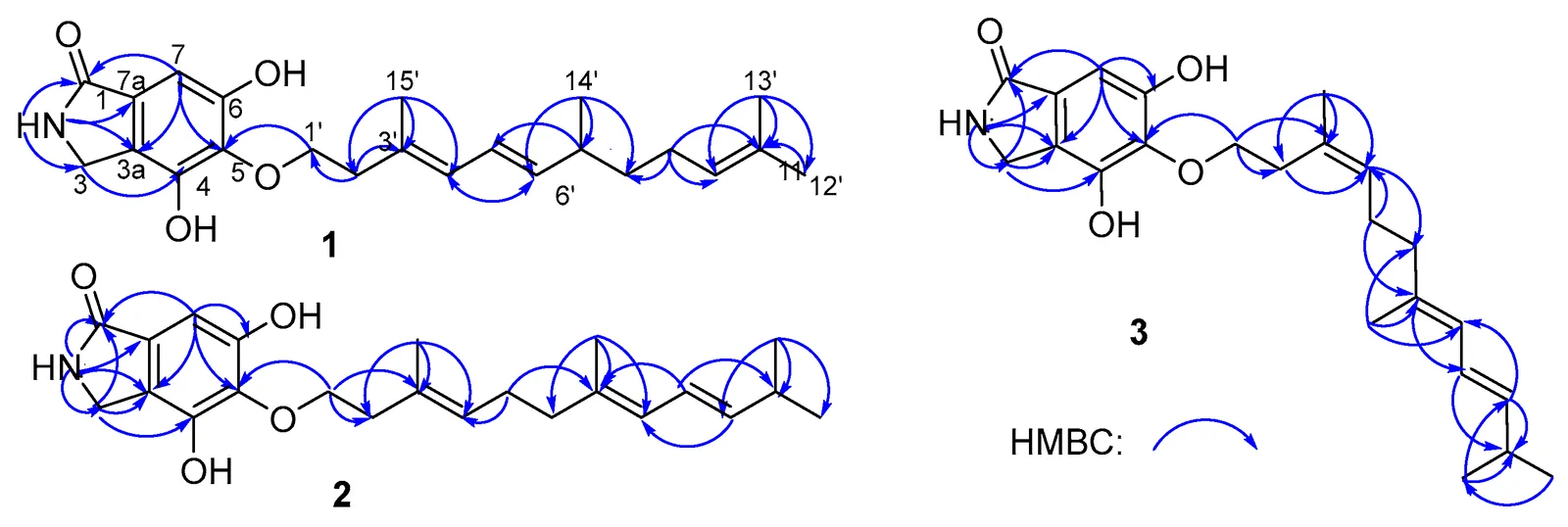
Key HMBC correlations of compounds **1**–**3**.

**Figure 3:**
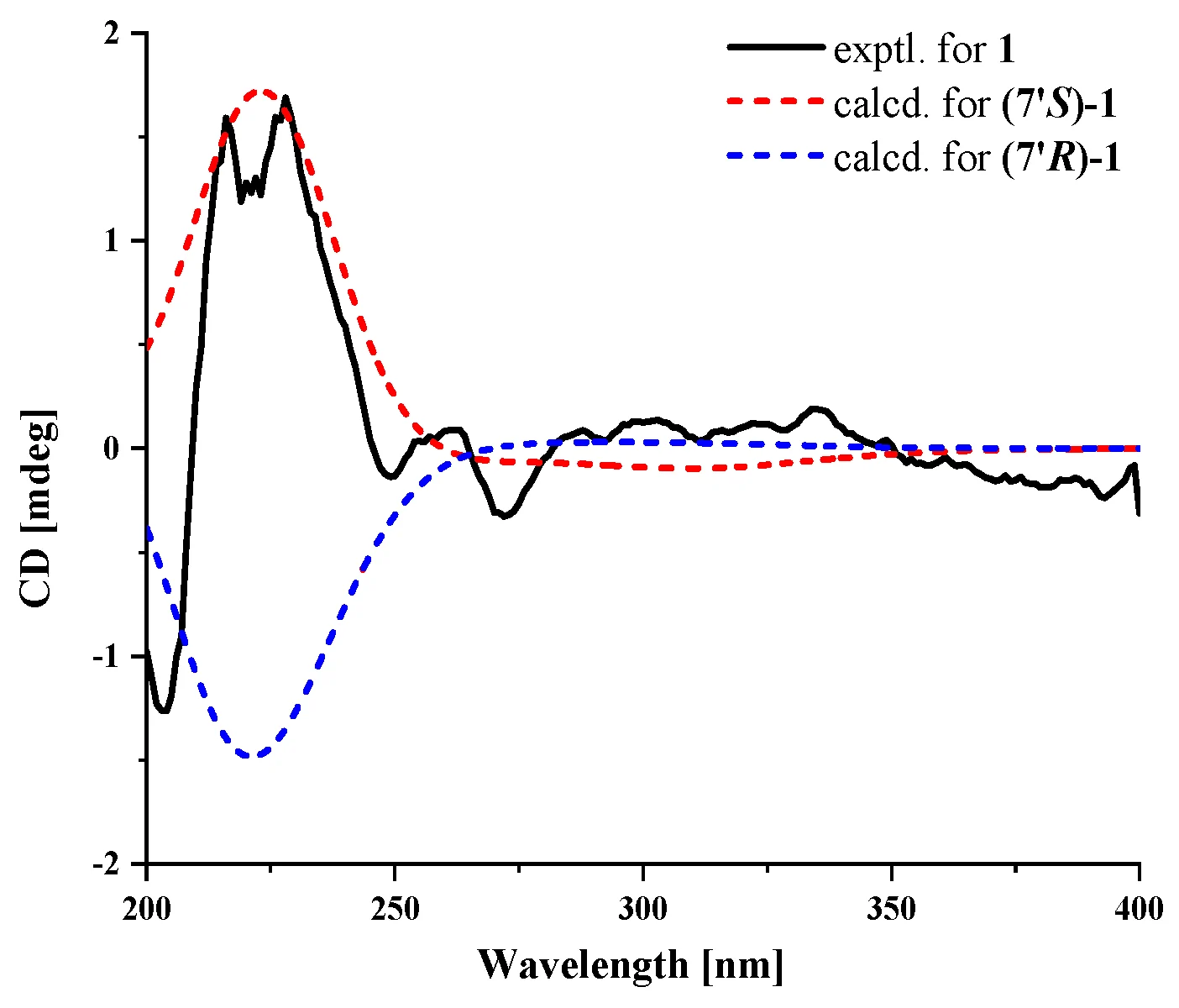
Experimental and calculated ECD spectra of **1**.

Aspernidine C (**1**) was obtained as a brown oil. Its molecular formula was determined as C_23_H_31_NO_4_ by HRESIMS, which showed a protonated molecular ion peak at *m*/*z* 386.2325 [M + H]^+^ (calcd. for C_23_H_32_NO_4_, 386.2326), corresponding to nine degrees of unsaturation. The ^1^H NMR and HSQC spectra (Table 1) indicated that there were twenty-four sp^3^-hybridized protons and five sp^2^-hybridized protons. The ^13^C NMR data showed twenty-three carbon atoms, including one carbonyl carbon, seven olefinic quaternary carbons, six methine carbons (five of which are olefinic), five methylene carbons, and four methyl carbons. The nine degrees of unsaturation were accounted for by one carbonyl group, six olefinic bonds (12 sp^2^ carbon signals) and two rings. The NMR data of compound **1** were similar to those of aspernidine A, a prenylated isoindolinone alkaloid from *Aspergillus nidulans* [8]. The HMBC correlations (Figure 2) of H-7 to C-5, C-3a, and C-6, together with those of 2-NH to C-3a, C-7a, C-3, and C-1, confirmed the presence of the core isoindolinone skeleton. The remaining signals were consistent with an aliphatic chain containing three double bonds, including a terminal double bond at the 10′/11′ position bearing two methyl substituents. The main difference between **1** and aspernidine A lies in the positions of the double bonds within the prenyl side chain. The coupling patterns of the olefinic protons H-4′, H-5′, and H-6′ in the ^1^H NMR spectrum, combined with the HMBC correlations of H-4′ to C-6′ and of H-5′ to C-3′, indicated the presence of a trans-conjugated diene unit at the 3′/4′ and 5′/6′ positions, with both double bonds being of *E* configuration. In addition, the HMBC correlation of H-15′ to the olefinic quaternary carbon C-3′ indicated that this methyl group is attached to the structural unit. The above data established the planar structure elucidation of **1**. As **1** is a chiral molecule, ECD calculations (Figure 3) were performed for the two possible configurations, 7′*R*-**1** and 7′*S*-**1**. The results showed that the calculated ECD spectrum of 7′*S*-**1** matched the experimental curve, thus allowing the absolute configuration of 1 to be assigned as 7′*S*. Thus, compound **1** was unequivocally identified as aspernidine C.

Aspernidines D (**2**) and E (**3**) were found to be isomers of **1**. The differences between **2** and **1** were deduced from the ^1^H NMR chemical shifts (Table 1), coupling constants, and HMBC correlations (Figure 2), which indicated variations in the positions of the olefinic double bonds along the aliphatic chain. In compound **2**, a trans-conjugated diene unit was present at the 7′/8′ and 9′/10′ positions, while the 3′/4′ double bond was isolated; all double bonds were assigned to be of *E* configuration. This distinction was further supported by the HMBC correlation of H-14′ (*δ*_H_ 1.68) to the olefinic quaternary carbon (*δ*_C_ 135.6). Based on these structural features, compound **2** was identified as aspernidine D. Comparison of the 1D and 2D NMR data of **3** and **2** revealed differences only in chemical shift in the methyl signal at the 15′ position, suggesting that the double bond at 3′/4′ differed solely in its configuration, being *Z* in **3** and *E* in **2**. Accordingly, compound **3** was identified as aspernidine E.

**Figure 4:**
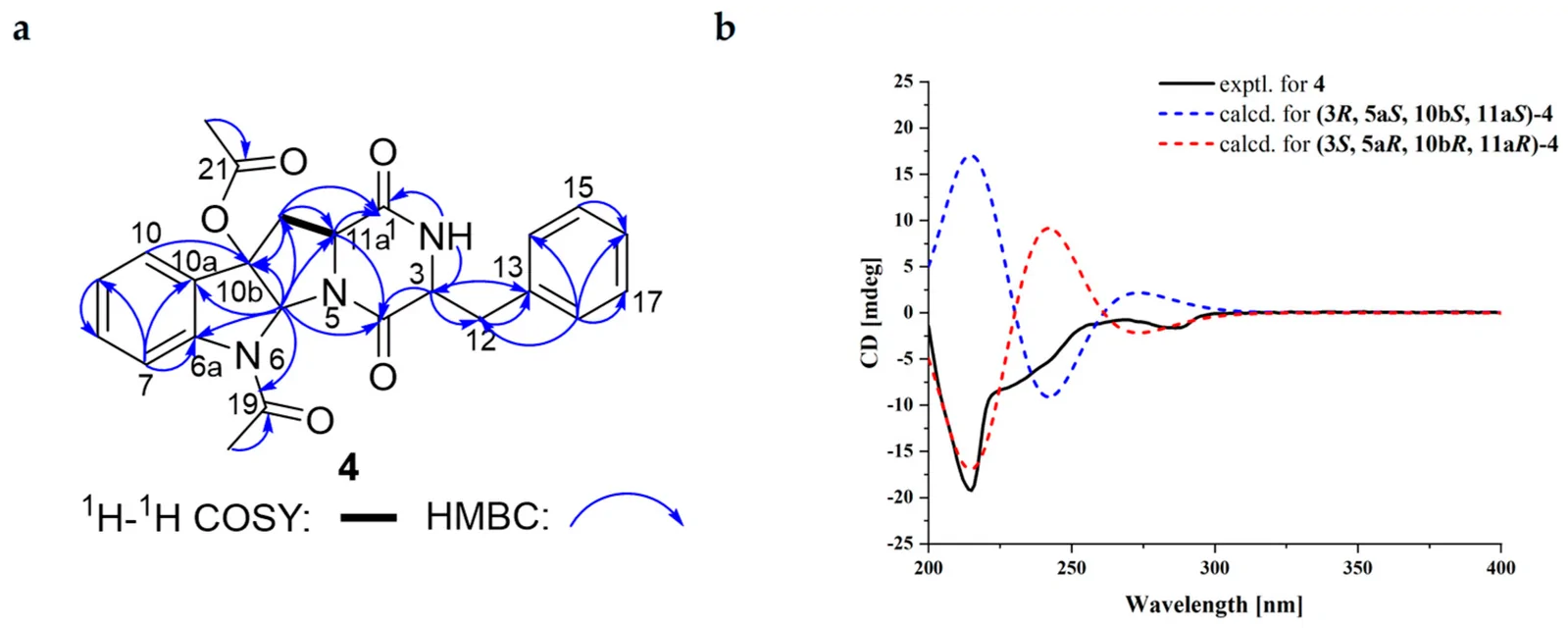
(**a**) Key HMBC and ^1^H-^1^H COSY correlations of **4**. (**b**) Experimental and calculated ECD spectra of **4**.

**Figure 5:**
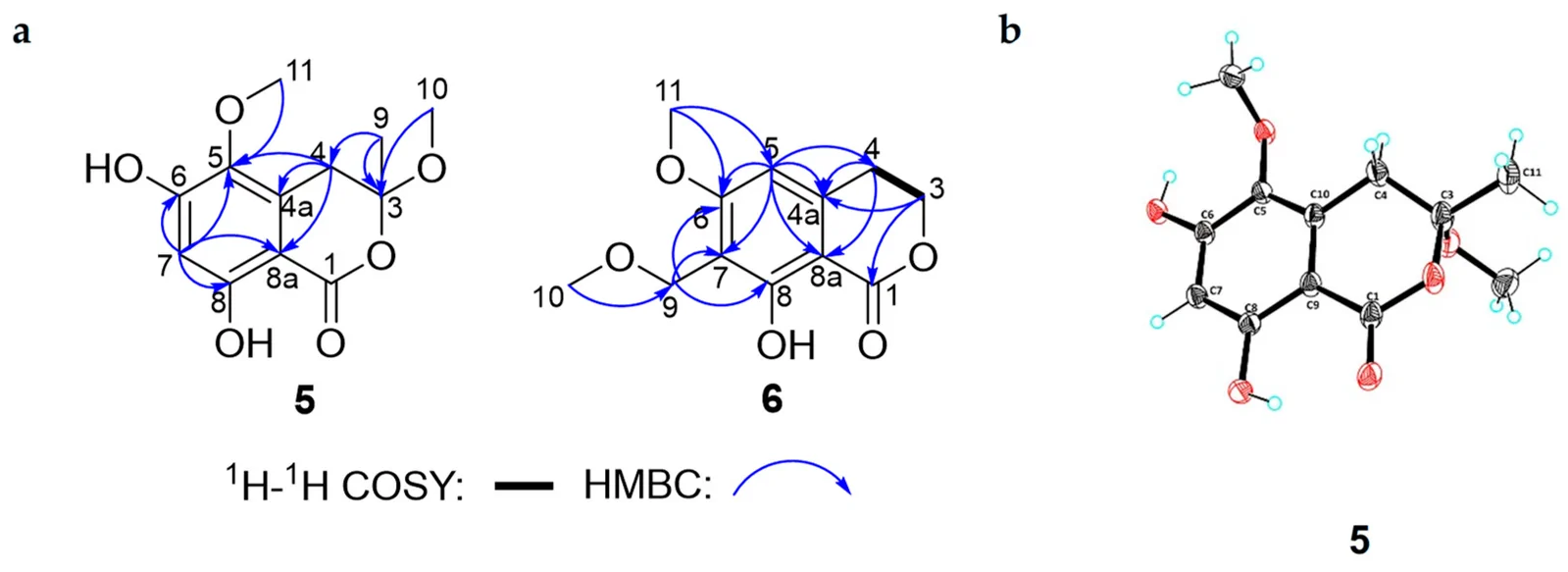
(**a**) Key HMBC and ^1^H-^1^H COSY correlations of **5** and **6**. (**b**) X-ray crystallographic structure of **5**.

Protubonine C (**4**) was isolated as a yellow solid. Its molecular formula was determined as C_24_H_23_N_3_O_5_, based on HRESIMS data (calcd. for C_24_H_23_N_3_NaO_5_, 456.1530) and NMR spectroscopic analysis, corresponding to fifteen degrees of unsaturation. The ^1^H NMR, ^13^C NMR, and HSQC spectra (Table 2) indicated the presence of twenty-four carbon atoms, consisting of four carbonyl carbons, three olefinic quaternary carbons, one oxygen-bearing quaternary carbon, twelve methine carbons (nine of which are olefinic), two methylene carbons, and two methyl carbons. Key HMBC correlations from the amide proton at *δ*_H_ 8.44 to the carbonyl carbons at *δ*_C_ 165.5 (C-1), and from H-11a to C-1 and C-4 (Figure 4a), confirmed the presence of a diketopiperazine ring. The existence of two aromatic rings, one of which is 1,2-disubstituted, was established by interpretation of the aromatic region coupling patterns in the ^1^H NMR spectrum. In addition, the HMBC correlations of H-5a to C-6a, C-10a and C-10b, and of H-10 to C-10b, suggested the presence of an indoline ring unit. The connections between the two structural fragments were determined with HMBC correlations of H-11 with C-1, C-11a and C-10b, and of H-5a with C-4 and C-11a, i.e., there existed a carbon–nitrogen bond between C-5a and N-5, and a linkage through the –CH_2_– unit connecting C-11a and C-10b. The presence of two acetyl groups was confirmed by HMBC correlations of H_3_-20 to C-19 and of H_3_-22 to C-21 and C-10b; furthermore, the HMBC correlation of H-5a to C-19 established the attachment of this group to the nitrogen atom of the indoline moiety (N-6). The NOESY correlations of H-5a with H-11a revealed that these two protons are syn; however, the lack of correlation between H-3 and H-11a suggested that the two protons are placed on opposite faces. Consequently, two possible relative configurations were considered for **4** (3*R*, 5a*S*, 11a*S*) and (3*S*, 5a*R*, 11a*R*). Based on the comparison of the NMR spectrum with protubonine B (**7**) [9], **4** shared the same basic skeleton as compound **7**; meanwhile, their specific rotations have the same sign: compound **7** has ${\left[\alpha \right]}_{D}^{25}$-269 (c 0.55, CH_3_OH), while **4** has ${\left[\alpha \right]}_{D}^{25}$-159.5 (c 0.1, CH_3_OH). It was further inferred that the absolute configuration at C-10b in **4** is consistent with that in **7**, leading to two other possible assignments: (3*R*, 5a*S*, 10b*S*, 11a*S*)-**4** and (3*S*, 5a*R*, 10b*R*, 11a*R*)-**4**. Furthermore, ECD calculations (Figure 4b) were performed for both candidate configurations. The results showed that (3*S*, 5a*R*, 10b*R*, 11a*R*)-**4** matched the experimental curve. The absolute configuration of **4** was determined to be 3*S*, 5a*R*, 10b*R*, 11a*R*. Compound **4** was unequivocally characterized as protubonine C.

(±)-Asperisocoumarin J (**5**) was isolated as a pale red amorphous powder. The molecular formula of **5** was determined to be C_12_H_14_O_6_ by HRESIMS data at *m*/*z* 253.0719 [M − H]^−^ (calcd. for C_12_H_13_O_6_, 253.0718). The ^1^H NMR and HSQC spectra (Table 3) revealed the existence of eleven sp^3^-hybridized protons and one sp^2^-hybridized proton. The ^13^C NMR spectrum indicated that there were twelve carbon atoms, including one carbonyl carbon, six olefinic quaternary carbons (five of which are olefinic), one methine carbon, one methylene carbon, and three methyl carbons (two of which are methoxy carbons). Six degrees of unsaturation were accounted for by the presence of one carbonyl group, three olefinic bonds (i.e., six sp^2^ carbon signals), and two rings. The presence of a benzene unit was established by HMBC correlations (Figure 5a) of H-7 with C-5, C-8a, C-6, and C-8. A correlation of H-11 with C-5 established the attachment position of one methoxy group. Furthermore, correlations of 9-CH_3_ with C-3 and C-4 indicated that this methyl group was attached to C-3, adjacent to C-4, while the correlation of H-10 with C-3 confirmed the attachment position of another methoxy group. Additionally, the HMBC correlation of H-7 with the carbonyl carbon of C-1, together with the degree of unsaturation, supported the existence of the second ring in the structure. Furthermore, the stereo-structure of **5** was determined by single-crystal X-ray diffraction analysis (Figure 5b). Although compound **5** contains a stereogenic center at C-3, its optical rotation was nearly zero. In addition, no obvious Cotton effect was observed in its ECD spectrum, indicating that it was not enantiomerically pure. Subsequently, various chiral columns and mobile phase systems were attempted to separate enantiomers of **5**; unfortunately, all efforts failed, and it was tentatively designated as a racemic (±)-asperisocoumarin J.

(±)-Asperisocoumarin K (**6**) was isolated as a brown oil. The molecular formula of C_12_H_14_O_5_ was determined based on HRESIMS data detected at *m*/*z* 239.0913 [M + H]^+^ (calcd. for C_12_H_15_O_5_, 239.0914), implying six degrees of unsaturation. The ^1^H NMR and HSQC spectra (Table 3) suggested that there were twelve sp^3^-hybridized protons and one sp^2^-hybridized proton. The ^13^C NMR spectrum indicated twelve carbon atoms, including one carbonyl carbon, five olefinic quaternary carbons, one methine carbon, three methylene carbons, and two methoxy carbons. The presence of a benzene ring was revealed by HMBC correlations (Figure 5a) of H-5 with C-7, C-8a, C-6, C-4a and C-4. The ^1^H-^1^H COSY correlation (Figure 5a) between H-3 and H-4 confirmed that the corresponding carbons were adjacent. The correlations of H-3 with C-4, C-4a and C-1 indicated that one side of C-3 was linked to the benzene ring through CH_2_-4, while the other side was connected to the carbonyl through an oxygen atom. Additionally, a correlation of H-5 with the C-1 carbonyl signal suggested the presence of an oxygen-containing six-membered heterocyclic ring. Moreover, the HMBC spectrum also showed the correlations of H-11 with C-6 and C-5, confirming this methoxy group’s attachment at C-6, which was adjacent to C-5. The only HMBC correlation of the methoxy proton (H-10) with C-9, combined with correlations of H-9 with C-7, C-6 and C-8, suggested that the other methoxy group was connected to the benzene ring via a CH_2_-9 linkage. Therefore, compound **6** was characterized as asperisocoumarin K.

In addition, six known compounds were identified as protubonine B (**7**) [9], protubonine A (**8**) [9], 2,4-dihydroxy-3,5,6-trimethylbenzene (**9**) [10], dankasterone A (**10**) [11], 5-(Undeca-3′,5′,7′-trien-1′-yl)furan-2-ol (**11**) [12], 2,4-Dihydroxy-6-(3*E*,5*E*)-3,5-nonadien-1-ylbenzoic acid (**12**) [13,14] by comparison of their spectroscopic data (Supplementary Materials) with literature values.

### 2.2. Bioactivity Assay

**Figure 6:**
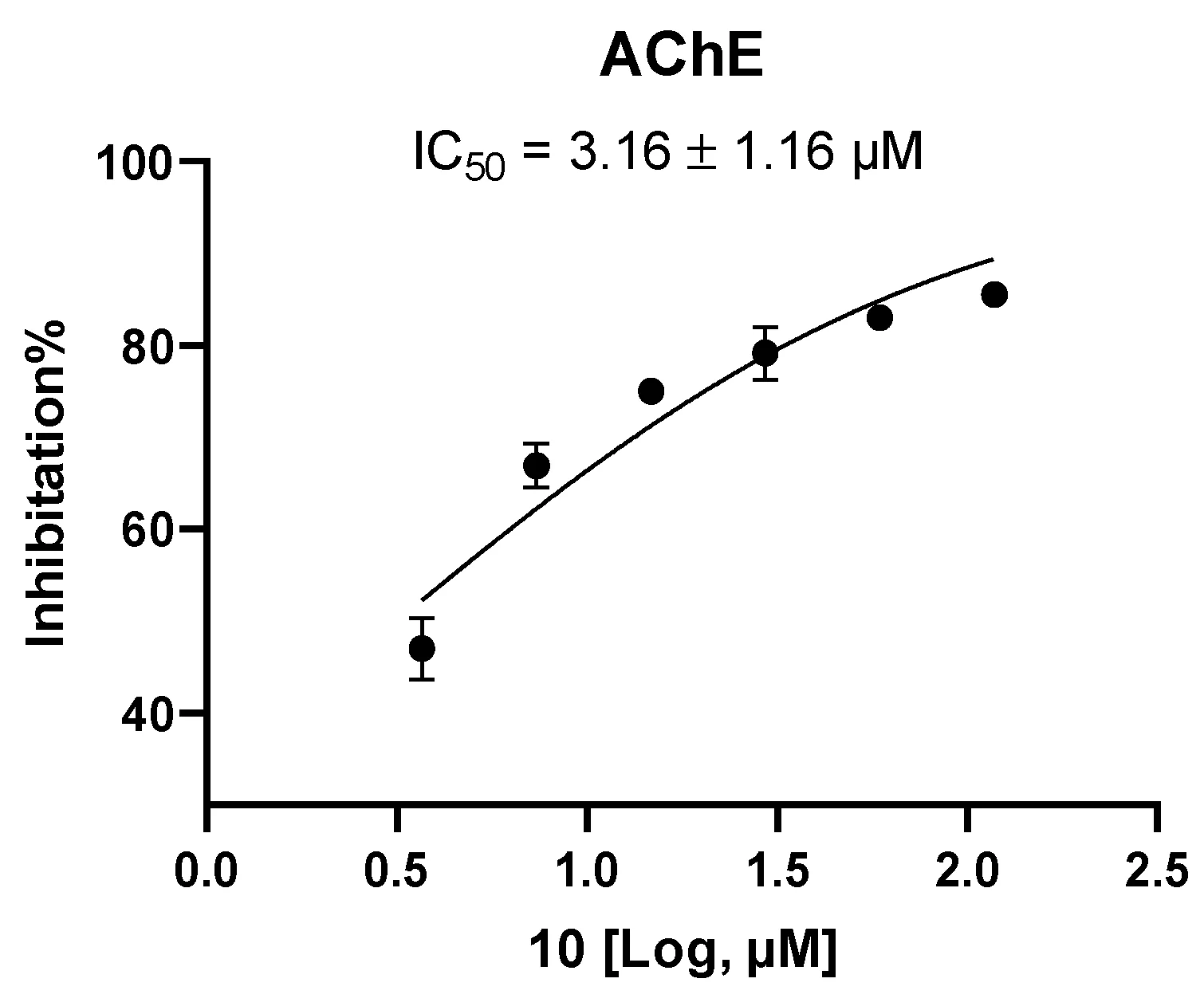
Compound **10** revealed inhibition of enzyme activity of AChE. Inhibition was measured with increasing concentrations of **10**, and IC_50_ value was calculated.

**Figure 7:**
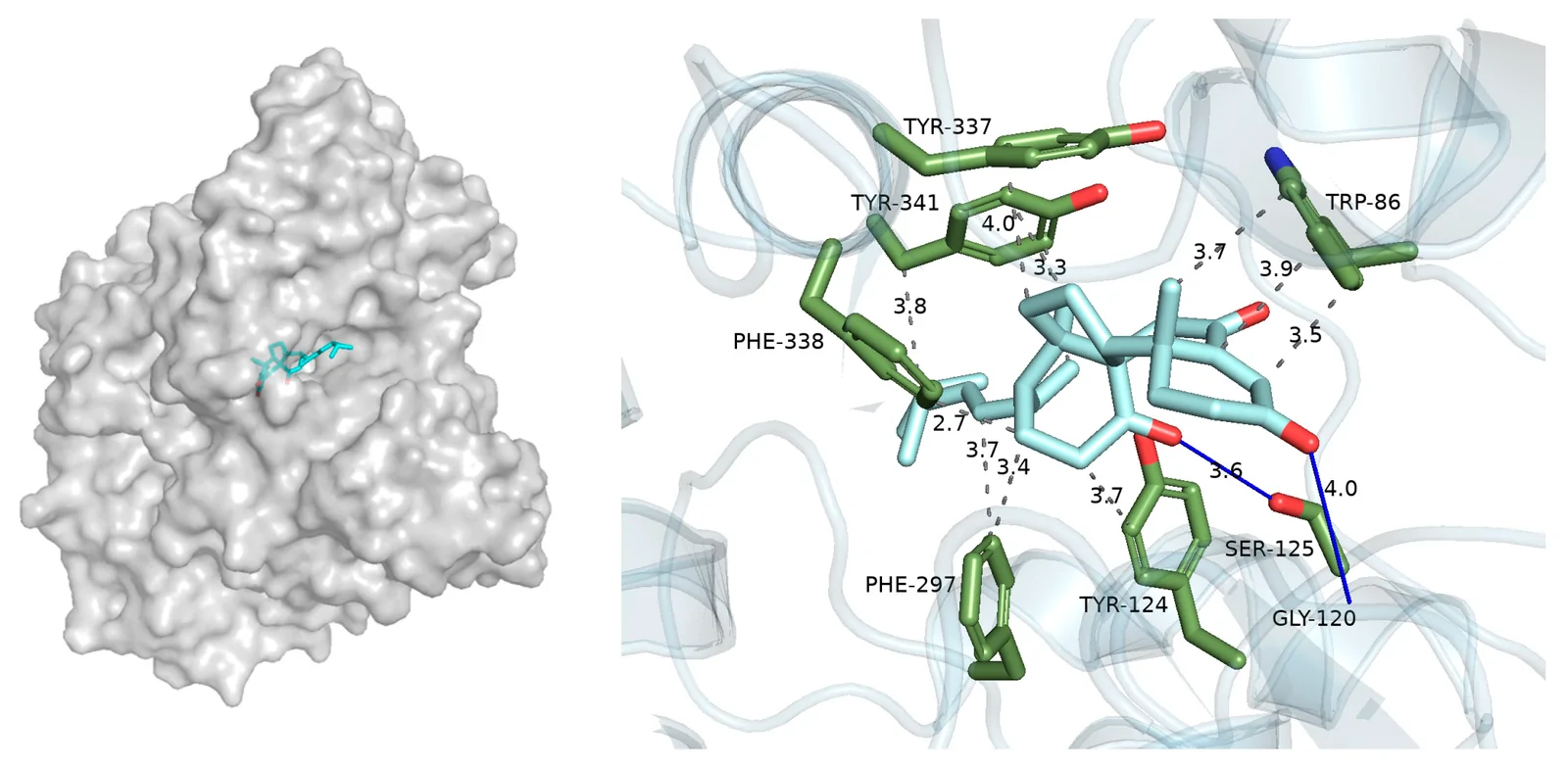
Molecular docking predicted binding interactions of **10** with AChE.

All compounds were screened for their inhibitory activity against acetylcholinesterase. The AChE inhibitors activity was initially screened at a concentration of 50 μg/mL, and the inhibition rate was used as the evaluation criterion. To confirm that the effects of inhibition were ascribed merely to the compounds, the highest concentration of DMSO in the activity assay was diluted to 0.1% (*v*/*v*). An equivalent DMSO concentration was included in the control group. Compounds showing inhibition rates greater than 50% were further subjected to IC_50_ determination. Ultimately, compound **10** was determined as a potential inhibitor of AChE, with the IC_50_ value of 3.16 ± 1.16 μM (Figure 6), while positive control (tacrine) to be 510 ± 29 nM [15]. To investigate the binding mode of **10** with AChE, molecular docking studies were performed (Figure 7). Compound **10** exhibited favorable interactions with the target protein AChE (PDB code: 4EY7), with docking scores ranging from −10.860 to −5.403, while the positive control tacrine showed a docking score of −9.965 [16]. Compound **10** with a docking score of −10.860 formed two hydrogen bonds with the active-site residues GLY-120 and SER-125, with bond distances of 3.4 Å and 3.6 Å, respectively. Additionally, hydrophobic interactions were observed between **10** and the following active-site residues: TRP-86, TYR-124, PHE-297, TYR-337, PHE-338, and TYR-341. Therefore, a favorable binding free energy of **10** with AChE was calculated as −10.860 kcal/mol, suggesting that it possesses a strong binding affinity for this target protein. These results suggested that compound **10** is a promising candidate for further studies as a selective AChE inhibitor.

All compounds were also evaluated for antibacterial and antifungal activities using the disk diffusion method. Compounds exhibiting inhibition zones were subsequently subjected to MIC determination. MIC values were determined by the broth microdilution method, with test compounds and positive controls serially diluted twofold in 96-well plates at final concentrations ranging from 128 to 1 μg/mL. For antibacterial assays, 100 μL of bacterial suspension, 98 μL of LB medium, and 2 μL of compound solution were added to each well of experimental groups, while 2 μL of DMSO was added to the wells of control groups. Antibacterial MIC plates were incubated at 37 °C for 16 h; antifungal MIC plates at 28 °C for 36–48 h. Bacterial growth was monitored by measuring the absorbance at 600 nm. Compound **6** and known compounds **11** and **12** exhibited weak antibacterial activity against *Exiguobacterium aurantiacum*, with MIC values of 0.54, 0.14 and 0.12 mM, respectively. Compounds **11** and **12** also showed weak activity against *Staphylococcus aureus* (*S. aureus*; MIC = 0.28 and 0.12 mM, respectively), methicillin-resistant *Staphylococcus aureus* (MRSA; MIC = 0.14 and 0.12 mM, respectively), and *Enterococcus faecalis* (*E. faecalis*; MIC = 0.28 and 0.23 mM, respectively). For comparison, the positive control ampicillin showed MIC values of less than 2.86 μM against all tested bacteria. In antifungal assays, compound **6** showed weak antifungal activity against *Fusarium oxysporum*, with an MIC of 0.27 mM, while the positive control nystatin showed an MIC of 1.08 μM.

## 3. Materials and Methods

### 3.1. General Experimental Procedures

Thin-layer chromatography (TLC) was conducted on silica gel GF254 under ultraviolet light at 254 nm and 365 nm, and column chromatography (CC) was done on silica gel (100–200 mesh and 200–300 mesh; Qingdao Marine Chemical Factory, Qingdao, China). Semi-preparative high-performance liquid chromatography (HPLC) was carried out on a Hitachi Primaide system equipped with a NanoChrom column (ChromCore 120 C18, 250 × 10 mm I.D., S-5 μm; NanoChrom Technologies Co., Ltd., Suzhou, China) using a diode-array detector (DAD) (Hitachi, Tokyo, Japan). Acetylcholinesterase from fly head and Acetylthiocholine (ATCh) were bought from Shandong Keyuan Biochemical Co., Ltd. (Yantai, China). 5,5′-dithiobis(2-nitrobenzoic acid) (DTNB) and phosphate-buffered saline (PBS, pH 7.4) were purchased from Shanghai Macklin Biochemical Co., Ltd. (Shanghai, China) and Wuhan Servicebio Technology Co., Ltd. (Wuhan, China), respectively.

NMR spectra were recorded on a Bruker Avance III 500 MHz spectrometer (Bruker, Billerica, MA, USA) using tetramethylsilane (TMS) as an internal standard. HRESIMS data were acquired on a Bruker maXis impact Q-TOF mass spectrometer (Bruker, Fallanden, Switzerland). Optical rotations were recorded on a MCP 500 polarimeter (Anton Paar, Graz, Austria). ECD spectra were obtained on a Chirascan CD spectrometer (Applied Photophysics, Leatherhead, Surrey, UK).

### 3.2. Fungal Material

The fungal strain *Aspergillus* sp. SCSIO 41440 was isolated from mangrove sediments collected at the Mangrove Park, Thaladuwa, Negombo, Sri Lanka, and deposited at the Key Laboratory of Tropical Marine Bio-resources and Ecology, South China Sea Institute of Oceanology, Chinese Academy of Sciences (Guangzhou, China). Based on ITS sequence analysis (GenBank accession No. PZ812247), the strain was identified as *Aspergillus* sp., showing 97% sequence similarity to the corresponding region of *Aspergillus puniceus*.

### 3.3. Fermentation and Extraction

The strain was revived and cultivated on potato dextrose agar (PDA) plates at 28 °C for 8 days. Before large-scale fermentation, the fungal strain was cut into small pieces (ca. 1 cm^3^) and inoculated onto solid rice medium, made up of equal weights of rice and distilled water and 3.2% (*w*/*v*) sea salt. The cultures were incubated at 26 °C for 28 days. Finally, ethyl acetate was used to terminate the fermentation and to extract the fermentation products in culture medium, to yield 235.7 g of brown extract.

### 3.4. Isolation and Purification

The crude extract was preliminarily separated by silica gel column chromatography (CC). Using a gradient of petroleum ether (PE)–dichloromethane (DCM) (1:0, 1:1, 0:1, *v*/*v*) and DCM–methanol mixtures (100:1, 100:3, 100:5, 100:7, 10:1, 5:1, 0:1, *v*/*v*), the preliminary separation yielded 28 fractions according to TLC analysis.

Fr.6 was divided into 17 subfractions by ODS silica gel column chromatography eluted with CH_3_OH/H_2_O (10–100%). Fr.6-12 was subdivided by semi-preparative HPLC (80% CH_3_CN/H_2_O, 3.0 mL/min) to obtain **10** (40.9 mg, *t*_R_ 22.9 min).

Fr.7 was subjected to ODS silica gel column chromatography eluted with a gradient of CH_3_OH/H_2_O (10–100%) to afford 20 subfractions (Fr.7-1–Fr.7-20). Fr.7-2 was purified by semi-preparative HPLC (52% CH_3_OH /H_2_O, 3.0 mL/min) to gain **6** (4.8 mg, *t*_R_ 14.4 min). Under semi-preparative HPLC (50% CH_3_CN/H_2_O, 3.0 mL/min), compound **5** (26.8 mg, *t*_R_ 9.5 min) was isolated from Fr.7-5. Then, Fr.7-6 was subdivided by semi-preparative HPLC (30% CH_3_CN/H_2_O/0.04% formic acid, 3.0 mL/min) to gain **8** (15.9 mg, *t*_R_ 12.9 min). Fr.7-7 was separated by semi-preparative HPLC (32% CH_3_CN/H_2_O/0.04% formic acid, 3.0 mL/min) to obtain **9** (10.4 mg, *t*_R_ 12.2 min), and another subfraction Fr.7-7-6 was further purified by HPLC with the same separation conditions except using a naphthyl column to gain **4** (6.5 mg, *t*_R_ 29.8 min) and **7** (330.8 mg, *t*_R_ 27.6 min). Fr.7-12 yielded **11** (26.9 mg, *t*_R_ 28.3 min) via semi-preparative HPLC (85% CH_3_OH /H_2_O, 2.5 mL/min). Fr.7-13 was processed with semi-preparative HPLC (70% recycled CH_3_CN/H_2_O/0.04% formic acid, 3.0 mL/min) to afford **12** (52.3 mg, *t*_R_ 22.3 min).

Fr.11 was fractionated on an ODS gel column to yield 18 subfractions with CH_3_OH/H_2_O (10–100%). **3** (3.8 mg, *t*_R_ 30.6 min), **1** (5.2 mg, *t*_R_ 32.2 min), **2** (3.4 mg, *t*_R_ 34.4 min) were isolated from Fr.11-12 using HPLC with 55% CH_3_CN /H_2_O at the flow rate of 3.0 mL/min.

### 3.5. Spectroscopic Data of Compounds

Aspernidine C (**1**): brown oil; UV(CH_3_OH) *λ*_max_ (log *ε*) 239 (3.38), 214 (3.59) nm; IR (film) *ν*_max_ 3420, 2957, 2920, 2857, 1668, 1647, 1456, 1362, 1086, 772 cm^−1^; HRESIMS *m*/*z* 386.2325 [M + H]^+^ (calcd. for C_23_H_32_NO_4_, 386.2326).

Aspernidine D (**2**): brown oil; ECD (0.78 mM, CH_3_OH) *λ*_max_ (Δ*ε*) 223 (0.47), 204 (−0.49); ${\left[\alpha \right]}_{D}^{25}$ +2.0 (*c* 0.05, CH_3_OH); UV(CH_3_OH) *λ*_max_ (log *ε*) 239 (3.08), 214 (3.32) nm; IR (film) *ν*_max_ 3364, 1645, 1636, 1362, 1087, 679 cm^−1^; HRESIMS *m*/*z* 386.2325 [M + H]^+^ (calcd. for C_23_H_32_NO_4_, 386.2326).

Aspernidine E (**3**): brown oil; UV(CH_3_OH) *λ*_max_ (log *ε*) 239 (3.18), 214 (3.38) nm; IR (film) *ν*_max_ 3381, 1660, 1651, 1362, 1017, 667 cm^−1^; HRESIMS *m*/*z* 386.2325 [M + H]^+^ (calcd. for C_23_H_32_NO_4_, 386.2326).

Protubonine C (**4**): yellow solid; ECD (0.47 mM, CH_3_OH) *λ*_max_ (Δ*ε*) 286 (−1.07), 215 (−12.53), 200 (−0.96) nm; ${\left[\alpha \right]}_{D}^{25}$−159.5 (*c* 0.1, CH_3_OH); UV(CH_3_OH) *λ*_max_ (log *ε*) 277 (3.32), 244 (3.93) nm; IR (film) *ν*_max_ 3404, 2945, 2833, 1748, 1668, 1385, 1227, 1024, 756, 704 cm^−1^; HRESIMS *m*/*z* 456.1537 [M + Na]^+^ (calcd. for C_24_H_23_N_3_NaO_5_, 456.1530).

(±)-Asperisocoumarin J (**5**): pale red amorphous powder; ${\left[\alpha \right]}_{D}^{25}$ 0.0 (*c* 0.1, CH_3_OH); UV(CH_3_OH) *λ*_max_ (log *ε*) 314 (3.87), 270 (4.13), 214 (4.39) nm; IR (film) *ν*_max_ 3420, 1653, 1622, 1350, 1011, 951 cm^−1^; HRESIMS *m*/*z* 253.0719 [M–H]^−^ (calcd. for C_12_H_13_O_6_, 253.0718).

Asperisocoumarin K (**6**): brown oil; UV(CH_3_OH) *λ*_max_ (log *ε*) 304 (3.34), 270 (3.79), 221 (4.08) nm; IR (film) *ν*_max_ 3431, 2941, 1661, 1267, 1155, 1126, 1084, 806, 758 cm^−1^; HRESIMS *m*/*z* 239.0913 [M + H]^+^ (calcd. for C_12_H_15_O_5_, 239.0914).

### 3.6. X-Ray Crystallographic Analysis

Compound **5** was dissolved in methanol, and transparent red needle-shaped crystals were obtained upon slow evaporation of the solvent. The crystallographic information for **5** was submitted to the Cambridge Crystallographic Data Centre (CCDC) and is accessible free of charge upon request from the Centre at 12 Union Road, Cambridge, CB2 1EZ, UK.

Crystal Data for **5**: C_12_H_14_O_6_, *M*r = 254.23, crystal size 0.1 × 0.09 × 0.07 mm^3^, tri-clinic, *a* = 6.4884 (8) Å, *b* = 8.6930(13) Å, *c* = 11.2707(16) Å, *α* = 72.588(13)°, *β* = 80.539(11)°, *γ* = 76.750(12)°, *V* = 587.28(15) Å^3^, *Z* = 2, *T* = 99.9 (5) K, space group P-1, *µ* (Cu Kα) = 0.992 mm^−1^, *Dcalc* = 1.438 g/cm^3^, 6000 reflections measured (8.266° ≤ 2Θ ≤ 148.222°), 2268 unique (*R*_int_ = 0.0532, *R*_sigma_ = 0.0673). The final *R*_1_ was 0.0771 (I > 2*σ*(I)) and *wR*_2_ was 0.2276 (all data). The goodness of fit on F^2^ was 1.079 (CCDC 2571961).

### 3.7. ECD Calculation of Compounds **1** and **4**

For new compounds with chirality, ECD calculations were carried out to determine their absolute configuration. The general procedure comprises four main steps: conformational optimization, conformational analysis, spectrum calculation, and spectral fitting. The initial conformations were generated using the GFN0-xTB method, and were subjected to preliminary optimization at the GFN2-xTB level. All low-energy conformers within an energy window of 4 kcal/mol were further optimized, and were subjected to frequency calculations at the r2SCAN-3c level using the ORCA program, excluding transition states with imaginary frequencies and geometrically redundant structures. Excitation energies and rotational strengths for ECD simulations were calculated at the B3LYP/TZVP level using the SMD implicit solvation model with methanol as solvent. Subsequently, the individual ECD curves and their Boltzmann-weighted spectra for all conformers were generated using Multiwfn 3.8 [17,18].

### 3.8. Enzyme Activity Inhibition Assay

Based on Ellman’s method, enzyme inhibitory activities of the compounds against acetylcholinesterase were evaluated [19]. The optical density (OD) of each well was measured at 405 nm using a microplate reader. For compounds that exhibited more than 50% inhibition of enzyme activity, the IC_50_ values were further determined with six concentration points from 1.56 to 50.00 μg/mL. All inhibition data were collected and analyzed using GraphPad Prism 8.0.2 (GraphPad Dotmatics, Boston, MA, USA). Concentration-response curves were fitted using a normalized nonlinear regression model with the variable slope to obtain the IC_50_ values.

### 3.9. Antimicrobial Activity Assay

The disk diffusion method was employed to evaluate the antibacterial and antifungal activities of all compounds. The tested pathogenic bacteria included six human pathogens: *S. aureus*, MRSA, *E. faecalis*, *Exiguobacterium aurantiacum*, *Klebsiella pneumoniae,* and *Acinetobacter baumannii*. The tested phytopathogenic fungi included four strains: *Fusarium oxysporum*, *Fusarium oxysporum f.* sp. cubense, *Alternaria solani*, and *Alternaria longipes*.

For the preliminary antibacterial screening, a bacterial suspension (OD_600_ = 0.08–0.13) was spread onto agar plates. Filter paper discs (6 mm) containing 2 μL of test compounds or positive controls (ampicillin and gentamicin) were placed on the plates. Bacterial growth was observed and the inhibitory effects were recorded 18 h later. Compounds showing positive results, i.e., exhibiting inhibition zones, were further subjected to MIC determination in 96-well plates [20]. The plates were incubated at 37 °C for 16 h to determine the minimum inhibitory concentration of each compound.

For preliminary antifungal screening, fungal agar blocks were placed on MB medium. Filter paper discs, containing test compounds or positive control (nystatin), were placed around the blocks. After 2–3 days, compounds showing obvious inhibition zones were further evaluated for MIC values using the twofold serial dilution method in 96-well plates [20]. Fungal suspensions were diluted 100-fold, and the compounds and the positive control (25.6 μg/μL) were added. After incubation at 28 °C for 36–48 h, the MIC values were determined.

### 3.10. Molecular Docking

The crystal structure of the receptor protein AChE (PDB code: 4EY7) was acquired from the Protein Data Bank (http://www.rcsb.org/). Water molecules and small-molecule ligands in the crystal structure were removed by using PyMol 2.5. The docking grid box was defined with the center coordinates (9.8355, −58.8919, −24.4105) with a box size of 22 Å in each dimension (x, y, z). Compound **10** as the ligand molecule was preprocessed together with AChE using AutoDockTools 1.5.6, including addition of hydrogen atoms, assignment of Gasteiger charges, and setting of atom types. Molecular docking calculations were then performed with AutoDock Vina 1.2.7 [21]. The intermolecular interactions between **10** and AChE residues in the docked complexes were analyzed via the PLIP online server [22], and final structural visualization and rendering were carried out using PyMol.

## 4. Conclusions

In summary, four new alkaloids (**1**–**4**) and two new polyketides (**5**, **6**), along with six known ones (**7**–**12**) were obtained from the mangrove-derived fungus *Aspergillus* sp. SCSIO 41440. The planar structures of the new metabolites were elucidated by 1D and 2D NMR spectroscopic analysis and HRESIMS. The absolute configuration of **4** was assigned by NOESY, ECD, and optical rotation; the relative configuration of racemic **5** was established by single-crystal X-ray diffraction. All compounds were assessed for their AChE inhibitory activity. Notably, compound **10** exhibited potent AChE inhibition with an IC_50_ value of 3.16 ± 1.16 μM, representing a promising scaffold for the development of AChE-targeted therapeutic agents. In antimicrobial assays, compound **6** displayed weak but selective antibacterial activity against *Exiguobacterium aurantiacum* and antifungal activity against *Fusarium oxysporum*. Additionally, compounds **11** and **12** showed weak antibacterial activity against *Exiguobacterium aurantiacum*, *S. aureus*, MRSA, and *E. faecalis*. These findings expand the chemical diversity of mangrove-derived *Aspergillus* metabolites and highlight compound **10** as a promising AChE inhibitor scaffold (IC_50_ = 3.16 μM). It suggests that this *Aspergillus* strain is a promising source of bioactive leads. Subsequent studies should focus on in vivo efficacy evaluation and mechanisms to fully exploit the pharmacological potential of these mangrove fungal metabolites.

## Figures and Tables

**Table 1:** ^1^H and ^13^C NMR spectroscopic data of **1**–**3** (500, 125 MHz, DMSO-*d*_6_).

Pos.	1		2		3	
	*δ*_C_, Type	*δ*_H_, (*J* in Hz)	*δ*_C_, Type	*δ*_H_, (*J* in Hz)	*δ*_C_, Type	*δ*_H_, (*J* in Hz)
1	170.2, C		170.2, C		170.2, C	
2		8.27, s		8.27, s		8.27, s
3	42.3, CH_2_	4.11, s	42.3, CH_2_	4.11, s	42.3, CH_2_	4.11, s
3a	122.0, C		122.0, C		122.0, C	
4	145.7, C		145.7, C		145.7, C	
5	137.3, C		137.3, C		137.1, C	
6	151.3, C		151.2, C		151.3, C	
7	101.0, CH	6.63, s	101.0, CH	6.63, s	101.0, CH	6.64, s
7a	127.8, C		127.8, C		127.8, C	
1’	70.4, CH_2_	4.01, t (7.3)	70.7, CH_2_	3.97, t (7.5)	70.1, CH_2_	3.94, t (7.9)
2’	38.9, CH_2_	2.47, t (7.4)	39.3, CH_2_	2.41, t (7.4)	32.0, CH_2_	2.48, t (7.8)
3’	132.6, C		131.6, C		131.3, C	
4’	126.2, CH	5.86, d (10.8)	125.5, CH	5.16, t (7.0)	126.5, CH	5.17, t (7.0)
5’	124.8, CH	6.18, dd (15.1, 10.8)	26.1, CH_2_	2.10–2.03, m	25.9, CH_2_	2.09, q (7.4)
6’	138.2, CH	5.45, dd (15.1, 7.9)	39.1, CH_2_	2.03–1.98, m	39.2, CH_2_	1.99, q (7.6)
7’	35.9, CH	2.21–2.09, m	135.6, C		135.6, C	
8’	36.7, CH_2_	1.28, q (7.5)	124.7, CH	5.74, d (10.8)	124.8, CH	5.73, d (10.8)
9’	25.3, CH_2_	1.90, q (7.5)	123.5, CH	6.15, dd (15.1, 10.8)	123.5, CH	6.15, dd (15.2, 10.7)
10’	124.4, CH	5.08, t (6.8)	139.3, CH	5.49, dd (15.2, 6.9)	139.2, CH	5.51, dd (15.2, 6.9)
11’	130.6, C		30.6, CH	2.37–2.23, m	30.7, CH	2.36–2.26, m
12’	25.5, CH_3_	1.64, s	22.8, CH_3_	0.95, d (6.8)	22.4, CH_3_	0.96, d (6.7)
13’	17.5, CH_3_	1.54, s	22.8, CH_3_	0.95, d (6.8)	22.4, CH_3_	0.96, d (6.7)
14’	20.5, CH_3_	0.96, d (6.7)	16.3, CH_3_	1.68, s	16.3, CH_3_	1.67, s
15’	16.5, CH_3_	1.75, s	16.1, CH_3_	1.61, s	23.6, CH_3_	1.69, s

**Table 2:** ^1^H and ^13^C NMR spectroscopic data of **4** (500, 125 MHz, DMSO-*d*_6_).

Pos.	*δ*_C_, Type	*δ*_H_, (*J* in Hz)	Pos.	*δ*_C_, Type	*δ*_H_, (*J* in Hz)
1	165.5, C		11a	56.4, CH	3.56, dd (12.3, 5.1)
2-NH		8.44, s	12	37.2, CH_2_	3.18, dd (13.7, 4.9) 2.99, dd (13.7, 4.9)
3	55.8, CH	4.51, t (4.9)	13	136.1, C	
4	165.1, C		14	128.3, CH	7.32, d (7.5)
5a	79.0, CH	6.38, s	15	130.3, CH	7.22, d (7.5)
6a	144.4, C		16	126.8, CH	7.29–7.25, m
7	117.2, CH	7.92, d (8.0)	17	130.3, CH	7.22, d (7.5)
8	130.4, CH	7.37–7.32, m	18	128.3, CH	7.32, d (7.5)
9	124.5, CH	7.16–7.09, m	19	170.5, C	
10	124.2, CH	7.45, d (8.0)	20	23.7, CH_3_	2.47, s
10a	128.2, C		21	169.4, C	
10b	88.0, C		22	21.1, CH_3_	2.03, s
11	41.5, CH_2_	2.53–2.51, m 1.13, t (12.3)			

**Table 3:** ^1^H and ^13^C NMR spectroscopic data of **5** and **6** (500, 125 MHz, DMSO-*d*_6_).

Pos.	5		6	
	*δ*_C_, Type	*δ*_H_, (*J* in Hz)	*δ*_C_, Type	*δ*_H_, (*J* in Hz)
1	170.4, C		169.5, C	
3	106.7, C		67.7, CH_2_	4.54, t (6.2)
4	33.8, CH_2_	3.27, s 3.03, dd (16.8, 0.8)	27.2, CH_2_	3.05, t (6.0)
4a	132.3, C		143.0, C	
5	139.4, C		102.0, CH	6.63, s
6	159.8, C		163.8, C	
7	102.8, CH	6.27, d (0.7)	110.9, C	
8	161.6, C		161.2, C	
8a	100.1, C		101.9, C	
9	23.0, CH_3_	1.66, s	61.2, CH_2_	4.36, s
10	50.5, CH_3_	3.35, s	57.3, CH_3_	3.20, s
11	61.2, CH_3_	3.70, s	56.2, CH_3_	3.87, s

## Data Availability

The original contributions presented in this study are included in the article. Further inquiries can be directed to the corresponding author.

## Supplementary Materials

The following supporting information can be downloaded at: https://www.mdpi.com/article/10.3390/md24090297/s1, Figures S1–S48: NMR, HRESIMS, IR and UV spectra of **1**–**6**; Figures S49–S60: ^1^H NMR and ^13^C NMR data of compounds **7**–**12** and comparison with literature values [9,10,11,12,13,14].
