# Supplementary material for: New Prenylated Isoindolinone Alkaloids and Polyketides from the Mangrove-Derived Fungus Aspergillus sp. SCSIO 41440
Source: Mar Drugs. 2026 Aug 25;24(9):297. doi: 10.3390/md24090297 (PMC13608482; doi:10.3390/md24090297)
Supplement: Supplementary file 1 [file marinedrugs-24-00297-s001.zip › marinedrugs-4488285-supplementary.pdf]

SUPPLEMENTARY MATERIAL

New Prenylated Isoindolinone Alkaloids and Polyketides from the Mangrove-Derived Fungus Aspergillus sp. SCSIO 41440

Chun yang [1#], Yi Chen [2,3#], Zhifei Xiong [1], Jian Cai [2], Chunmei Chen [4], Bingling Liu [1], Yonghong Liu [2,3], Xuefeng Zhou [2,3] and Huaming Tao [1*]

1 School of Traditional Chinese Medicine, Southern Medical University, Guangzhou 510515, China; 3201008013@smu.edu.cn (C.Y.); xiongzhifei@i.smu.edu.cn (Z.X.); qazwsx256930@163.com (B.L.); taohm@smu.edu.cn (H.T.)

2 State Key Laboratory of Tropical Oceanography/Guangdong Key Laboratory of Marine Materia Medica, South China Sea Institute of Oceanology, Chinese Academy of Sciences, Guangzhou 510301, China; 13620281931@163.com (Y.C.); xfzhou@scsio.ac.cn (X.Z.); yonghongliu@scsio.ac.cn (Y.L.); caijian@scsio.ac.cn (J.C.)

3 University of Chinese Academy of Sciences, Beijing 100049, China; 13620281931@163.com (Y.C.); xfzhou@scsio.ac.cn (X.Z.); yonghongliu@scsio.ac.cn (Y.L.)

4 School of Chemistry, South China Normal University, Guangzhou 510006, China. chenchunmei18@mails.ucas.ac.cn (C.C.)

* Correspondence: taohm@smu.edu.cn (H.T.)

\# C.Y., Y.C. contributed equally to this work.

Contents

1 The ITS gene sequence data of *Aspergillus* sp. SCSIO 41440 4

2 The physicochemical data of the known compounds **7–12** 4

3 1D and 2D NMR, HRESIMS, IR and UV spectra of compounds **1–6** 6

Figure S1. ^{1}H NMR spectrum (500 MHZ) of aspernidine C (**1**) in DMSO-d_6. 6

Figure S2. ^{13}C NMR spectrum (125 MHZ) of aspernidine C (**1**) in DMSO-d_6. 6

Figure S3. HSQC spectrum (500/125 MHZ) of aspernidine C (**1**) in DMSO-d_6. 7

Figure S5. HRESIMS spectra of aspernidine C (**1**)... 8

Figure S6. IR spectrum of aspernidine C (**1**). 9

Figure S7. UV spectrum of aspernidine C (**1**) in CH_3OH. 9

Figure S8. ^{1}H NMR spectrum (500 MHZ) of aspernidine D (**2**) in DMSO-d_6. 10

Figure S9. ^{13}C NMR spectrum (125 MHZ) of aspernidine D (**2**) in DMSO-d_6. 10

Figure S10. HSQC spectrum (500/125 MHZ) of aspernidine D (**2**) in DMSO-d_6. 11

Figure S11. HMBC spectrum (500/125 MHZ) of aspernidine D (**2**) in DMSO-d_6. 11

Figure S12. HRESIMS spectra of aspernidine D (**2**). 12

Figure S13. IR spectrum of aspernidine D (**2**). 13

Figure S14. UV spectrum of aspernidine D (**2**) in CH_3OH. 13

Figure S15. ^{1}H NMR spectrum (500 MHZ) of aspernidine E (**3**) in DMSO-d_6. 14

Figure S16. ^{13}C NMR spectrum (125 MHZ) of aspernidine E (**3**) in DMSO-d_6. 14

Figure S17. HSQC spectrum (500/125 MHZ) of aspernidine E (**3**) in DMSO-d_6. 15

Figure S18. HMBC spectrum (500/125 MHZ) of aspernidine E (**3**) in DMSO-d_6. 15

Figure S19. HRESIMS spectra of aspernidine E (**3**). 16

Figure S20. IR spectrum of aspernidine E (**3**). 17

Figure S21. UV spectrum of aspernidine E (**3**) in CH_3OH. 17

Figure S22. ^{1}H NMR spectrum (500 MHZ) of 11 (**4**) in DMSO-d_6. 18

Figure S23. ^{13}C NMR spectrum (125 MHZ) of protubonine C (**4**) in DMSO-d_6. 18

Figure S24. HSQC spectrum (500/125 MHZ) of protubonine C (**4**) in DMSO-d_6. 19

Figure S25. HMBC spectrum (500/125 MHZ) of protubonine C (**4**) in DMSO-d_6. 19

Figure S26. ^1H–^1H COSY spectrum (500 MHZ) of protubonine C (**4**) in DMSO-d_6. ...20

Figure S27. NOESY spectrum (700 MHZ) of protubonine C (**4**) in DMSO-d_6. 20

Figure S28. HRESIMS spectra of protubonine C (**4**). 21

Figure S29. IR spectrum of protubonine C (**4**). 22

Figure S30. UV spectrum of protubonine C (**4**) in CH_3OH. 22

Figure S31. ^1H NMR spectrum (500 MHZ) of (±)-asperisocoumarin J (**5**) in CD_3OD. 23

Figure S32. ^{13}C NMR spectrum (125 MHZ) of (±)-asperisocoumarin J (**5**) in CD_3OD. 23

Figure S33. HSQC spectrum (500/125 MHZ) of (±)-asperisocoumarin J (**5**) in CD_3OD. 24

Figure S34. HMBC spectrum (500/125 MHZ) of (±)-asperisocoumarin J (**5**) in CD_3OD. 24

Figure S35. ^1H–^1H COSY spectrum (500 MHZ) of (±)-asperisocoumarin J (**5**) in CD_3OD. 25

Figure S36. NOESY spectrum (700 MHZ) of (±)-asperisocoumarin J (**5**) in CD_3OD...25

Figure S37. HRESIMS spectra of (±)-asperisocoumarin J (**5**). 26

Figure S38. IR spectrum of (±)-asperisocoumarin J (**5**). 27

Figure S39. UV spectrum of (±)-asperisocoumarin J (**5**) in CH_3OH. 27

Figure S40. ^1H NMR spectrum (500 MHZ) of asperisocoumarin K (**6**) in DMSO-d_6. .28

Figure S41. ^{13}C NMR spectrum (125 MHZ) of asperisocoumarin K (**6**) in DMSO-d_6. .28

Figure S42. HSQC spectrum (500/125 MHZ) of asperisocoumarin K (**6**) in DMSO-d_6. 29

Figure S43. HMBC spectrum (500/125 MHZ) of asperisocoumarin K (**6**) in DMSO-d_6. 29

Figure S44. ^1H–^1H COSY spectrum (500 MHZ) of asperisocoumarin K (**6**) in DMSO-d_6. 30

Figure S45. NOESY spectrum (700 MHZ) of asperisocoumarin K (**6**) in DMSO-d_6....30

Figure S46. HRESIMS spectra of asperisocoumarin K (**6**). 31

Figure S47. IR spectrum of asperisocoumarin K (**6**). 32

Figure S48. UV spectrum of asperisocoumarin K (**6**) in CH_3OH. 32

4 ^1H and ^{13}C NMR spectra of compounds **7–12** 33

Figure S49. ^1H NMR spectrum (500 MHZ) of protubonine B (**7**) in DMSO-d_6. 33

Figure S50. ^{13}C NMR spectrum (125 MHZ) of protubonine B (**7**) in DMSO-d_6. 33

Figure S51. ^1H NMR spectrum (500 MHZ) of protubonine A (**8**) in DMSO-d_6. 34

Figure S52. ^{13}C NMR spectrum (125 MHZ) of protubonine A (**8**) in DMSO-d_6. 34

Figure S53. ^1H NMR spectrum (500 MHZ) of 2,4-dihydroxy-3,5,6-trimethylbenzene (**9**) in DMSO-d_6. 35

Figure S54. ^{13}C NMR spectrum (125 MHZ) of 2,4-dihydroxy-3,5,6-trimethylbenzene (**9**) in DMSO-d_6. 35

Figure S55. ^1H NMR spectrum (500 MHZ) of dankasterones A (**10**) in DMSO-d_6. 36

Figure S56. ^{13}C NMR spectrum (125 MHZ) of dankasterones A (**10**) in DMSO-d_6. 36

Figure S57. ^1H NMR spectrum (500 MHZ) of 5-(Undeca-3',5',7'-trien-1'-yl) furan-2-ol (**11**) in DMSO-d_6. 37

Figure S58. ^{13}C NMR spectrum (125 MHZ) of 5-(Undeca-3',5',7'-trien-1'-yl) furan-2-ol (**11**) in DMSO-d_6. 37

Figure S59. ^1H NMR spectrum (500 MHZ) of 2,4-dihydroxy-6-((3*E*,5*E*)-nona-3,5-dien-1-yl) benzoic acid (**12**) in DMSO-d_6. 38

Figure S60. ^{13}C NMR spectrum (125 MHZ) of 2,4-dihydroxy-6-((3*E*,5*E*)-nona-3,5-dien-1-yl) benzoic acid (**12**) in DMSO-d_6. 38

Reference for Supporting Information 39

1 The ITS gene sequence data of *Aspergillus* sp. SCSIO 41440

ATATGCTTAAGTTCAGCGGGTATCCCTACCTGATCCGAGGTCAACCTGGTAAA ATAAAAGGTTTGGAGACGCCGGCTGGCGCCCGGCCGGCCCTAAACGAGCGG GTGACAAAGCCCCATACGCTCGAGGACCAGGACGCGGTGCCGCCGCTGCCTT TCGGGCCCGTCCCCCGGGGGGGACGACGACCCAACACACAAGCCGGGCTTG AAGGGCAGCAATGACGCTCGGACAGGCATGCCCCCCGGAATGCCAGGGGGC GCAATGTGCGTTCAAAGACTCGATGATTCACTGAATTCTGCAATTCACATTACT TATCGCAGTTCGCTGCGTTCTTCATCGATGCCGGAACCAAGAGATCCATTGTT GAAAGTTTTAATTAGGTTTGCTTGTATCAAGCTCAGACGACACGCTAAAAACA AGAAGTTCGGTGTGGTCCCCGGCGGCTTACGCCCCCCGGGAAGGGGGCGCGC ACCGCCGAAGCAACGTTGGTCAGGTATTCACGGGTGGGAGGTTAGGCCTGCC TGTGGGGCGAGGCCTGCACTCGGTAATGATCCTTCCGCAGGTTCACCTACGGA AACCTTGTTACATTT

2 The physicochemical data of the known compounds 7–12.

protubonine B (**7**) [1]: ^{1}H NMR (500 MHz, DMSO-*d*$_6$) δ_H 8.21 (1H, s, NH), 7.87 (1H, d, *J* = 8.1 Hz, H-7), 7.62 (1H, dd, *J* = 7.6, 1.3 Hz, H-10), 7.39 (1H, ddd, *J* = 8.3, 7.5, 1.4 Hz, H-8), 7.20 (1H, td, *J* = 7.5, 1.1 Hz, H-9), 6.45 (1H, s, H-5a), 4.07 (1H, m, H-3), 3.93 (1H, dd, *J* = 11.8, 5.7 Hz, H-11a), 3.03 (1H, dd, *J* = 12.5, 6.0 Hz, H-11), 2.62 (1H, t, *J* = 12.1 Hz, H-11), 2.54 (3H, s, H-17), 1.99 (3H, s, 18-CH_3), 1.86 (1H, m, H-12), 1.77 (1H, m, H-13), 1.41 (1H, m, H-12), 0.87 (3H, d, *J* = 6.5 Hz, H-15), 0.84 (3H, d, *J* = 6.5 Hz, H-14); ^{13}C NMR (125 MHz, DMSO-*d*$_6$) δ_C 170.9 (C, C-16), 169.4 (C, C-18), 168.1 (C, C-1), 167.4 (C, C-4), 144.3 (C, C-6a), 130.6 (CH, C-8), 128.7 (C, C-10a), 124.6 (2CH, C-9, 10), 118.4 (CH, C-7), 87.4 (C, C-10b), 79.1 (CH, C-5a), 57.3 (CH, C-11a), 52.5 (CH, C-3), 38.2 (CH_2, C-11), 38.1 (CH_2, C-12), 23.9 (CH, C-13), 23.5 (CH_3, C-17), 22.9 (CH_3, C-14), 21.8 (CH_3, 18-CH_3), 21.1 (CH_3, C-15).

protubonine A (**8**) [1]: ^{1}H NMR (500 MHz, DMSO-*d*$_6$) δ_H 7.89 (1H, d, *J* = 8.0 Hz, H-7), 7.54 (1H, dd, *J* = 7.6, 1.3 Hz, H-10), 7.34 (1H, td, *J* = 7.8, 1.4 Hz, H-8), 7.18 (1H, td, *J* = 7.5, 1.1 Hz, H-9), 5.83 (1H, s, H-5a), 4.00 (1H, ddd, *J* = 7.6, 4.8, 1.3 Hz, H-3), 3.89 (1H, dd, *J* = 11.8, 6.1 Hz, H-11a), 2.74 (1H, dd, *J* = 12.4, 6.1 Hz, H-11), 2.51 (3H, s, H-17), 2.37 (1H, t, *J* = 12.1 Hz, H-11), 1.82 (1H, m, H-12), 1.74 (1H, m, H-13), 1.37 (1H, m, H-12), 0.85 (3H, d, *J* = 6.5 Hz, H-14/15), 0.82 (3H, d, *J* = 6.5 Hz, H-14/15); ^{13}C NMR (125 MHz, DMSO-*d*$_6$) δ_C 170.6 (C, C-16), 168.7 (C, C-1), 167.8 (C, C-4), 142.8 (C, C-6a), 133.5 (C, C-10a), 129.6 (CH, C-8), 124.6 (CH, C-9), 123.9 (CH, C-10), 118.3 (CH, C-7), 82.7 (CH, C-10b), 81.6 (CH, C-5a), 58.5 (CH, C-11a), 52.3 (CH, C-3), 38.4 (CH_2, C-11), 38.0 (CH_2, C-12), 23.9 (CH, C-13), 23.8 (CH_3, C-17), 22.9 (CH_3, C-14/15), 21.8 (CH_3, C-14/15).

2,4-dihydroxy-3,5,6-trimethylbenzene (**9**) [2]: ^{1}H NMR (500 MHz, DMSO-*d*$_6$) δ_H 6.17 (1H, s,

H-1), 2.05 (3H, s, 6-CH_3), 1.96 (3H, s, 3-CH_3), 1.94 (3H, s, 5-CH_3); ^{13}C NMR (125 MHz, DMSO-d_6) δ_C 153.4 (C, C-4), 153.0 (C, C-2), 133.3 (C, C-6), 113.4 (C, C-5), 108.3 (CH, C-1), 108.2 (C, C-3), 20.0 (CH_3, 6-CH_3), 11.8 (CH_3, 5-CH_3), 9.2 (CH_3, 3-CH_3).

dankasterones A (**10**) [3]**:** ^{1}H NMR (500 MHz, DMSO-d_6) δ_H 5.95 (1H, s, H-4), 5.29 (1H, d, *J* = 6.8 Hz, H-23), 5.28 (1H, d, *J* = 6.8 Hz, H-22), 2.77 (1H, td, *J* = 9.0, 1.5 Hz, H-9), 2.66 (1H, d, *J* = 17.6 Hz, H-7), 2.63 (1H, m, H-2), 2.58 – 2.51 (2H, overlapped, H-7, H-2), 2.48 (1H, s, H-15), 2.46 – 2.37 (2H, overlapped, H-15, H-20), 2.29 (1H, dt, *J* = 16.7, 4.3 Hz, H-1), 2.03 – 1.95 (2H, overlapped, H-1, H-11), 1.91 – 1.77 (6H, m, H-16, H-24, H-11, H-12), 1.68 (1H, d, *J* = 12.9 Hz, H-12), 1.65 – 1.58 (1H, m, H-16), 1.55 (1H, dd, *J* = 12.8, 5.4 Hz, H-17), 1.51 – 1.39 (1H, octet, *J* = 6.1 Hz, H-25), 1.20 (3H, s, H_3-19), 1.07 (3H, d, *J* = 6.9 Hz, H_3-21), 0.88 (3H, d, *J* = 6.9 Hz, H_3-18), 0.87 (3H, s, H_3-28), 0.81 (3H, d, *J* = 6.7 Hz, H_3-27), 0.79 (3H, d, *J* = 6.7 Hz, H_3-26); ^{13}C NMR (125 MHz, DMSO-d_6) δ_C 215.2 (C, C-14), 200.0 (C, C-6), 199.0 (C, C-3), 157.4 (C, C-5), 133.7 (CH, C-23), 132.9 (CH, C-22), 124.6 (CH, C-4), 62.1 (C, C-8), 54.2 (C, C-13), 48.8 (CH, C-9), 46.4 (CH, C-17), 42.4 (CH, C-24), 40.3 (CH_2, C-7α), 38.2 (CH_2, C-1α), 37.5 (CH_2, C-12α), 36.7 (CH_2, C-12α), 36.4 (CH, C-20), 35.8 (C, C-10), 34.0 (CH_2, C-2α), 32.5 (CH, C-25), 24.5 (CH_2, C-11α), 23.5 (CH_3, C-19), 23.3 (CH_3, C-21), 23.2 (CH_2, C-16α), 19.9 (CH_3, C-27), 19.5 (CH_3, C-26), 17.3 (CH_3, C-28), 16.4 (CH_3, C-18).

5-(Undeca-3',5',7'-trien-1'-yl) furan-2-ol (**11**) [4]: ^{1}H NMR (500 MHz, DMSO-d_6) δ_H 6.03 (1H, br s, H-4), 6.02 (1H, d, *J* = 2.0 Hz, H-3), 6.01 (4H, m, H-4′, 5′, 6′, 7′), 5.56 (2H, m, H-3′, 8′), 2.44 (2H, m, H-1′), 2.25 (2H, q, *J* = 7.2 Hz, H-2′), 1.99 (2H, q, *J* = 7.2 Hz, H-9′), 1.35 (2H, q, *J* = 7.3 Hz, H-10′), 0.85 (3H, t, *J* = 7.4 Hz, H-11′); ^{13}C NMR (175 MHz, DMSO-d_6) δ_C 158.1 (C, C-2), 143.5 (C, C-5), 132.1 (CH, C-8′), 131.4 (CH, C-3′), 130.6 (2CH, C-4′, 7′), 130.5 (2CH, C-5′, 6′), 106.3 (CH, C-4), 100.0 (CH, C-3), 35.3 (CH_2, C-1′), 34.1 (CH_2, C-9′), 33.7 (CH_2, C-2′), 22.1 (CH_2, C-10′), 13.6 (CH_3, C-11′).

2,4-dihydroxy-6-((3*E*,5*E*)-nona-3,5-dien-1-yl) benzoic acid (**12**) [5, 6]: ^{1}H NMR (500 MHz, DMSO-d_6) δ_H 6.15 (m, 2H, H-3, 5), 5.98 (m, 2H, H-10, 11), 5.56 (m, 2H, H-9, 12), 2.84 (m, 2H, H-7), 2.24 (q, *J* = 7.4 Hz, 2H, H-8), 1.99 (q, *J* = 7.2 Hz, 2H, H-13), 1.34 (m, 2H, H-14), 0.85 (t, *J* = 7.4 Hz, 3H, H-15); ^{13}C NMR (125 MHz, DMSO-d_6) δ_C 172.7 (C-16), 163.8 (C-4), 161.6 (C-2), 146.4 (C-6), 132.2 (C-9), 131.5 (C-12), 130.5 (C-10), 130.5 (C-11), 110.1 (C-5), 105.3 (C-1), 100.8 (C-3), 35.4 (C-13), 34.3 (C-8), 34.2 (C-7), 22.1 (C-14), 13.6 (C-15).

3 [1]D and [2]D NMR, HRESIMS, IR and UV spectra of compounds 1–6

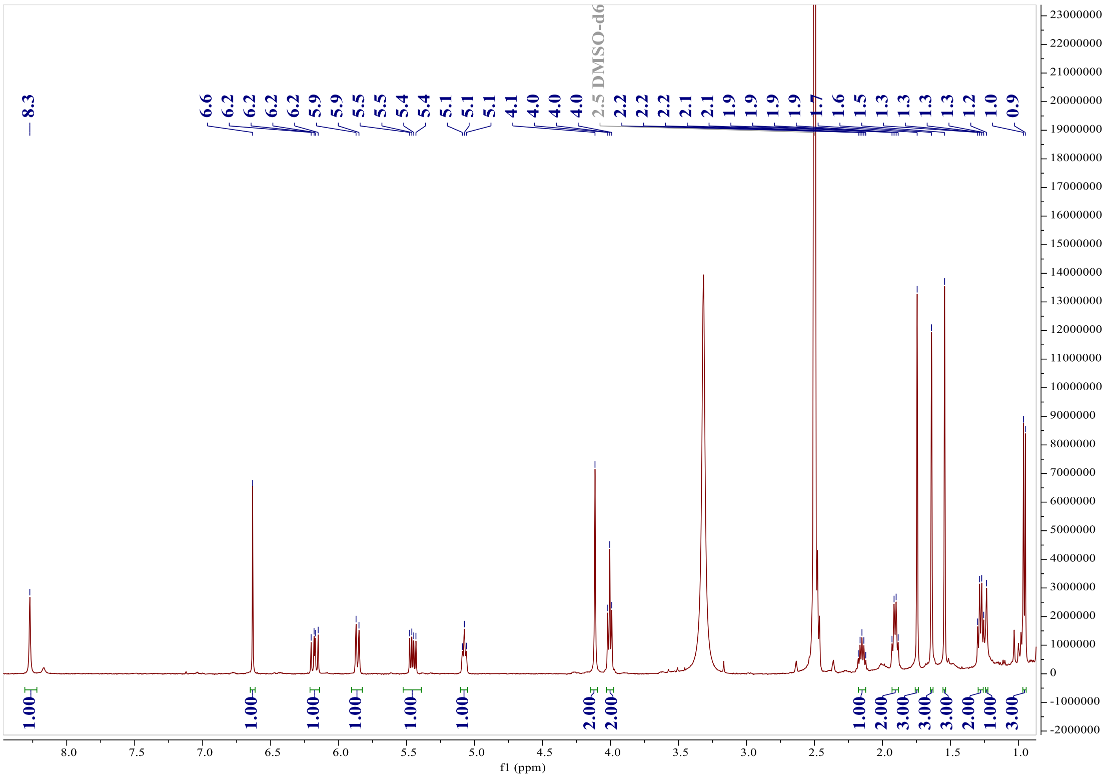

Figure S1. ^{1}H NMR spectrum (500 MHZ) of aspernidine C (**1**) in DMSO-d_6.

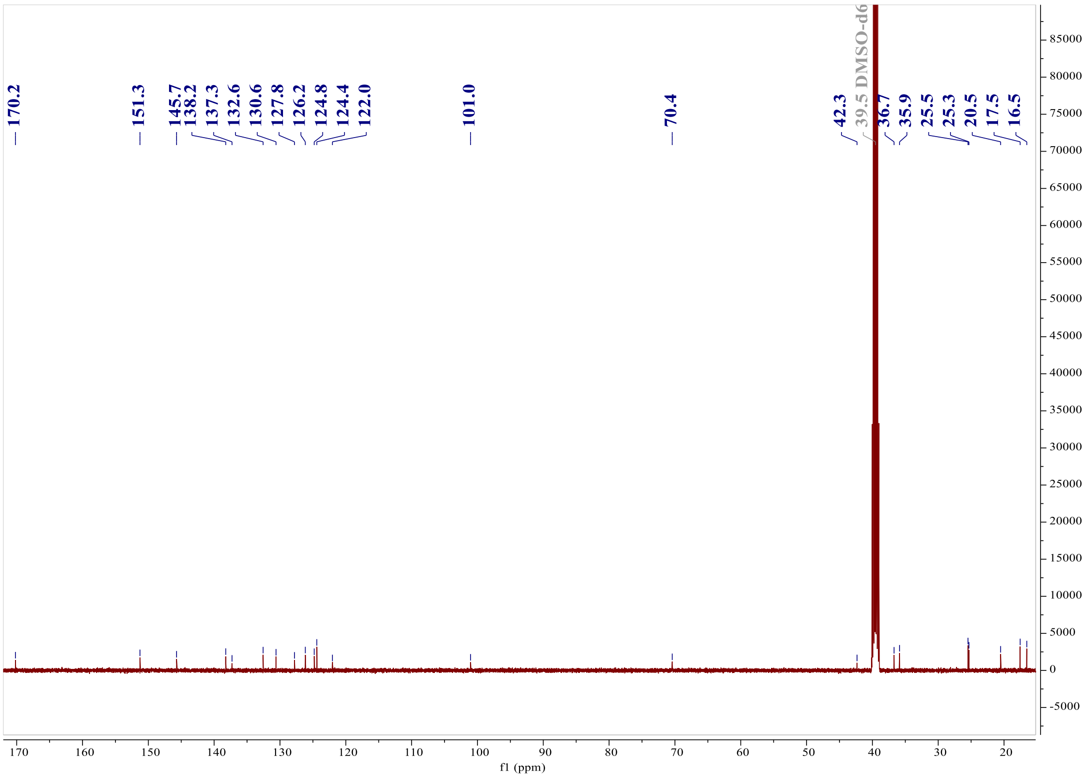

Figure S2. ^{13}C NMR spectrum (125 MHZ) of aspernidine C (**1**) in DMSO-d_6.

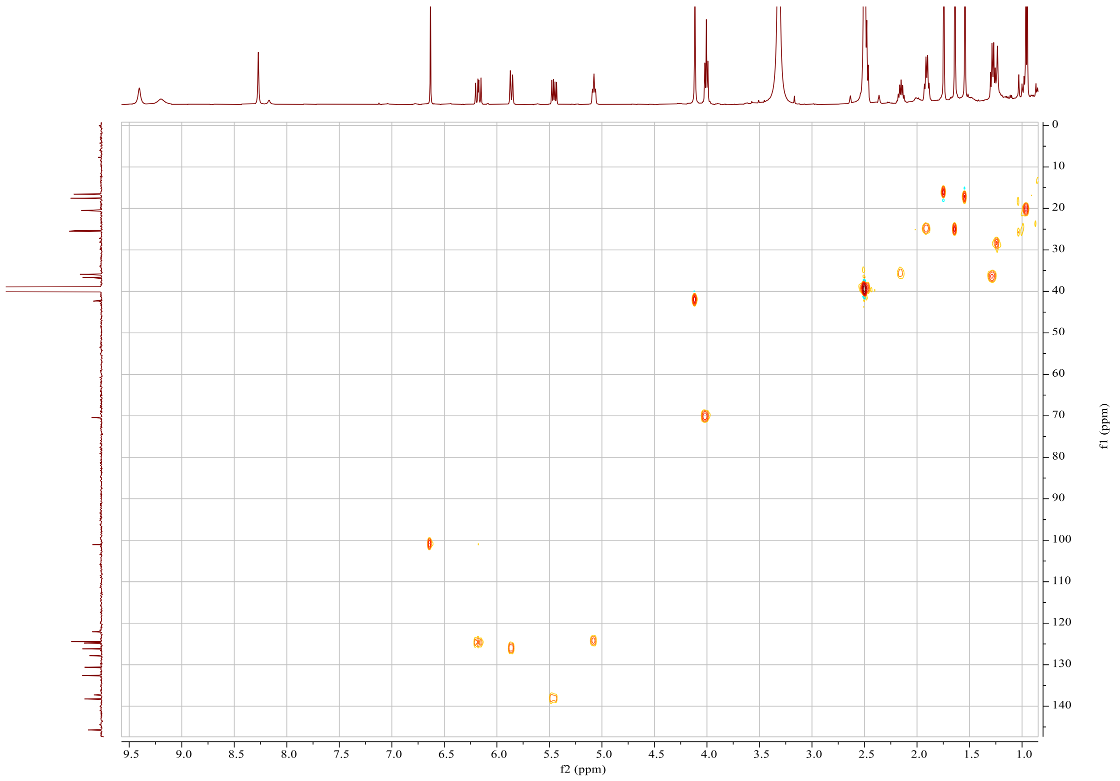

Figure S3. HSQC spectrum (500/125 MHZ) of aspernidine C (**1**) in DMSO-*d*6.

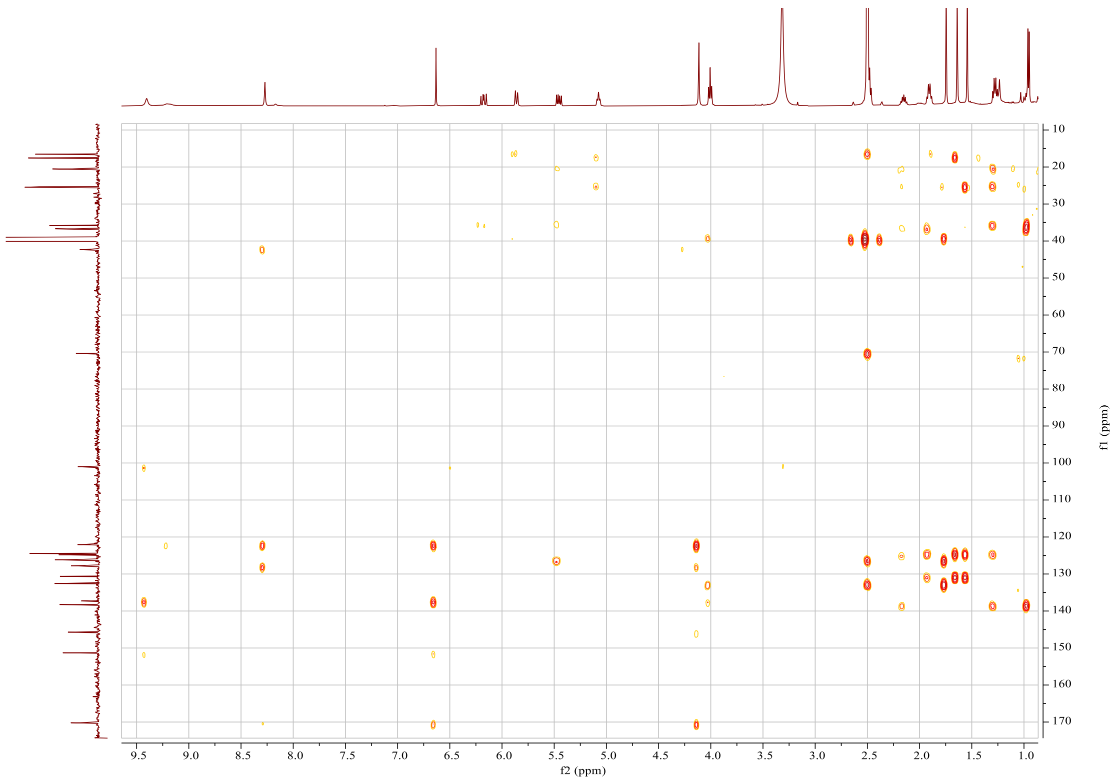

Figure S4. HMBC spectrum (500/125 MHZ) of aspernidine C (**1**) in DMSO-*d*6.

Generic Display Report

Analysis Info			
Analysis Name	D:\Data\MS\data\202603\yangchun_L-4Y64_pos_4_01_21379.d	Acquisition Date	3/17/2026 11:40:59 AM
Method	LC_Direct Infusion_pos_70-500mz.m	Operator	SCSIO
Sample Name	yangchun_L-4Y64_pos	Instrument	maXis
Comment			

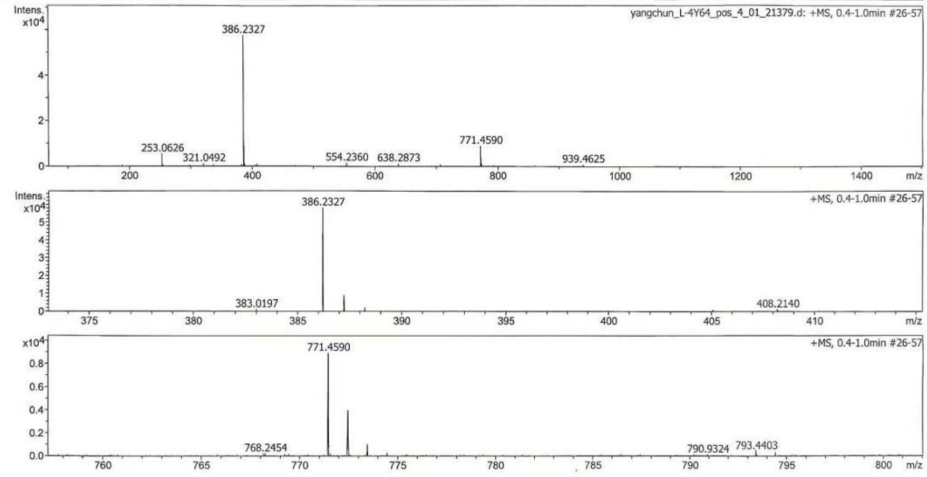

Bruker Compass DataAnalysis 4.1 printed: 3/17/2026 11:53:34 AM by: SCSIO Page 1 of 1

Mass Spectrum SmartFormula Report

Analysis Info				
Analysis Name	D:\Data\MS\data\202603\yangchun_L-4Y64_pos_4_01_21379.d	Acquisition Date	3/17/2026 11:40:59 AM	
Method	LC_Direct Infusion_pos_70-500mz.m	Operator	SCSIO	
Sample Name	yangchun_L-4Y64_pos	Instrument	maXis	255552.00029
Comment				

Acquisition Parameter					
Source Type	ESI	Ion Polarity	Positive	Set Nebulizer	0.4 Bar
Focus	Active	Set Capillary	4500 V	Set Dry Heater	180 °C
Scan Begin	70 m/z	Set End Plate Offset	-500 V	Set Dry Gas	4.0 l/min
Scan End	1500 m/z	Set Charging Voltage	0 V	Set Divert Valve	Waste
		Set Corona	0 nA	Set APCI Heater	0 °C

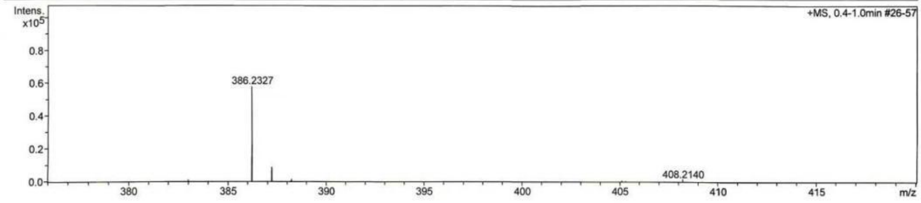

Meas. m/z	#	Ion Formula	Score	m/z	err [ppm]	err [mDa]	mSigma	rdb	e⁻ Conf	N-Rule
386.2327	1	C23H32NO4	100.00	386.2326	-0.4	-0.1	53.3	8.5	even	ok
408.2140	1	C23H31NNaO4	100.00	408.2145	-1.2	-0.5	29.3	8.5	even	ok
771.4590	1	C46H63N2O8	100.00	771.4579	-1.4	-1.1	32.6	16.5	even	ok
793.4403	1	C46H62N2NaO8	100.00	793.4398	-0.6	-0.5	41.8	16.5	even	ok

yangchun_L-4Y64_pos_4_01_21379.d
Bruker Compass DataAnalysis 4.1 printed: 3/17/2026 11:53:04 AM by: SCSIO Page 1 of 1

Figure S5. HRESIMS spectra of aspernidine C (**1**).

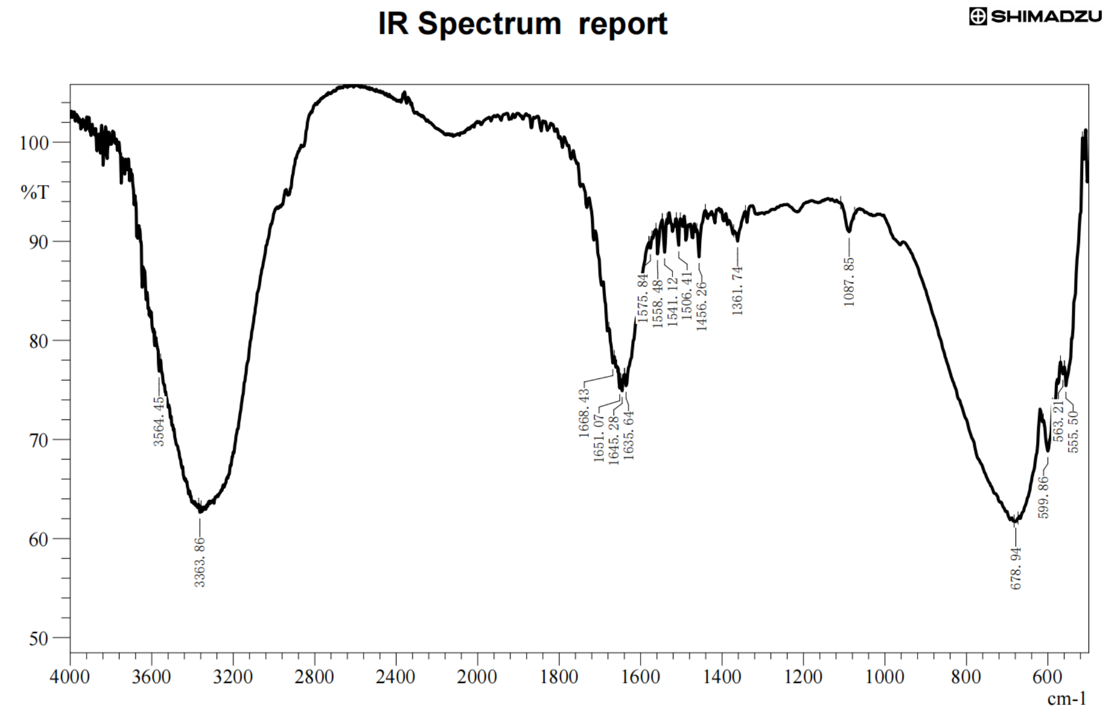

Figure S6. IR spectrum of aspernidine C (**1**).

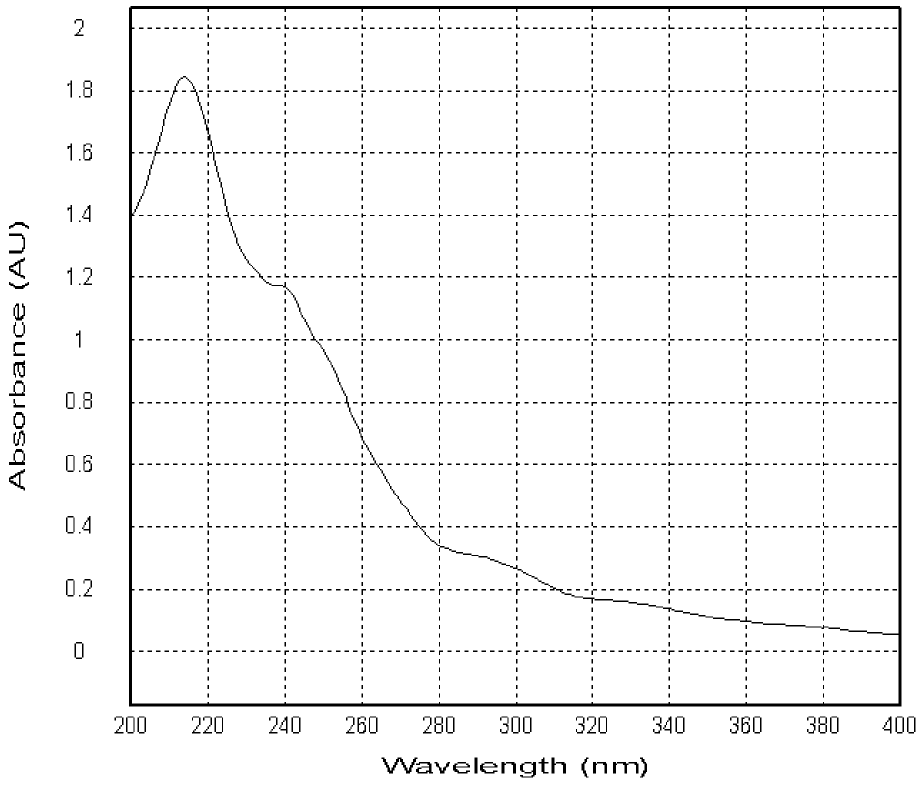

Figure S7. UV spectrum of aspernidine C (**1**) in CH_3OH.

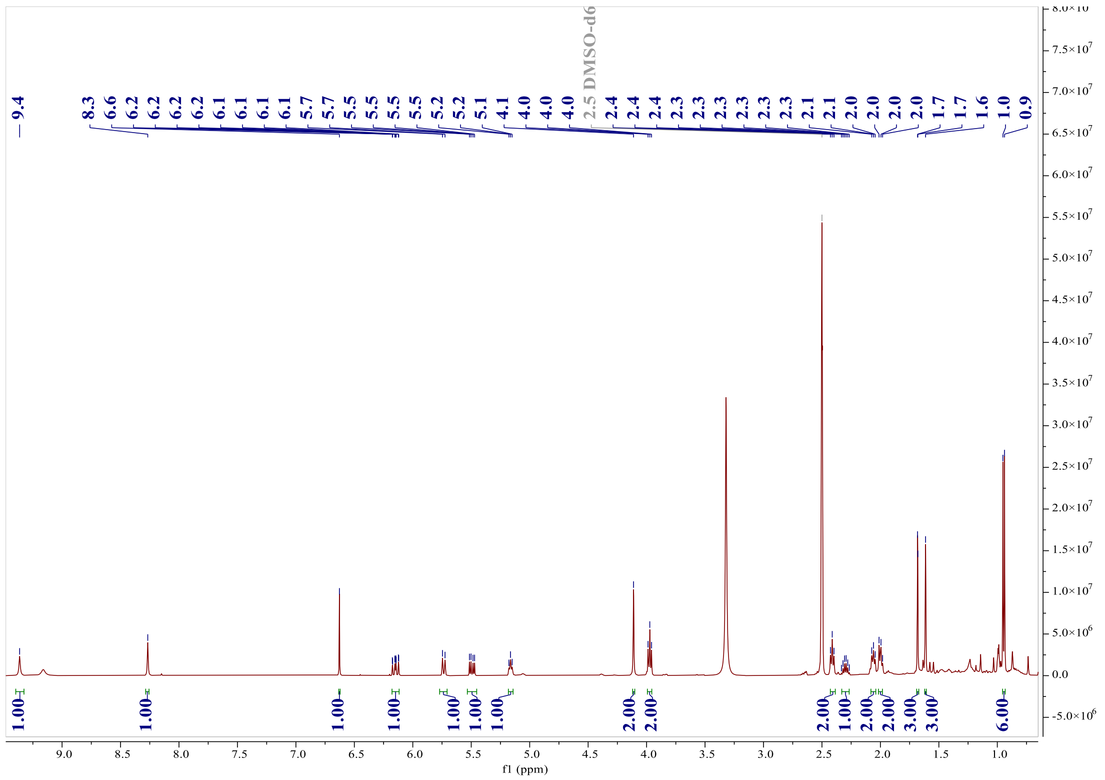

Figure S8. ^{1}H NMR spectrum (500 MHZ) of aspernidine D (**2**) in DMSO-d_6.

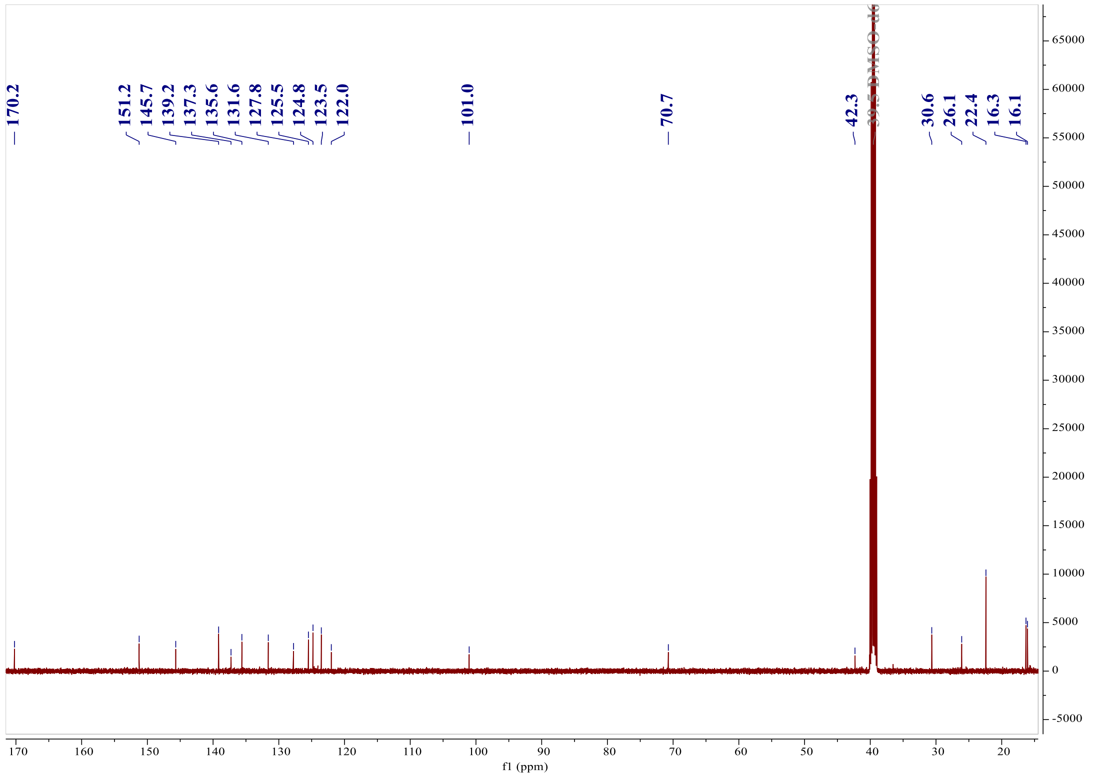

Figure S9. ^{13}C NMR spectrum (125 MHZ) of aspernidine D (**2**) in DMSO-d_6.

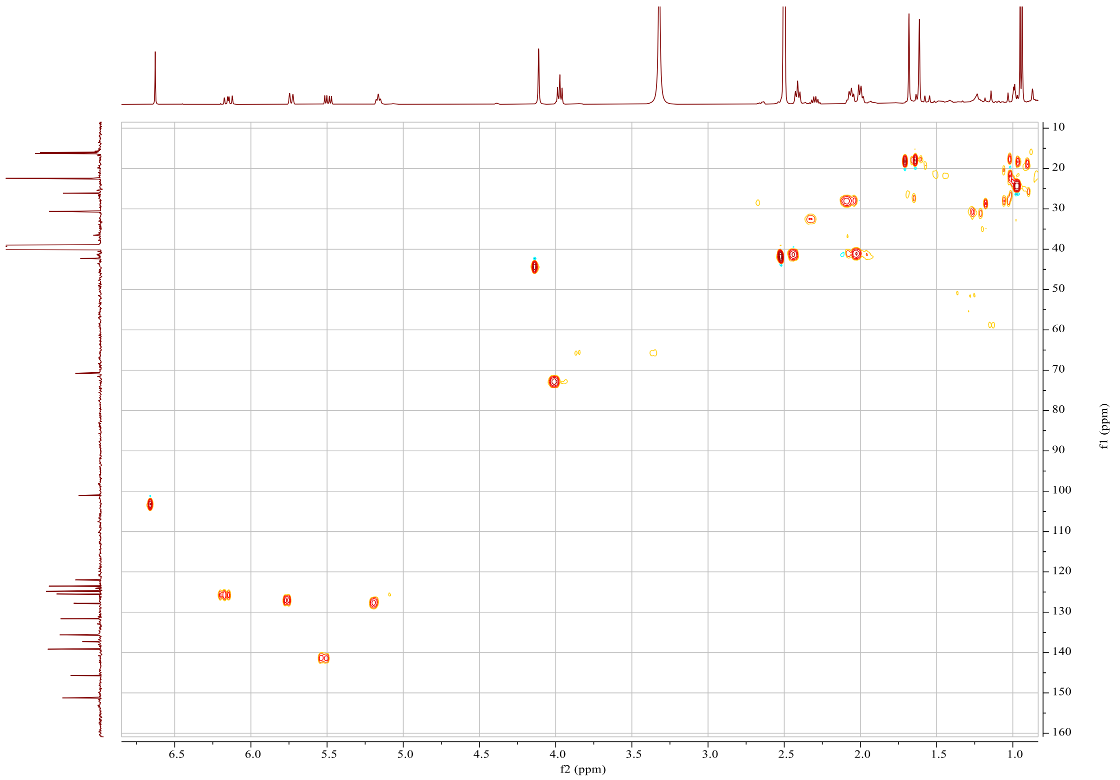

Figure S10. HSQC spectrum (500/125 MHZ) of aspernidine D (**2**) in DMSO-d_6.

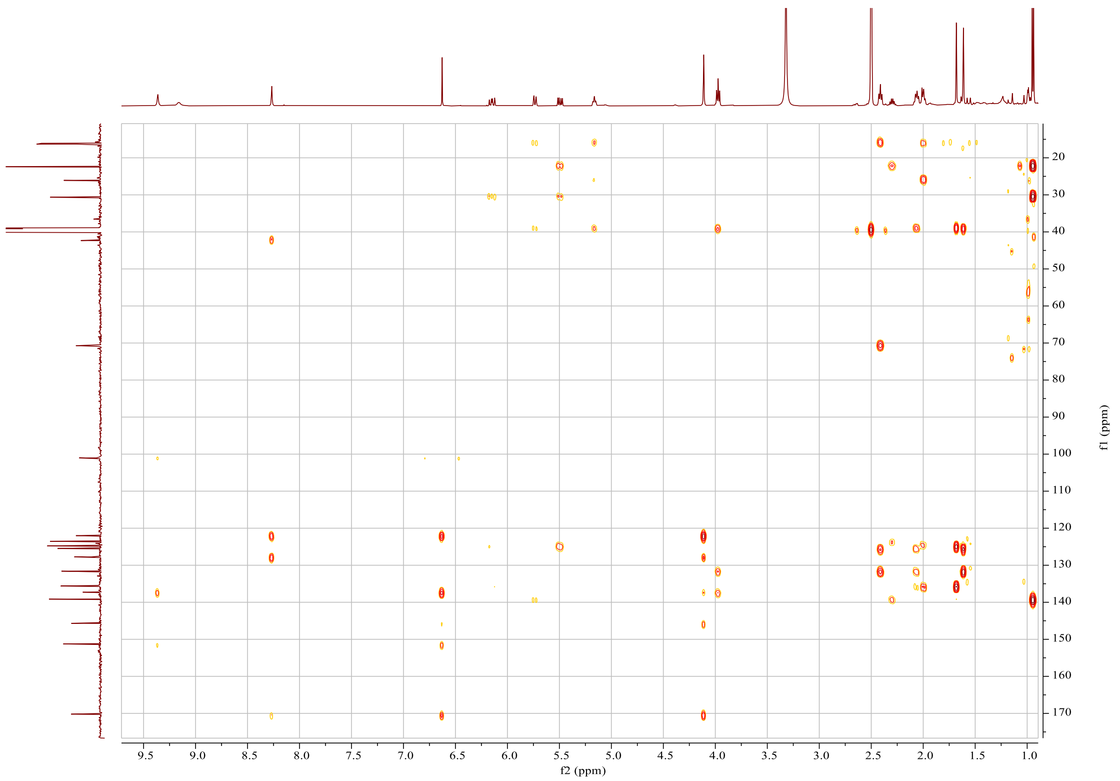

Figure S11. HMBC spectrum (500/125 MHZ) of aspernidine D (**2**) in DMSO-d_6.

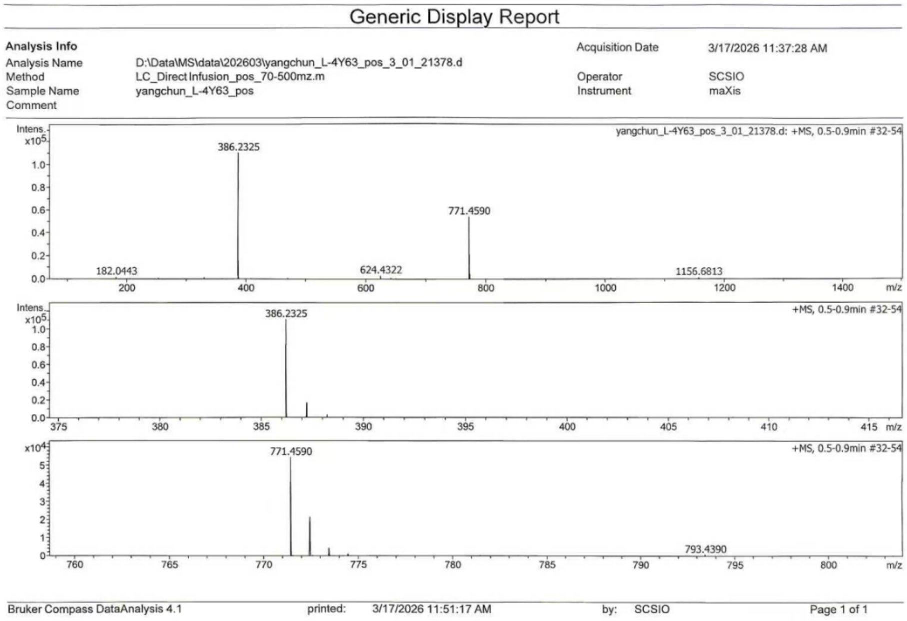

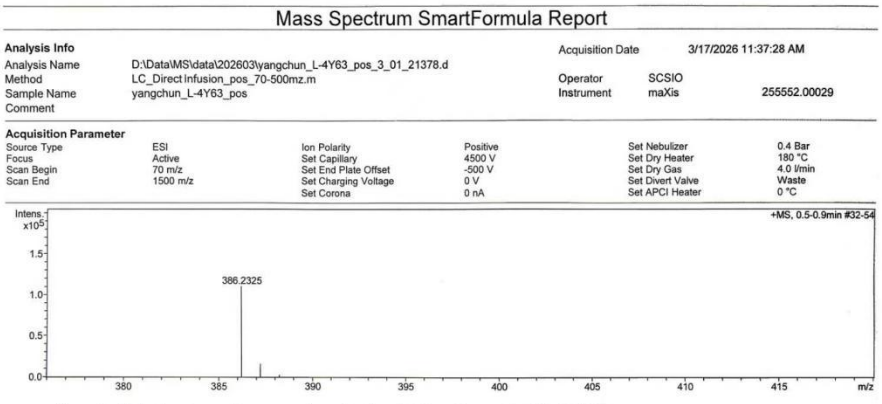

Meas. m/z	#	Ion Formula	Score	m/z	err [ppm]	err [mDa]	mSigma	rdb	e⁻ Conf	N-Rule
386.2325	1	C23H32NO4	100.00	386.2326	-0.3	-0.1	55.6	8.5	even	ok
408.2128	1	C23H31NNaO4	100.00	408.2145	-4.2	-1.7	59.8	8.5	even	ok
771.4590	1	C46H63N2O8	100.00	771.4579	1.5	1.1	60.7	16.5	even	ok
793.4390	1	C46H62N2NaO8	100.00	793.4398	-1.0	-0.8	44.0	16.5	even	ok

Figure S12. HRESIMS spectra of aspernidine D (**2**).

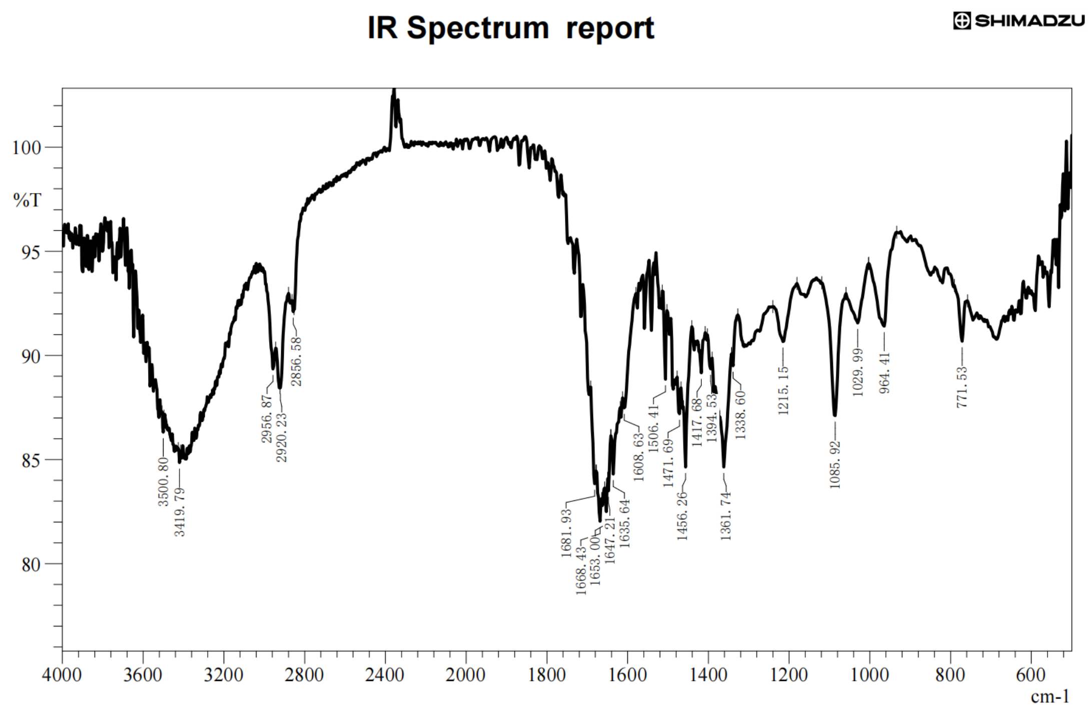

Figure S13. IR spectrum of aspernidine D (**2**).

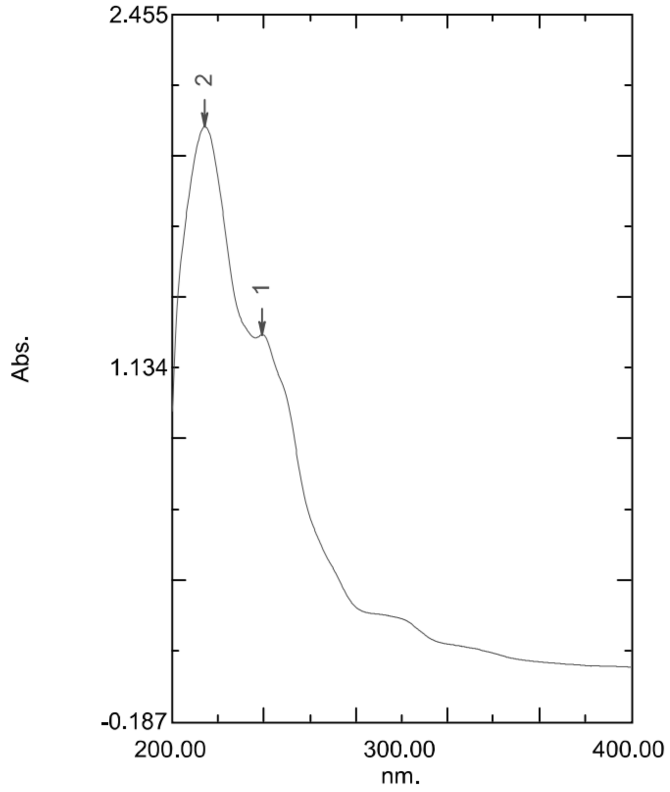

No.	P/V	波长(nm)	Abs.	描述
1	⬆	239. 40	1. 257	
2	⬆	213. 80	2. 037	

Figure S14. UV spectrum of aspernidine D (**2**) in CH_3OH.

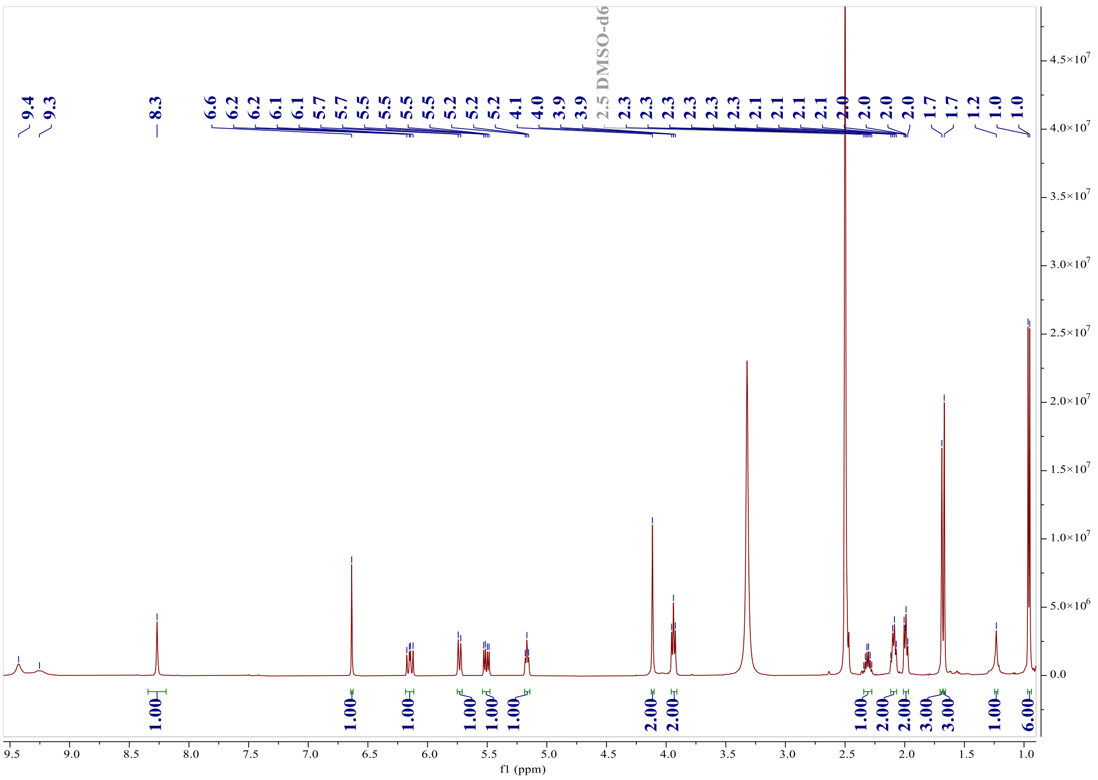

Figure S15. ^{1}H NMR spectrum (500 MHZ) of aspernidine E (**3**) in DMSO-*d*6.

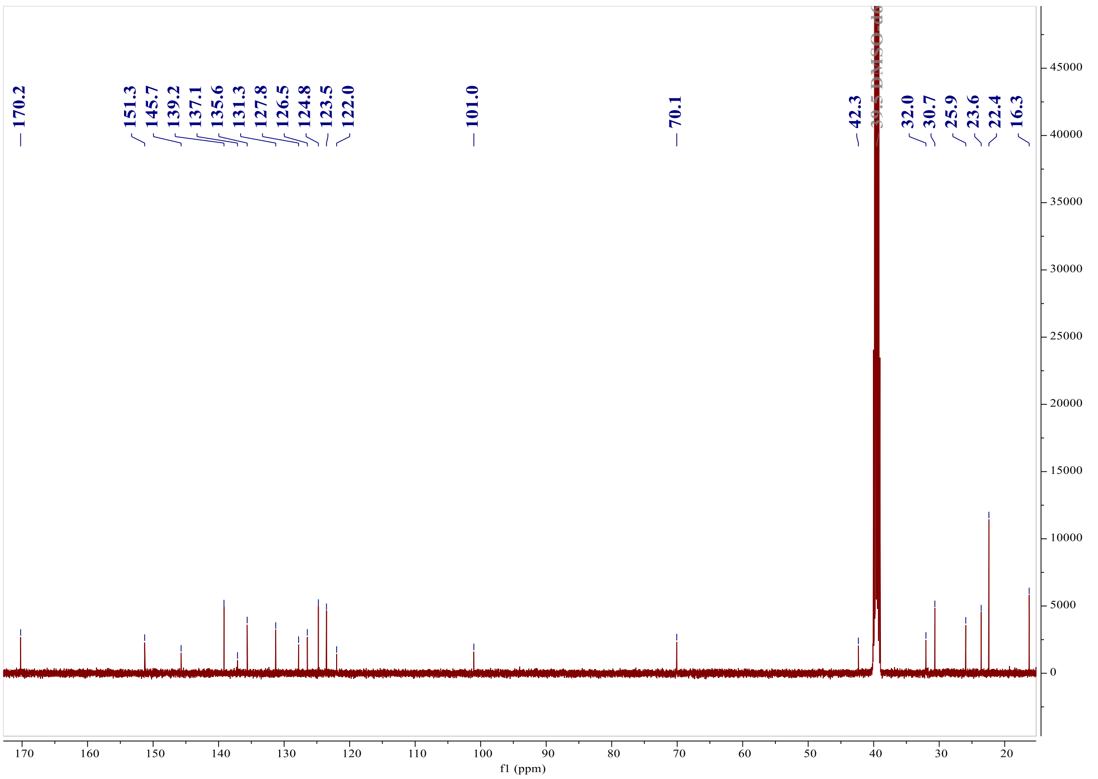

Figure S16. ^{13}C NMR spectrum (125 MHZ) of aspernidine E (**3**) in DMSO-*d*6.

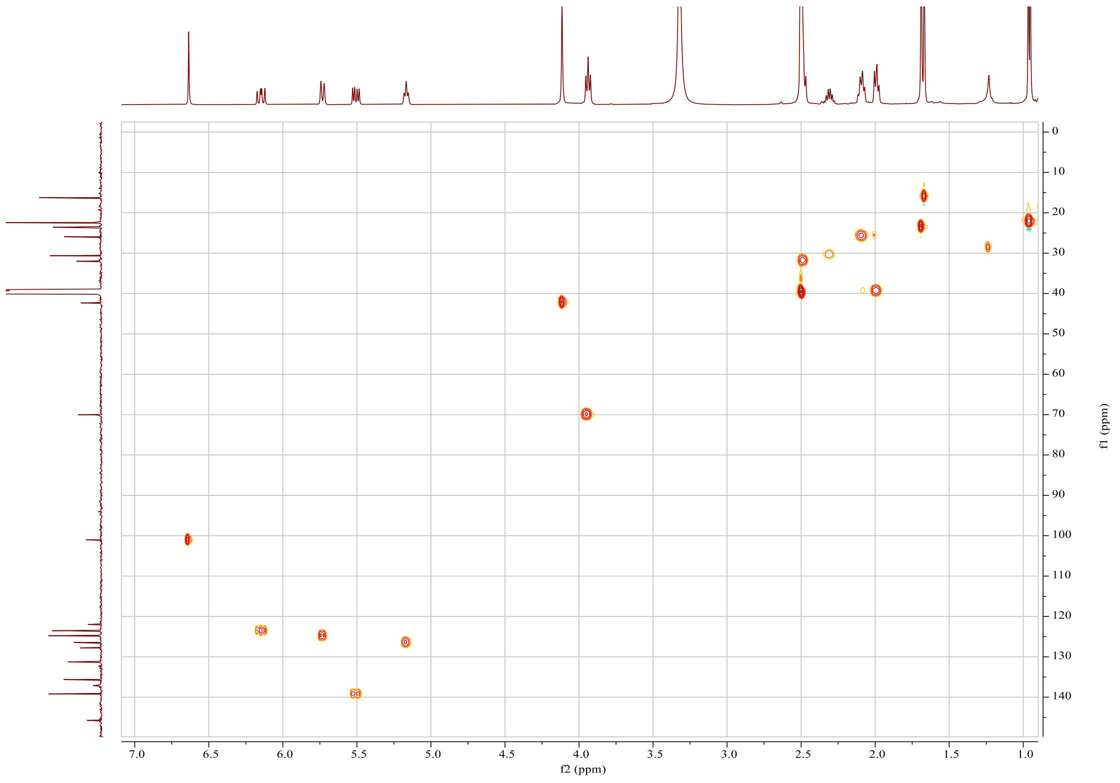

Figure S17. HSQC spectrum (500/125 MHZ) of aspernidine E (**3**) in DMSO-*d*6.

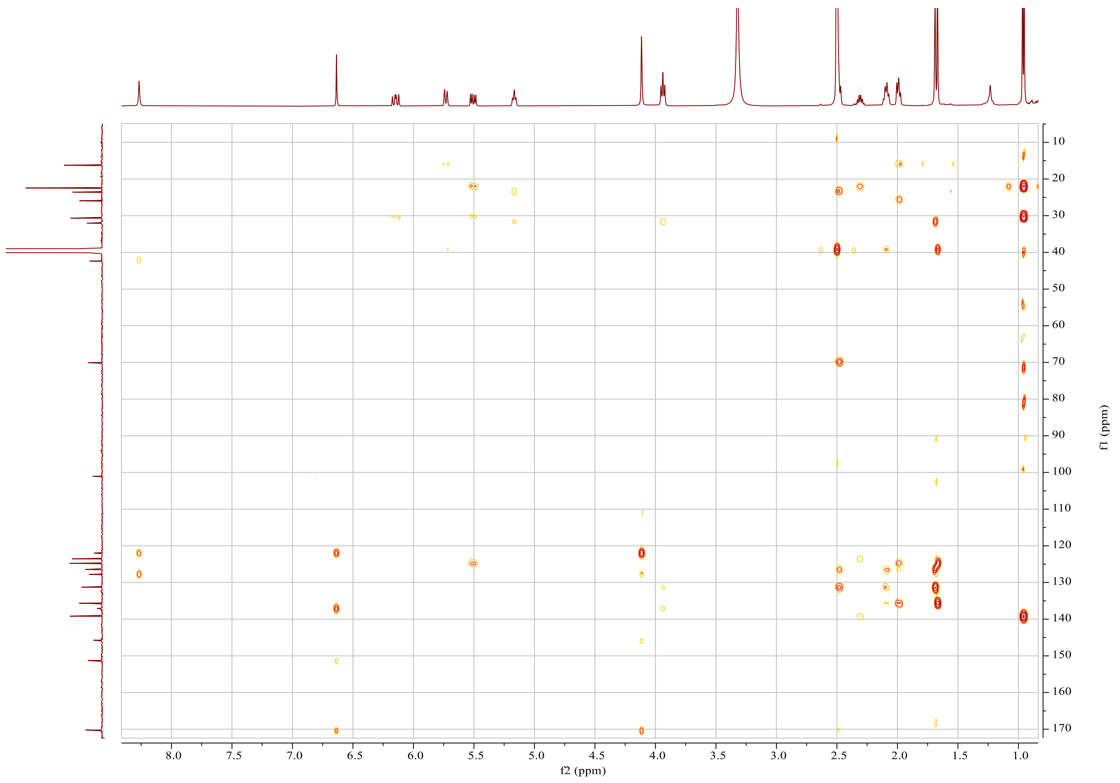

Figure S18. HMBC spectrum (500/125 MHZ) of aspernidine E (**3**) in DMSO-*d*6.

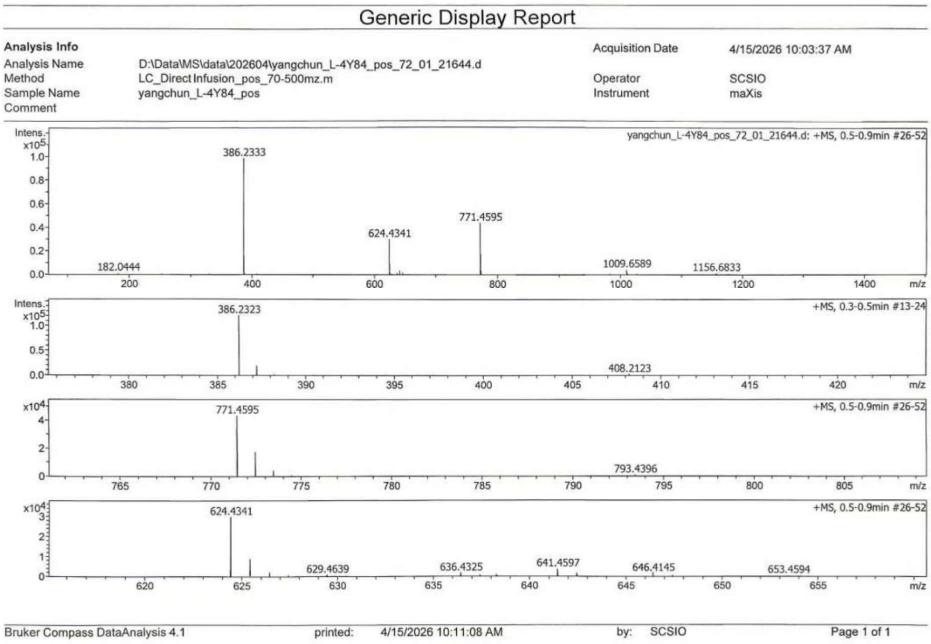

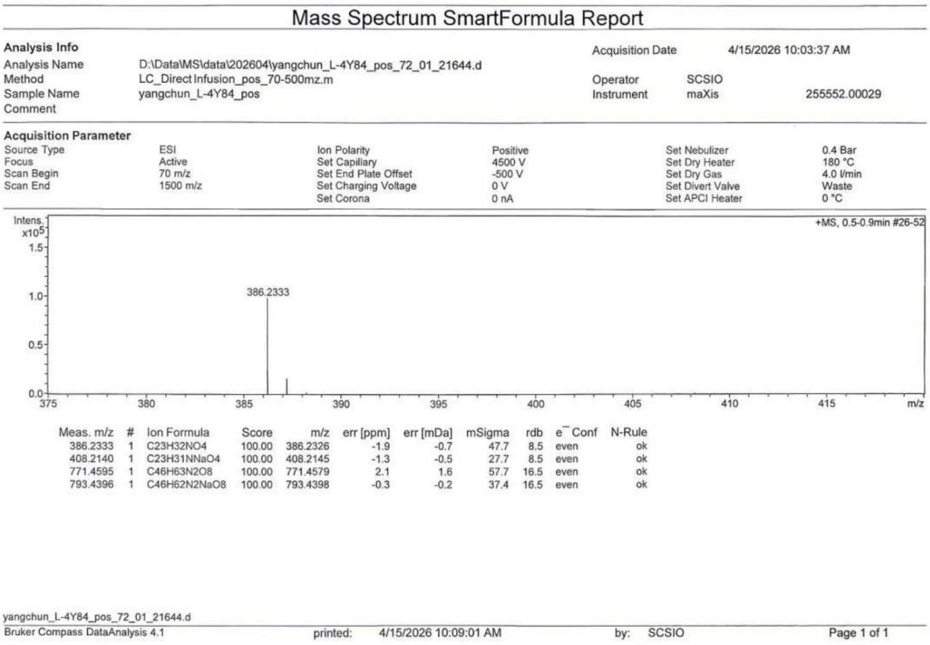

Meas. m/z	#	Ion Formula	Score	m/z	err [ppm]	err [mDa]	mSigma	rdb	e⁻ Conf	N-Rule
386.2333	1	C23H32NO4	100.00	386.2326	-1.9	-0.7	47.7	8.5	even	ok
408.2140	1	C23H31NNaO4	100.00	408.2145	-1.3	-0.5	27.7	8.5	even	ok
771.4595	1	C46H63N2O8	100.00	771.4579	2.1	1.6	57.7	16.5	even	ok
793.4396	1	C46H62N2NaO8	100.00	793.4398	-0.3	-0.2	37.4	16.5	even	ok

Figure S19. HRESIMS spectra of aspernidine E (**3**).

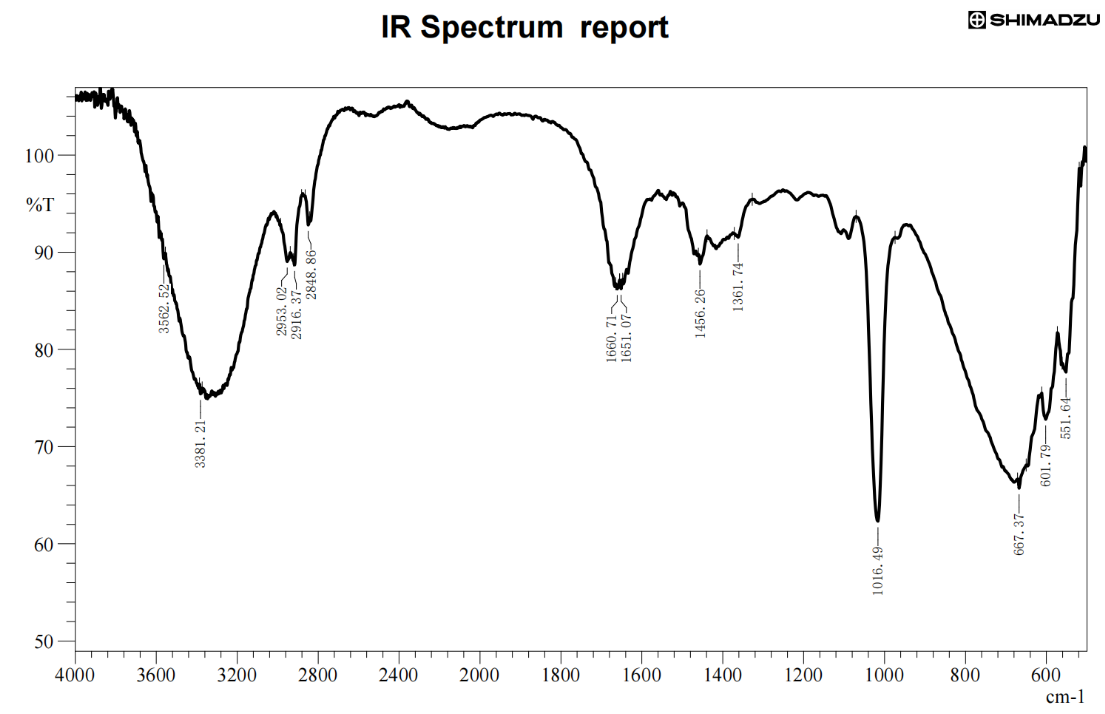

Figure S20. IR spectrum of aspernidine E (**3**).

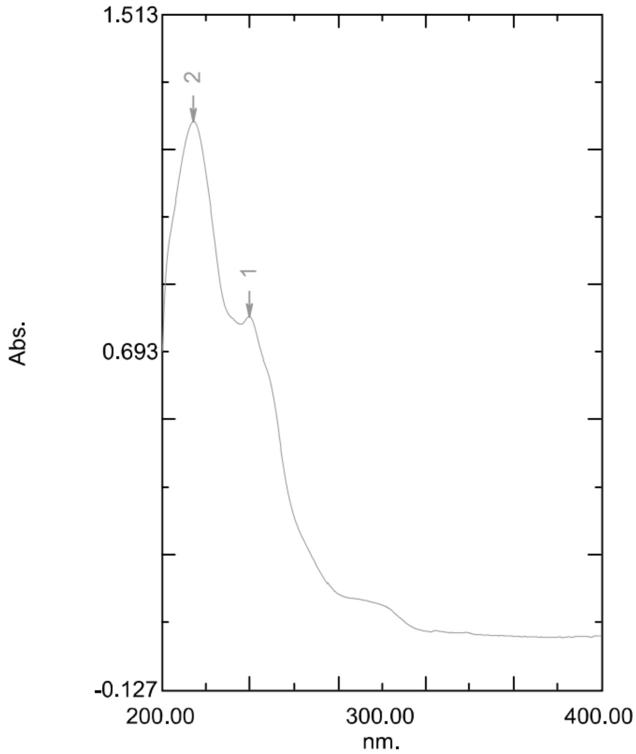

No.	P/V	波长(nm)	Abs.	描述
1		239.60	0.778	
2		214.20	1.254	

Figure S21. UV spectrum of aspernidine E (**3**) in CH_3OH.

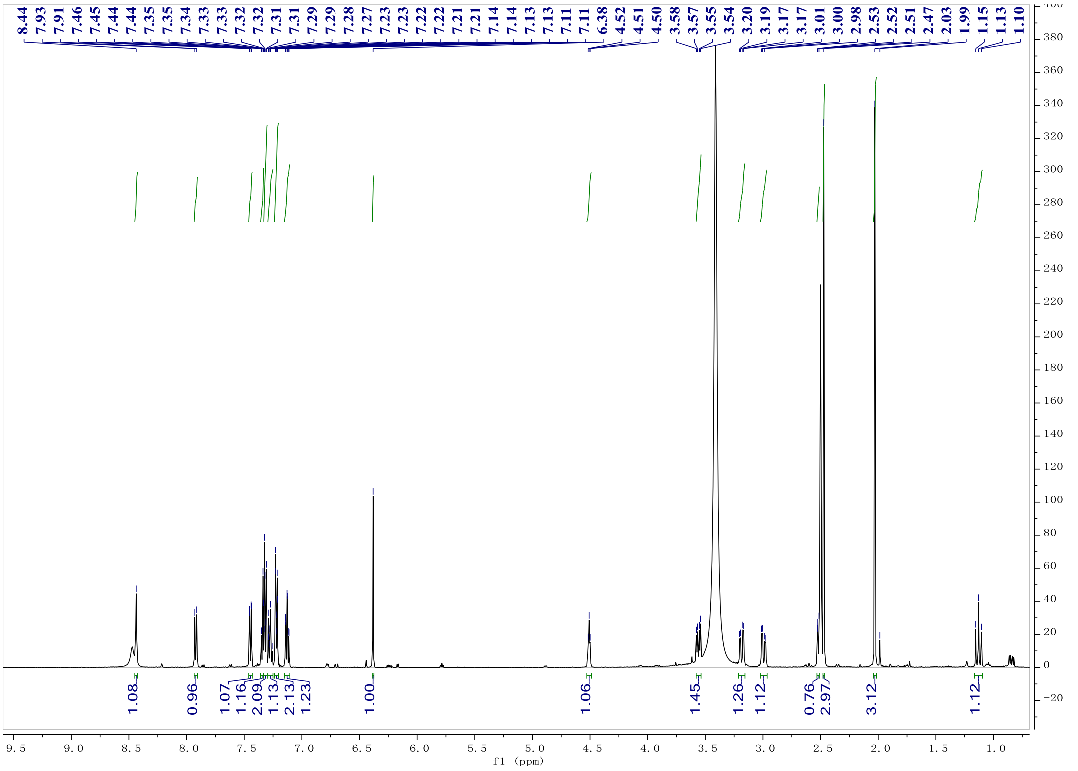

Figure S22. ^{1}H NMR spectrum (500 MHZ) of 11 (**4**) in DMSO-d_6.

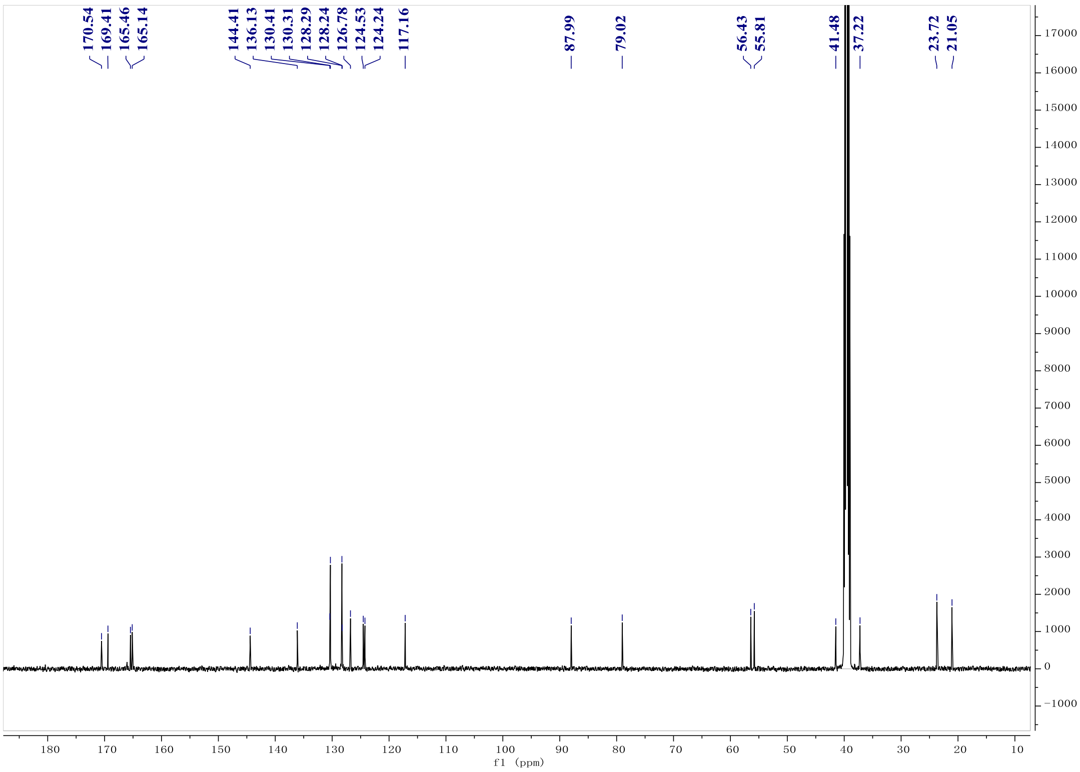

Figure S23. ^{13}C NMR spectrum (125 MHZ) of protubonine C (**4**) in DMSO-d_6.

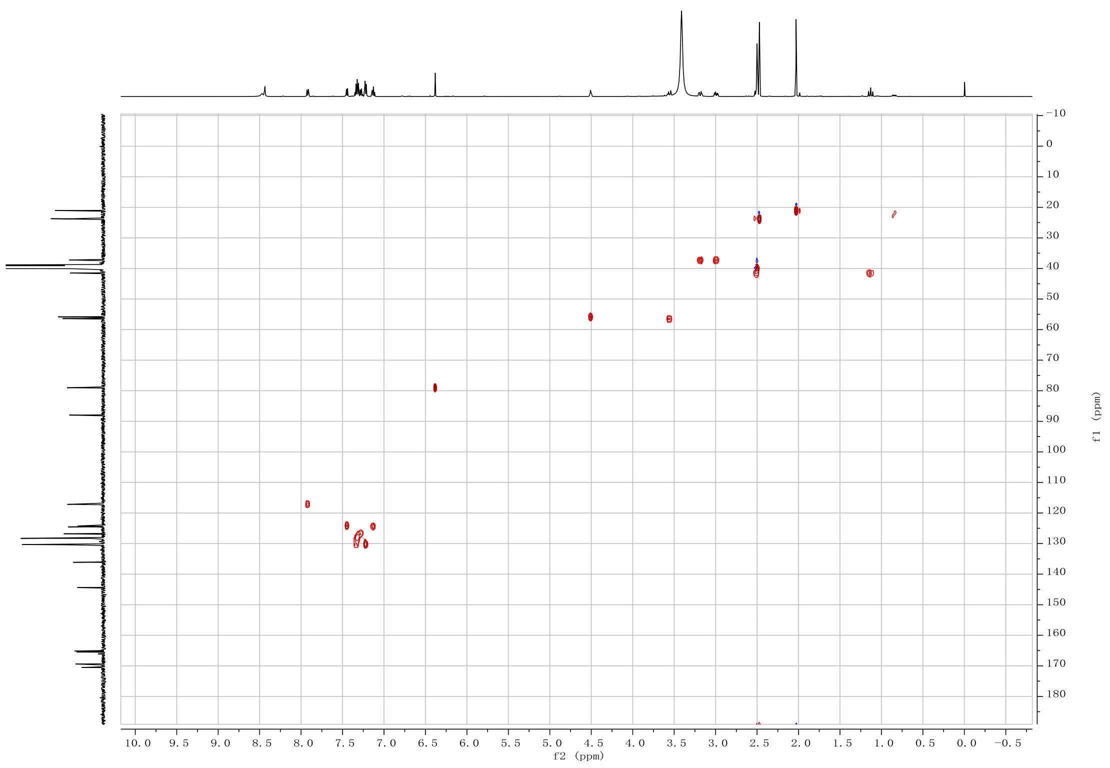

Figure S24. HSQC spectrum (500/125 MHZ) of protubonine C (**4**) in DMSO-d_6.

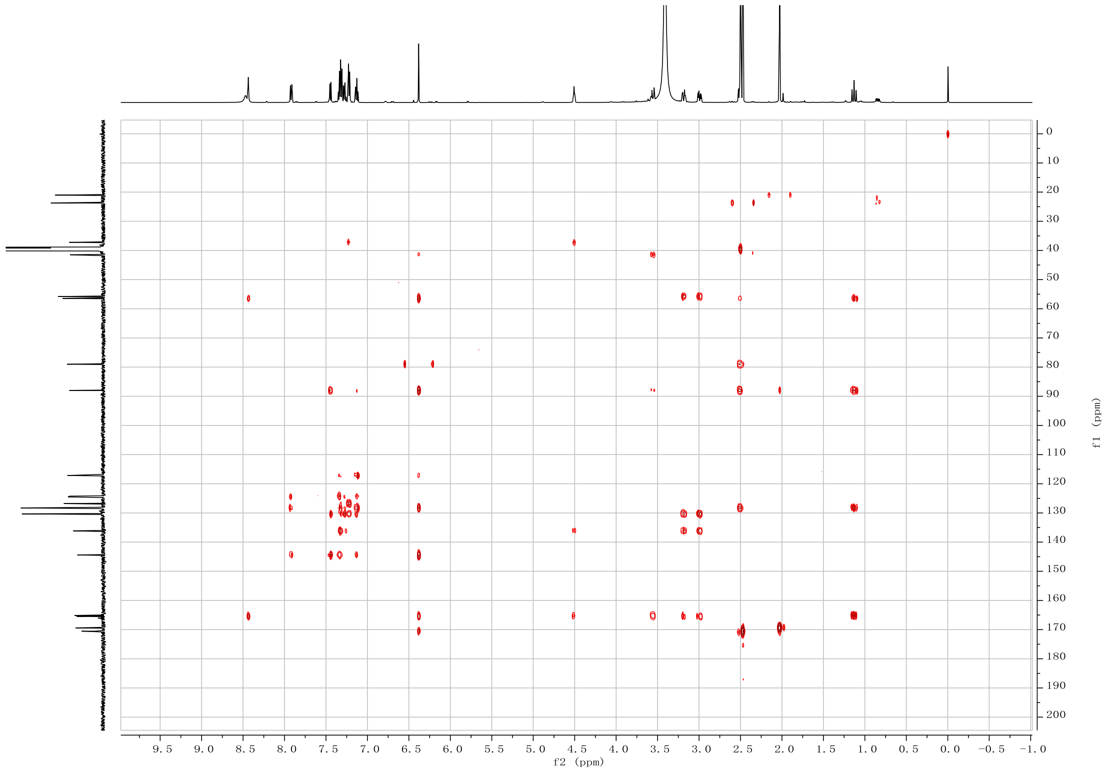

Figure S25. HMBC spectrum (500/125 MHZ) of protubonine C (**4**) in DMSO-d_6.

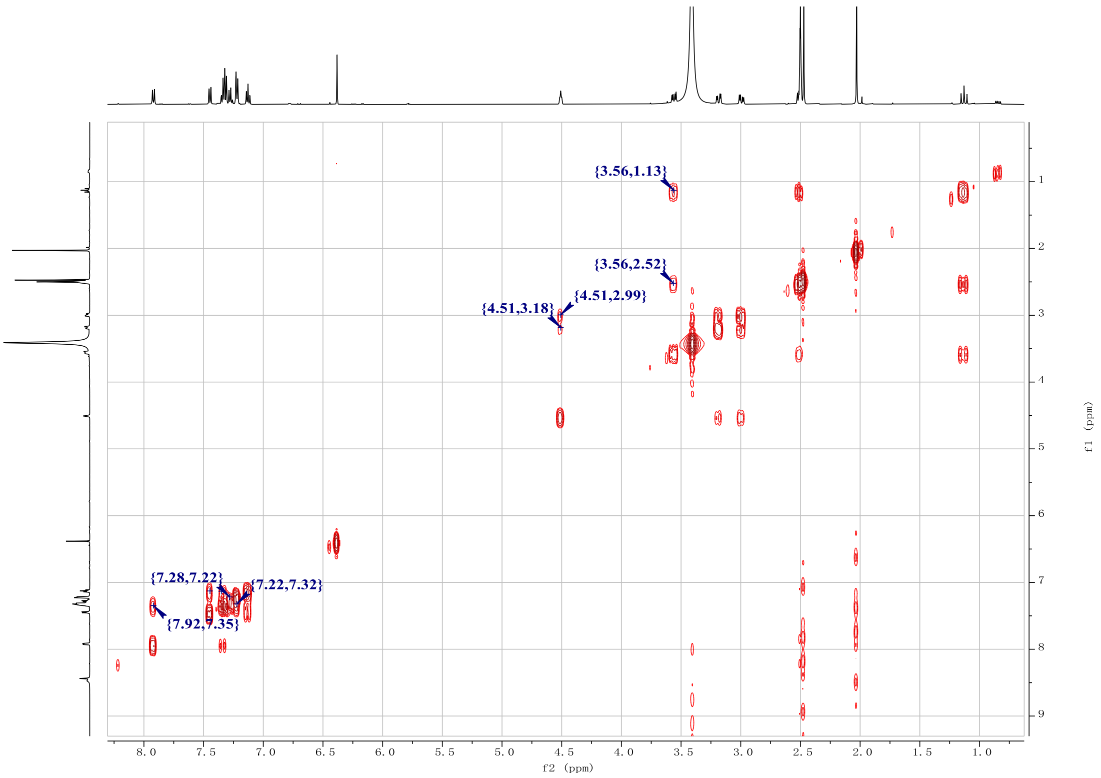

Figure S26. ^1H–^1H COSY spectrum (500 MHZ) of protubonine C (**4**) in DMSO-d_6.

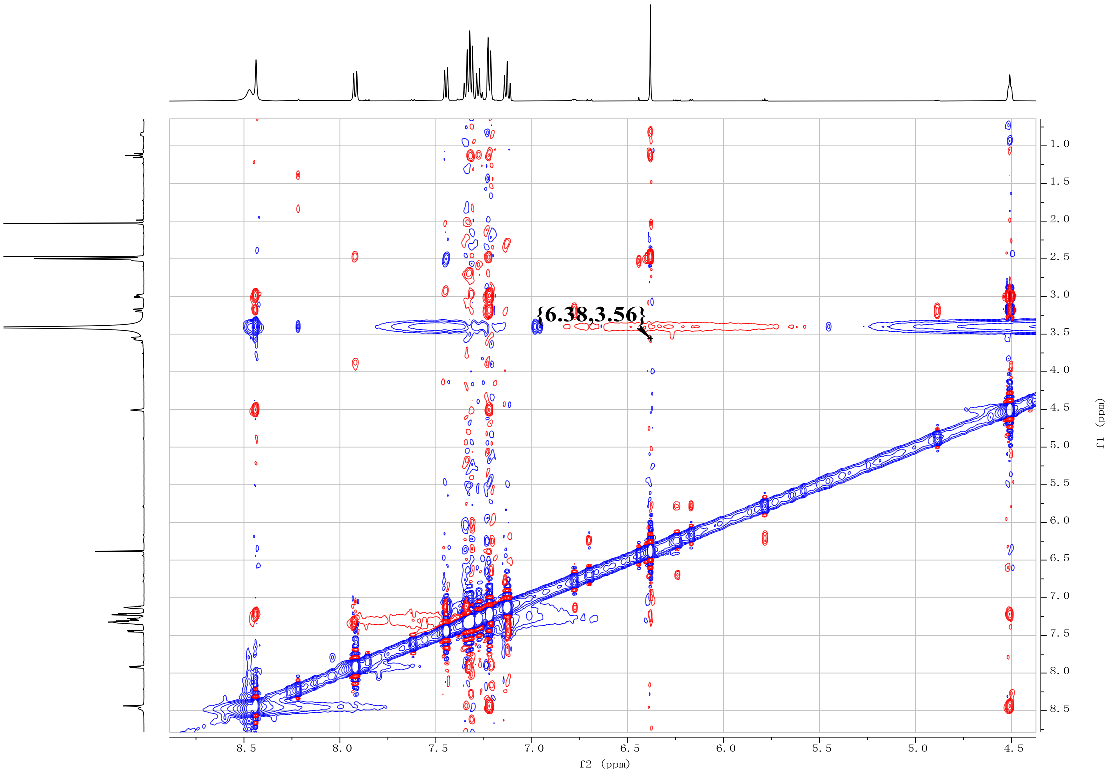

Figure S27. NOESY spectrum (700 MHZ) of protubonine C (**4**) in DMSO-d_6.

Generic Display Report

Analysis Info		Acquisition Date	9/10/2024 9:34:59 AM
Analysis Name	D:\Data\MS\data\202409\chenyi_L-4D79_pos_3_01_18064.d		
Method	LC_Direct Infusion_pos_70-500mz.m	Operator	SCSIO
Sample Name	chenyi_L-4D79_pos	Instrument	maXis
Comment			

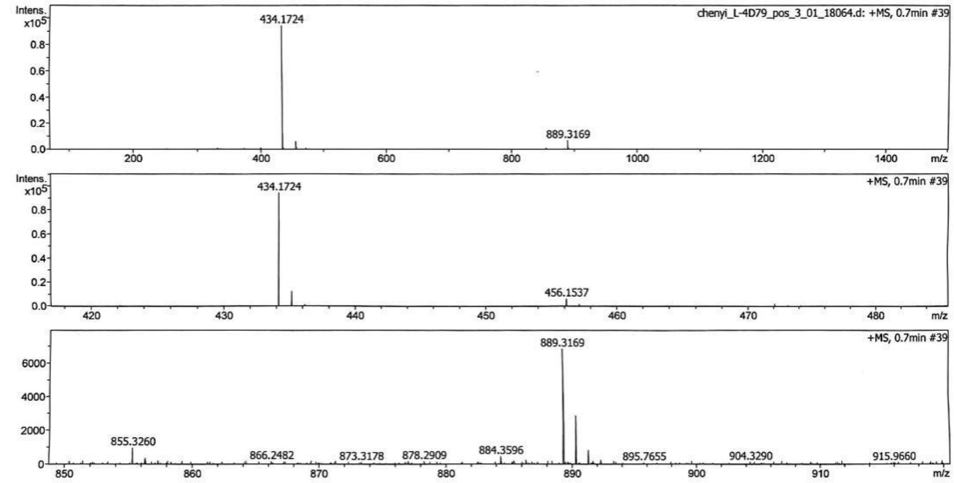

Bruker Compass DataAnalysis 4.1 printed: 9/10/2024 9:40:44 AM by: SCSIO Page 1 of 1

Mass Spectrum SmartFormula Report

Analysis Info		Acquisition Date	9/10/2024 9:34:59 AM	
Analysis Name	D:\Data\MS\data\202409\chenyi_L-4D79_pos_3_01_18064.d			
Method	LC_Direct Infusion_pos_70-500mz.m	Operator	SCSIO	
Sample Name	chenyi_L-4D79_pos	Instrument	maXis	255552.00029
Comment				

Acquisition Parameter

Source Type	ESI	Ion Polarity	Positive	Set Nebulizer	0.4 Bar
Focus	Active	Set Capillary	4500 V	Set Dry Heater	180 °C
Scan Begin	70 m/z	Set End Plate Offset	-500 V	Set Dry Gas	4.0 l/min
Scan End	1500 m/z	Set Charging Voltage	0 V	Set Divert Valve	Waste
		Set Corona	0 nA	Set APCI Heater	0 °C

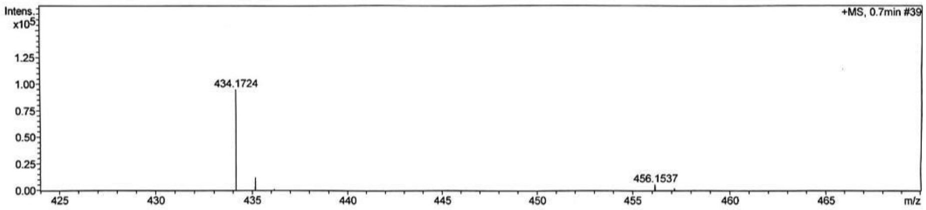

Meas. m/z	#	Ion Formula	Score	m/z	err [ppm]	err [mDa]	mSigma	rdb	e⁻ Conf	N-Rule
434.1724	1	C24H24N3O5	80.41	434.1710	-3.1	-1.3	74.4	14.5	even	ok
456.1537	1	C24H23N3NaO5	100.00	456.1530	-1.5	-0.7	10.2	14.5	even	ok
889.3169	1	C48H46N6NaO10	100.00	889.3168	0.2	0.2	60.8	28.5	even	ok

chenyi_L-4D79_pos_3_01_18064.d

Bruker Compass DataAnalysis 4.1 printed: 9/10/2024 9:40:10 AM by: SCSIO Page 1 of 1

Figure S28. HRESIMS spectra of protubonine C (**4**).

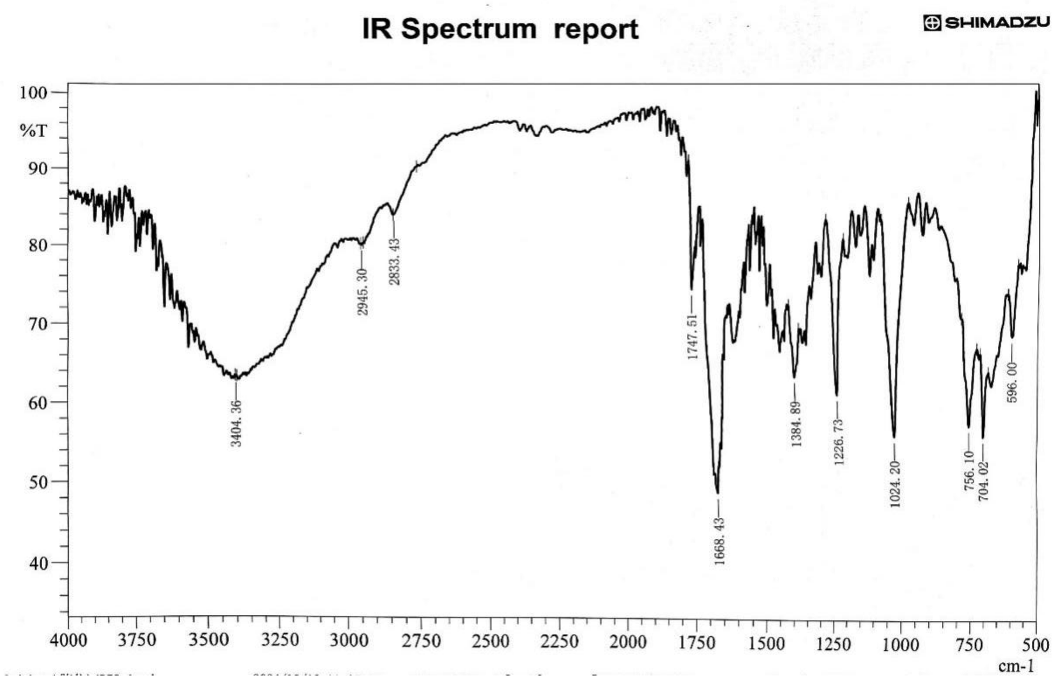

Figure S29. IR spectrum of protubonine C (**4**).

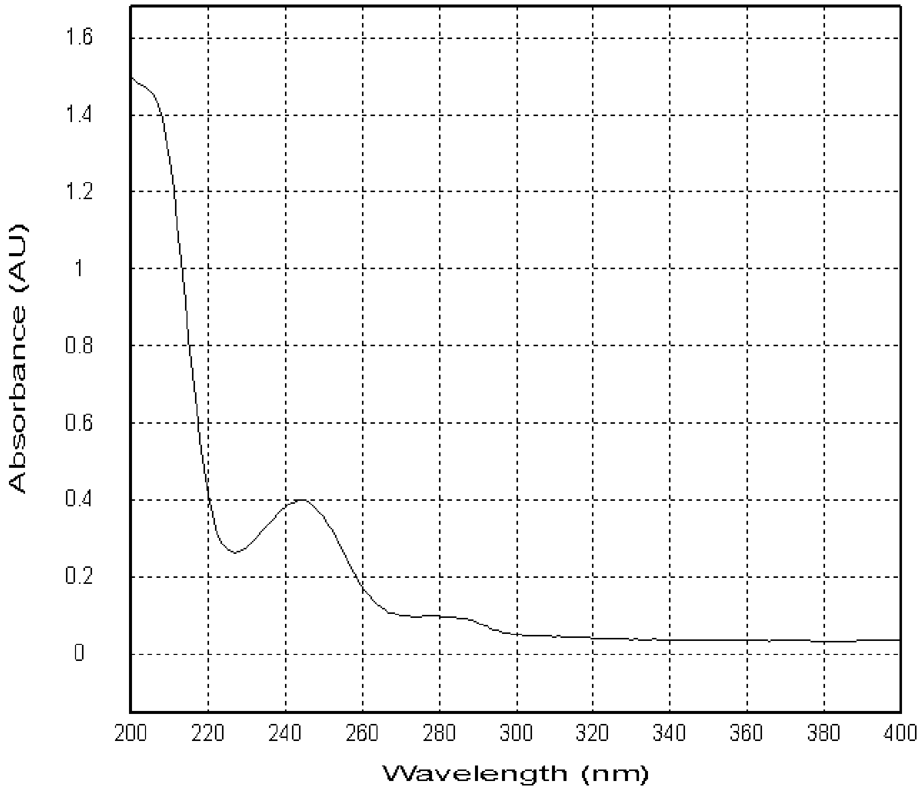

Figure S30. UV spectrum of protubonine C (**4**) in CH_3OH.

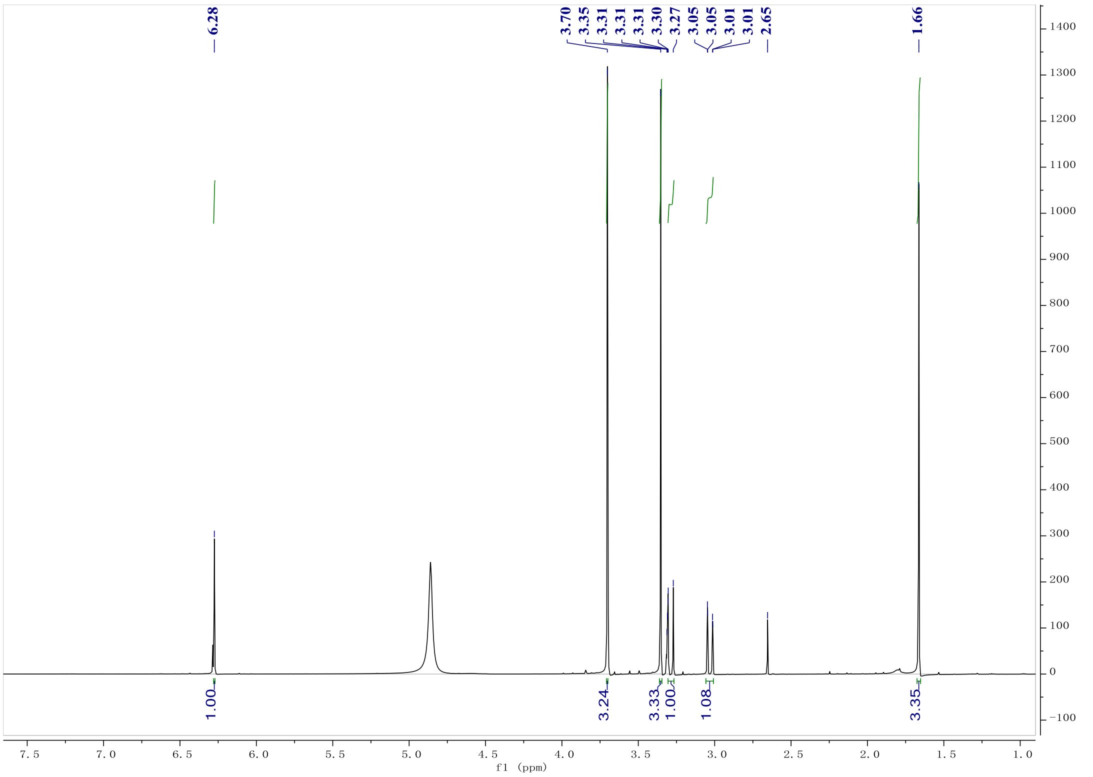

Figure S31. ^{1}H NMR spectrum (500 MHZ) of (±)-asperisocoumarin J (**5**) in CD_3OD.

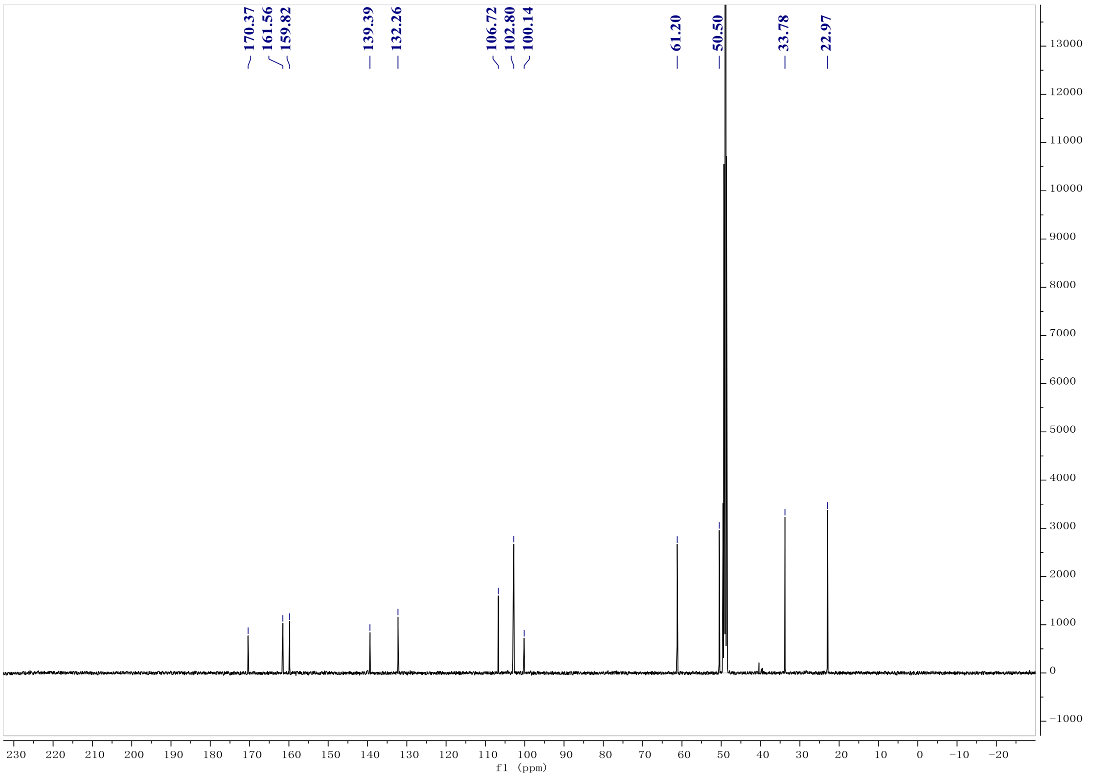

Figure S32. ^{13}C NMR spectrum (125 MHZ) of (±)-asperisocoumarin J (**5**) in CD_3OD.

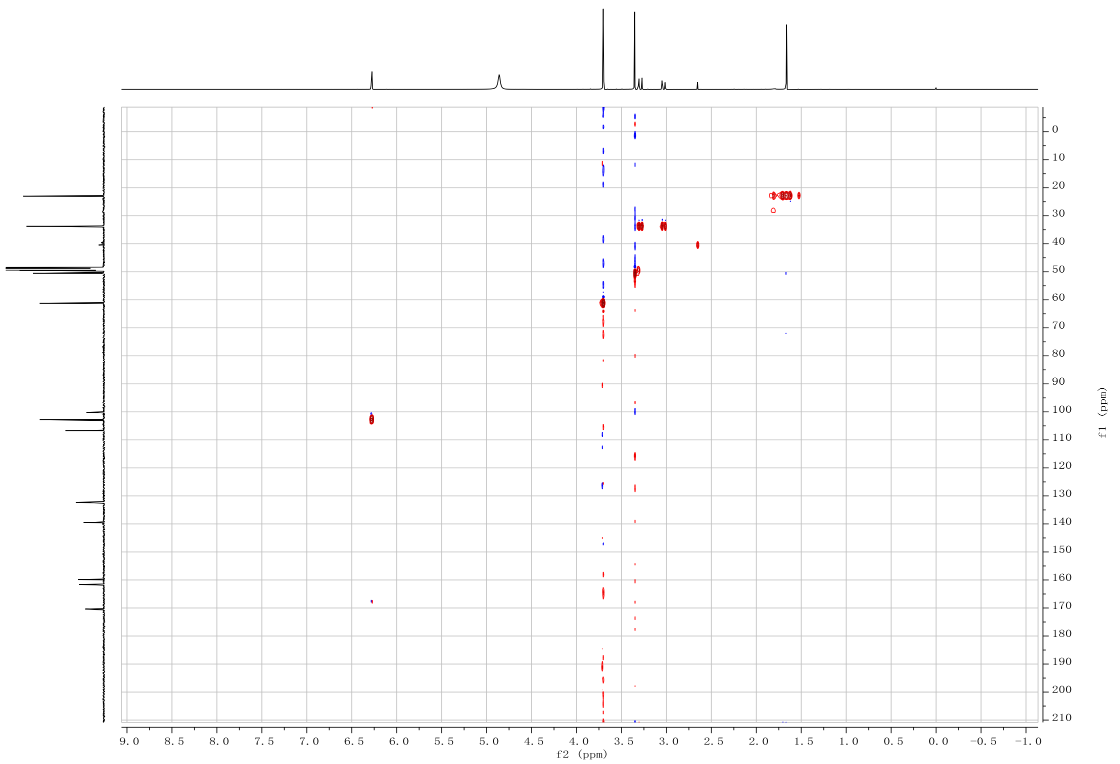

Figure S33. HSQC spectrum (500/125 MHZ) of (±)-asperisocoumarin J (**5**) in CD_3OD.

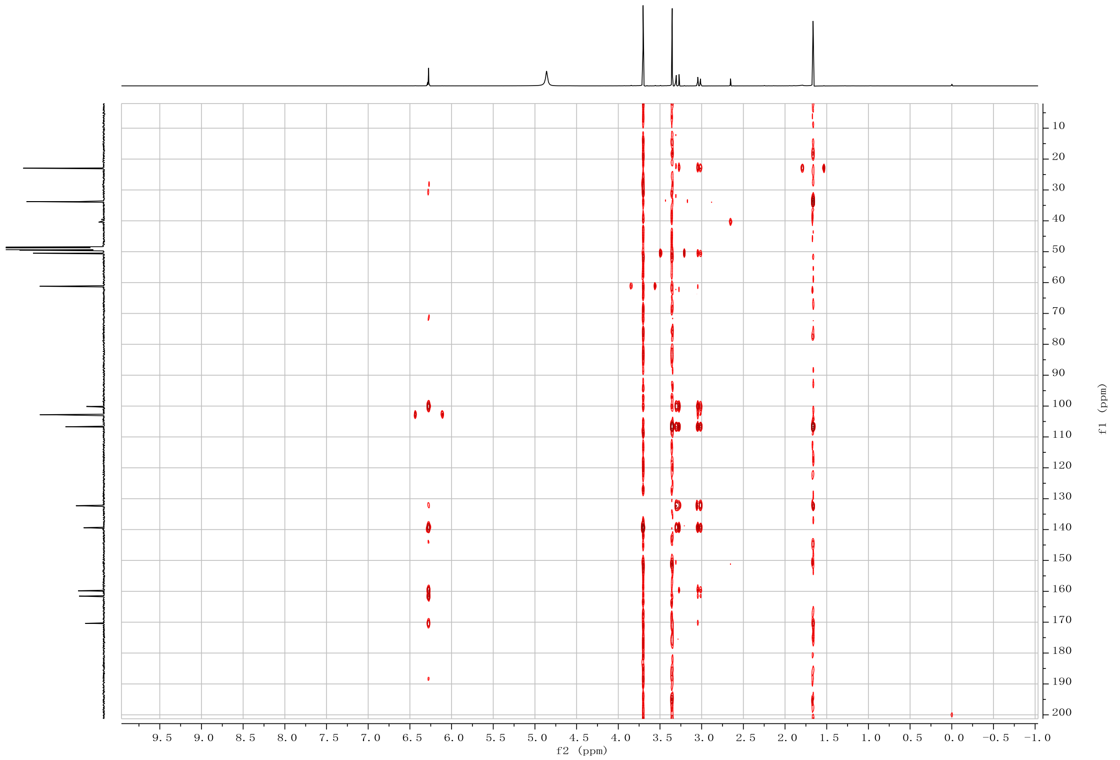

Figure S34. HMBC spectrum (500/125 MHZ) of (±)-asperisocoumarin J (**5**) in CD_3OD.

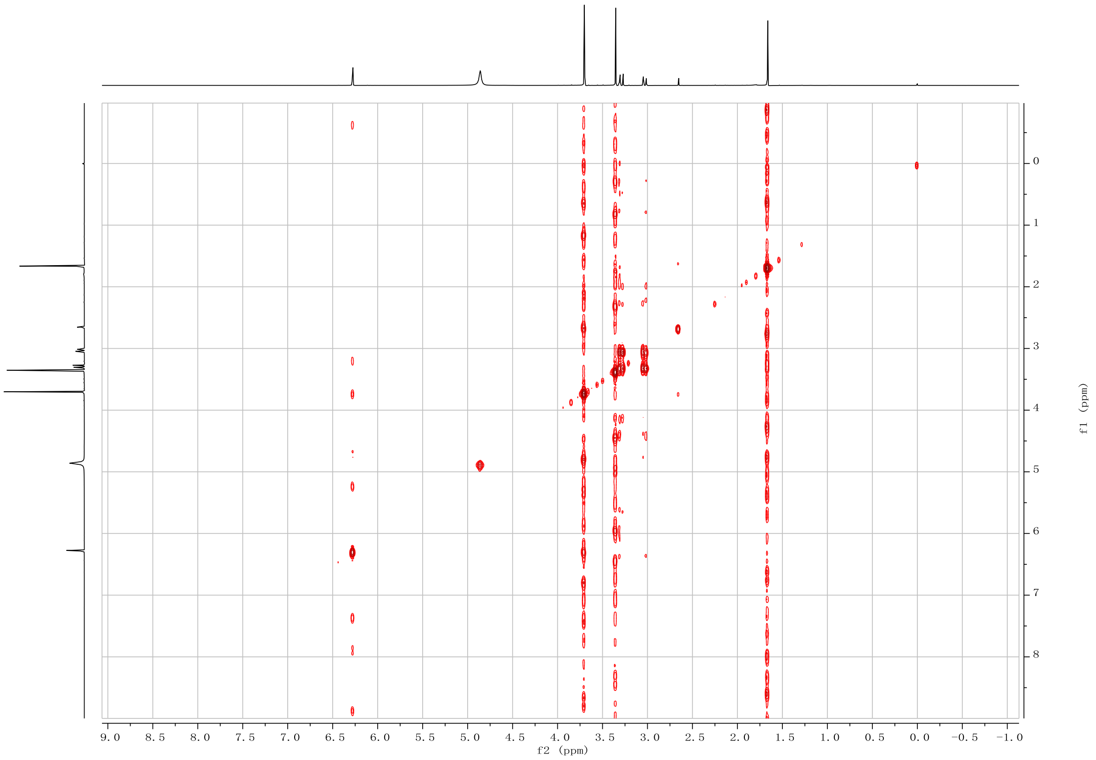

Figure S35. ^1H–^1H COSY spectrum (500 MHZ) of (±)-asperisocoumarin J (**5**) in CD_3OD.

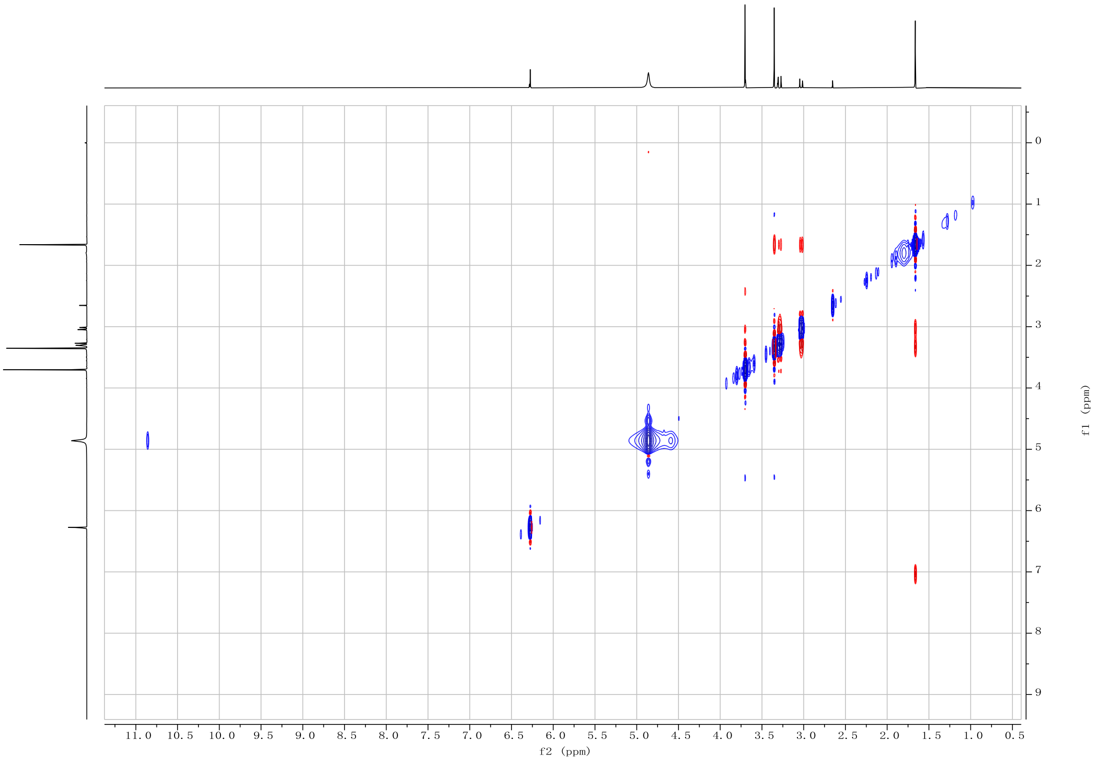

Figure S36. NOESY spectrum (700 MHZ) of (±)-asperisocoumarin J (**5**) in CD_3OD.

Generic Display Report

**Analysis Info**		Acquisition Date	7/4/2024 5:31:41 PM
Analysis Name	D:\Data\MS\data\202407\chenyi_4D32_neg_23_01_17613.d		
Method	LC_Direct Infusion_neg_70-500mz.m	Operator	SCSIO
Sample Name	chenyi_4D32_neg	Instrument	maXis
Comment			

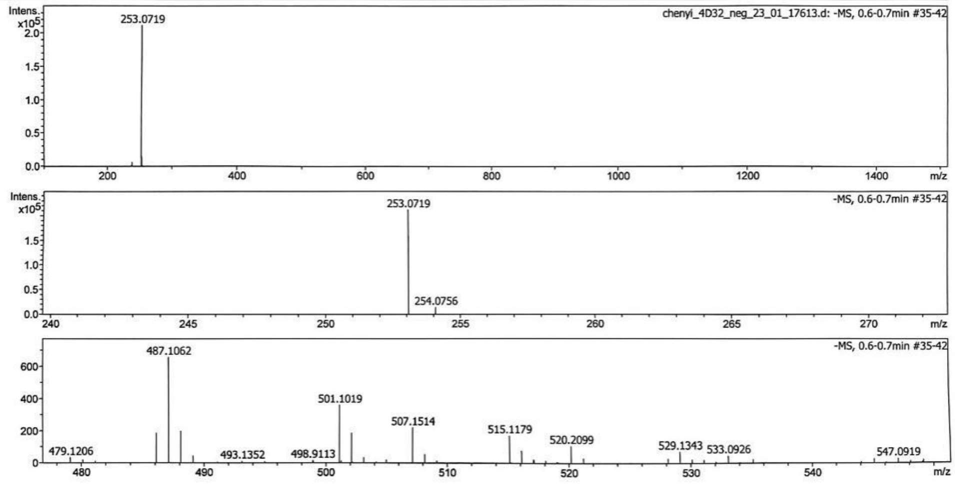

Bruker Compass DataAnalysis 4.1 printed: 7/4/2024 5:37:16 PM by: SCSIO Page 1 of 1

Mass Spectrum SmartFormula Report

**Analysis Info**		Acquisition Date	7/4/2024 5:31:41 PM	
Analysis Name	D:\Data\MS\data\202407\chenyi_4D32_neg_23_01_17613.d			
Method	LC_Direct Infusion_neg_70-500mz.m	Operator	SCSIO	
Sample Name	chenyi_4D32_neg	Instrument	maXis	255552.00029
Comment				

Acquisition Parameter

Source Type	ESI	Ion Polarity	Negative	Set Nebulizer	0.4 Bar
Focus	Active	Set Capillary	4200 V	Set Dry Heater	180 °C
Scan Begin	100 m/z	Set End Plate Offset	-500 V	Set Dry Gas	4.0 l/min
Scan End	1500 m/z	Set Charging Voltage	0 V	Set Divert Valve	Waste
		Set Corona	0 nA	Set APCI Heater	0 °C

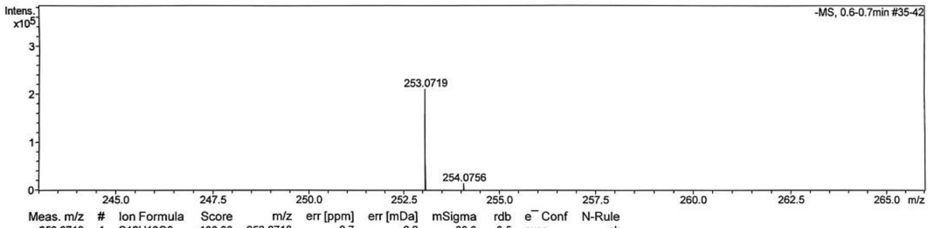

Meas. m/z	#	Ion Formula	Score	m/z	err [ppm]	err [mDa]	mSigma	rdb	e⁻ Conf	N-Rule
253.0719	1	C12H13O6	100.00	253.0718	-0.7	-0.2	38.6	6.5	even	ok
507.1514	1	C24H27O12	100.00	507.1508	1.2	0.6	13.3	11.5	even	ok

chenyi_4D32_neg_23_01_17613.d
Bruker Compass DataAnalysis 4.1 printed: 7/4/2024 5:36:48 PM by: SCSIO Page 1 of 1

Figure S37. HRESIMS spectra of (±)-asperisocoumarin J (**5**).

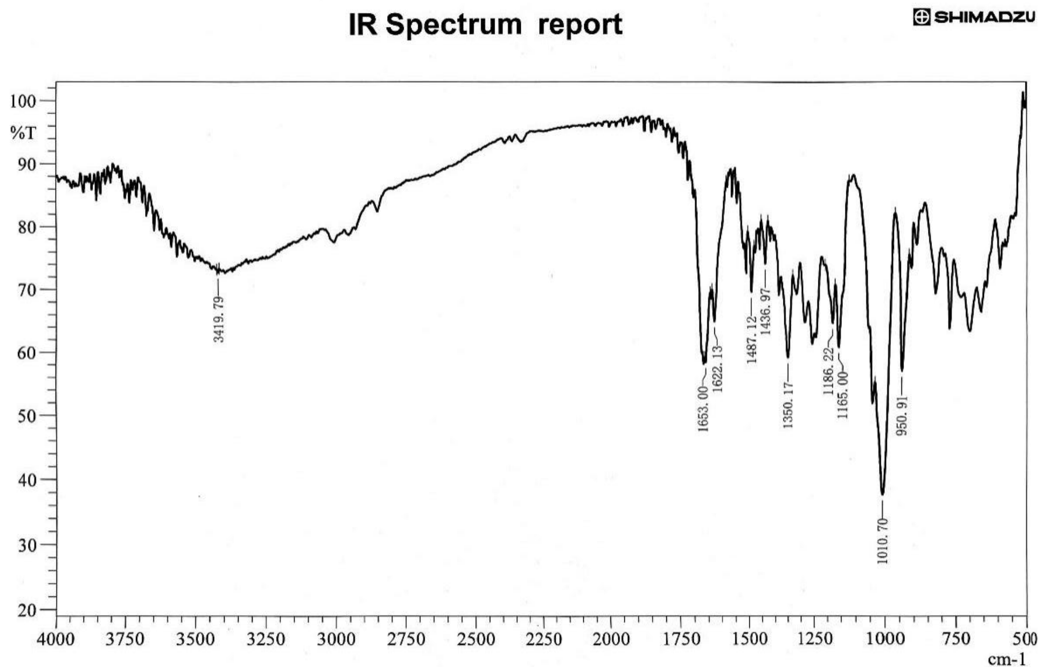

Figure S38. IR spectrum of (±)-asperisocoumarin J (**5**).

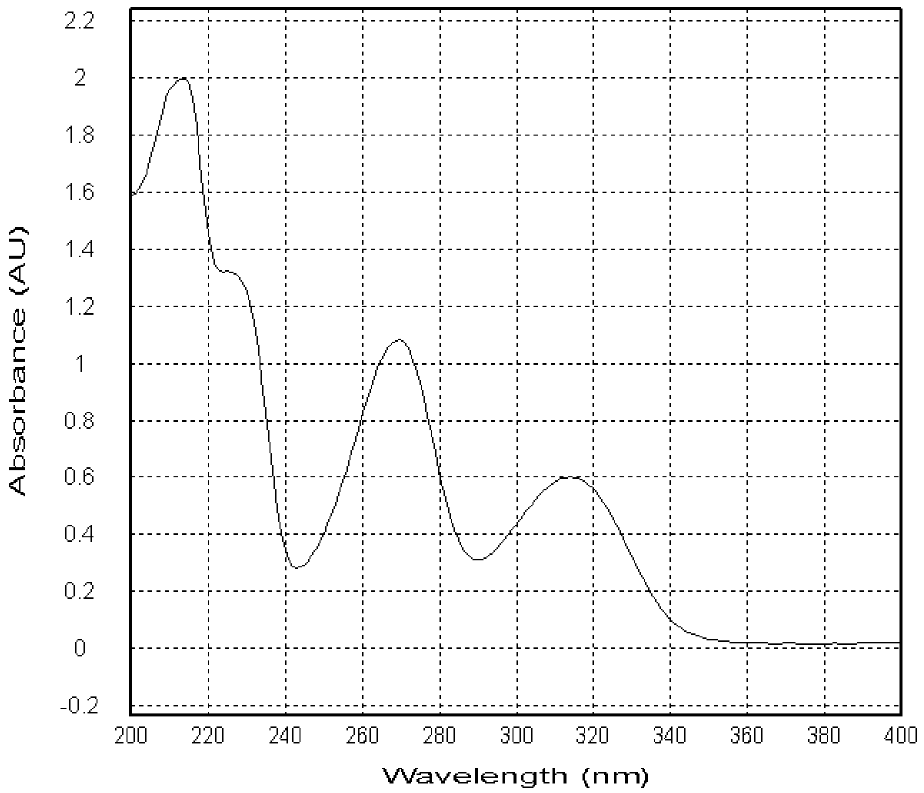

Figure S39. UV spectrum of (±)-asperisocoumarin J (**5**) in CH_3OH.

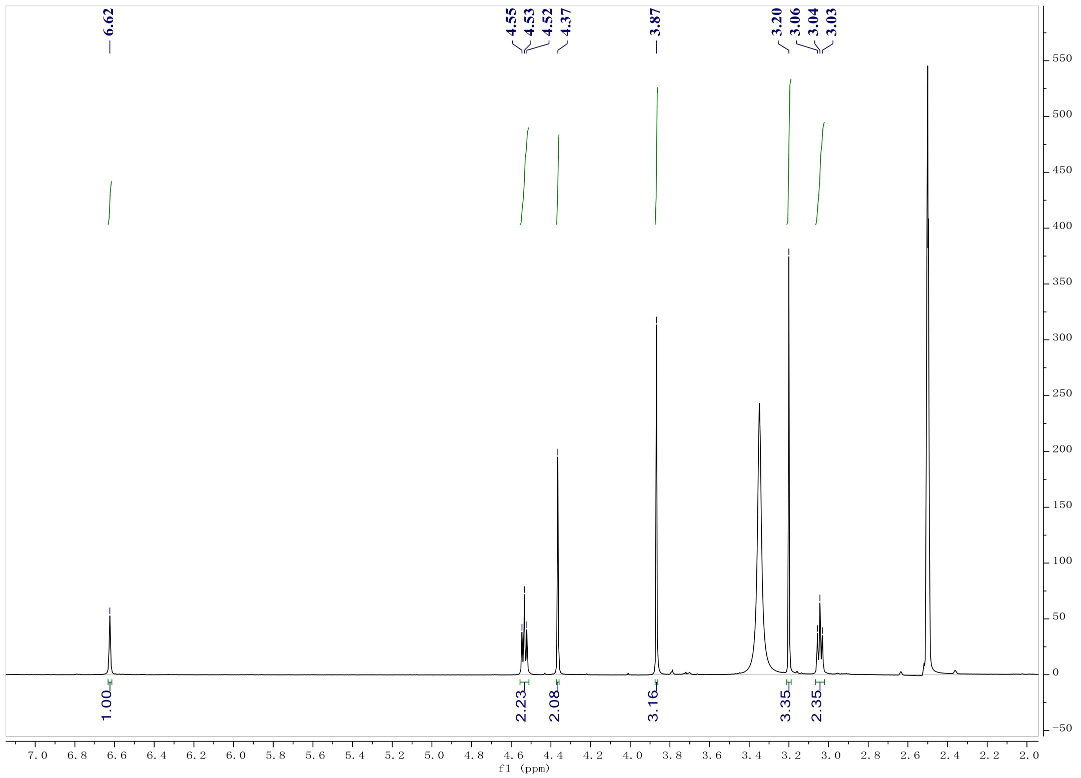

Figure S40. ^{1}H NMR spectrum (500 MHZ) of asperisocoumarin K (**6**) in DMSO-d_6.

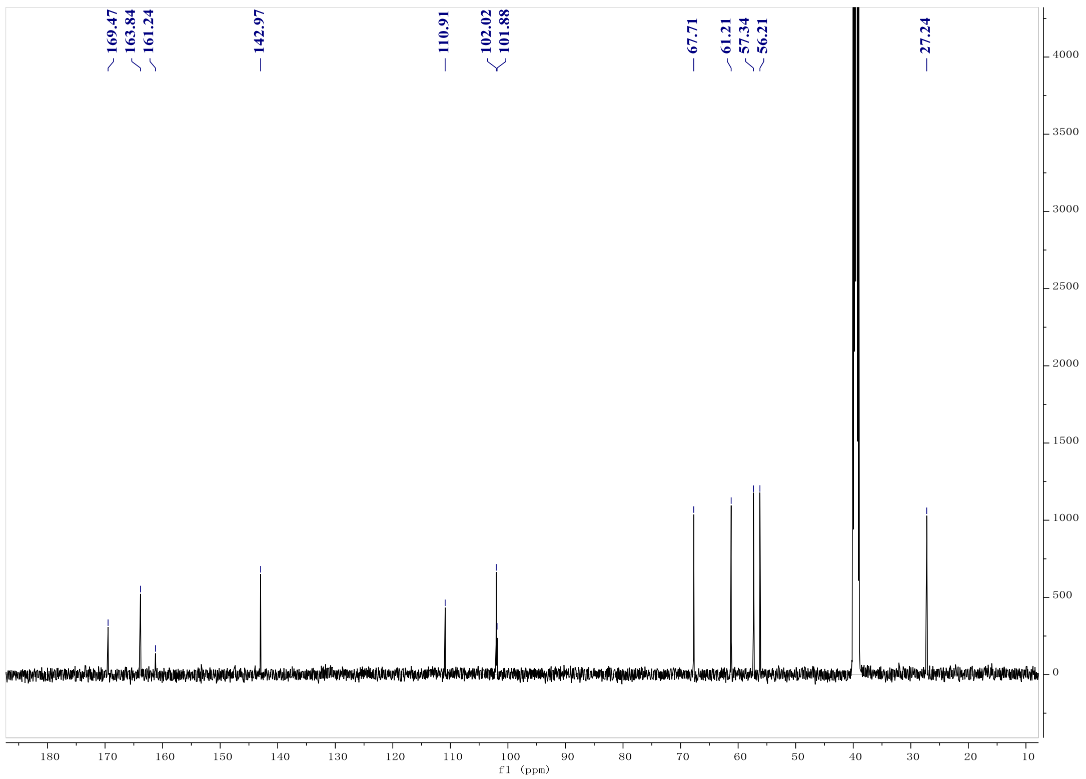

Figure S41. ^{13}C NMR spectrum (125 MHZ) of asperisocoumarin K (**6**) in DMSO-d_6.

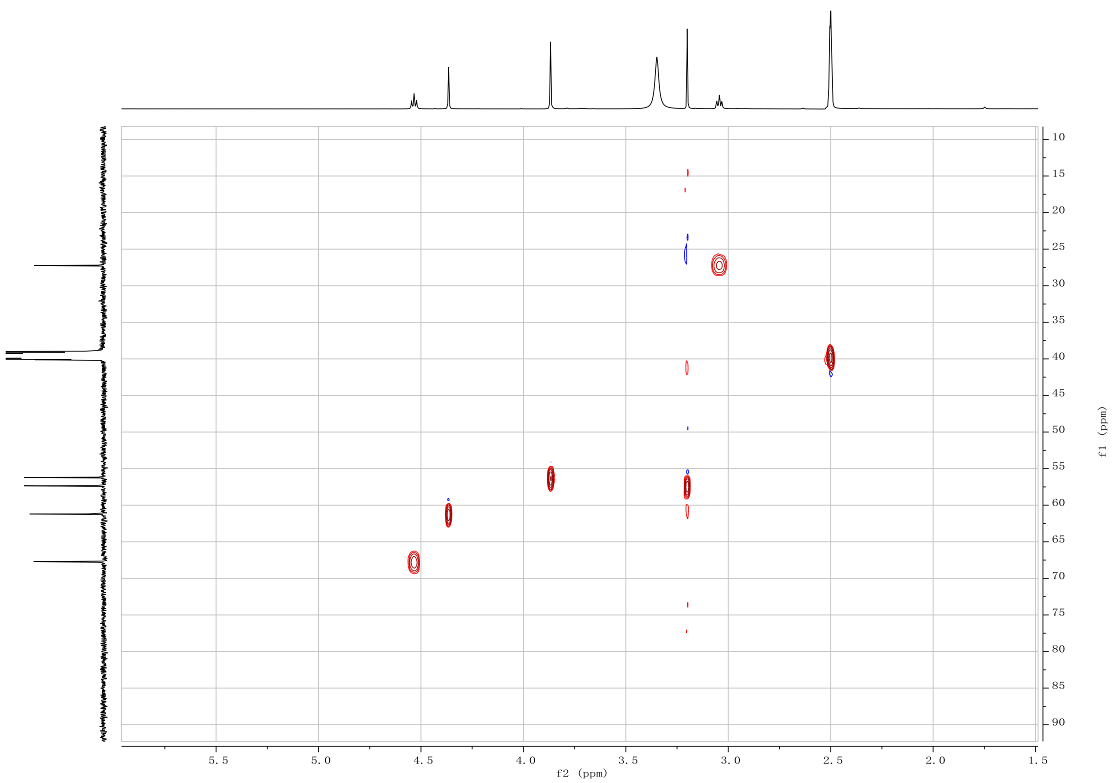

Figure S42. HSQC spectrum (500/125 MHZ) of asperisocoumarin K (**6**) in DMSO-*d*6.

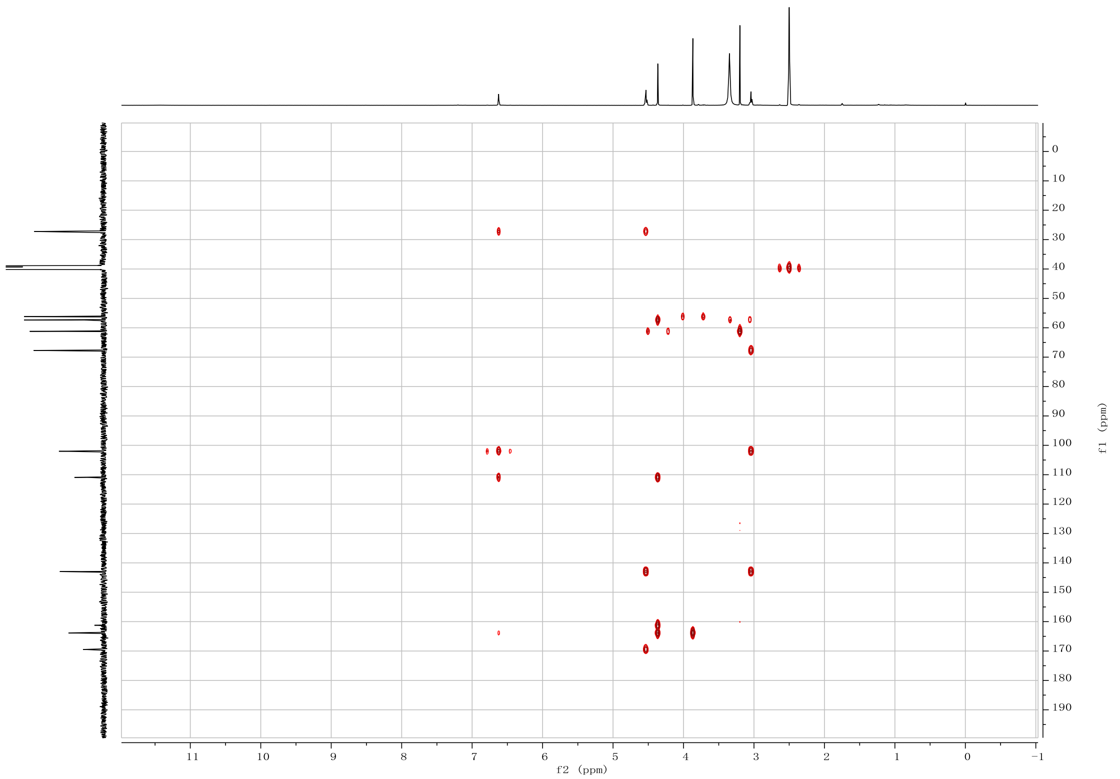

Figure S43. HMBC spectrum (500/125 MHZ) of asperisocoumarin K (**6**) in DMSO-*d*6.

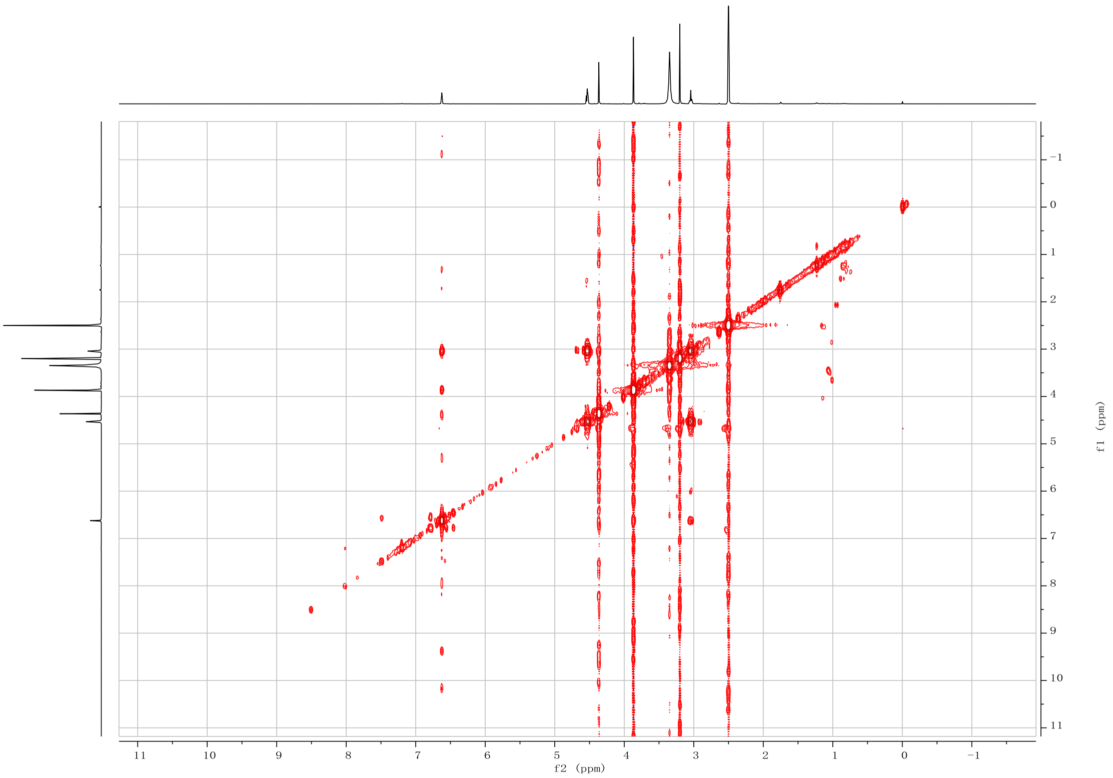

Figure S44. ^{1}H–^{1}H COSY spectrum (500 MHZ) of asperisocoumarin K (**6**) in DMSO-*d*$_6$.

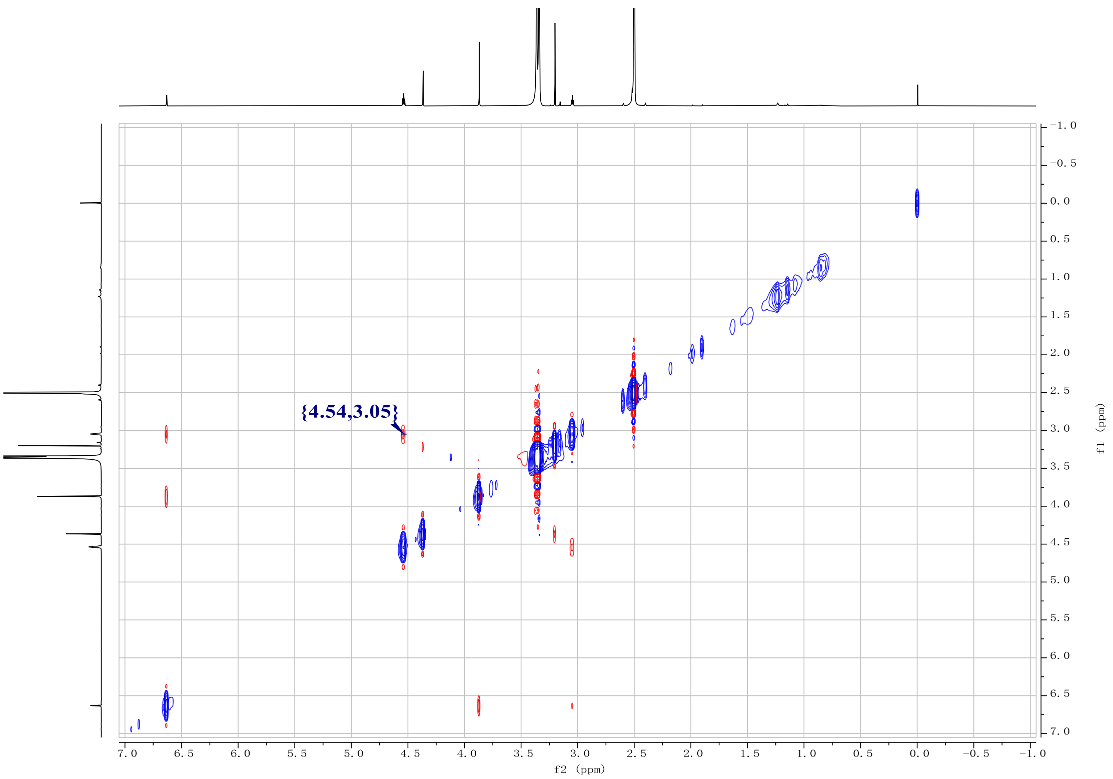

Figure S45. NOESY spectrum (700 MHZ) of asperisocoumarin K (**6**) in DMSO-*d*$_6$.

Generic Display Report

**Analysis Info**		Acquisition Date	7/4/2024 11:26:57 AM
Analysis Name	D:\Data\MS\data\202407\chenyi_4D28_pos_23_01_17609.d		
Method	LC_Direct Infusion_pos_70-500mz.m	Operator	SCSIO
Sample Name	chenyi_4D28_pos	Instrument	maXis
Comment			

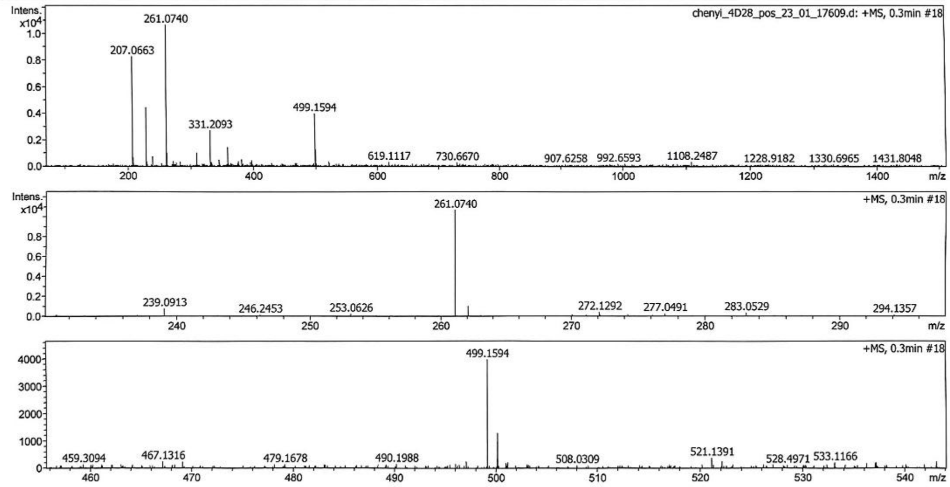

Bruker Compass DataAnalysis 4.1 printed: 7/4/2024 11:33:07 AM by: SCSIO Page 1 of 1

Mass Spectrum SmartFormula Report

**Analysis Info**		Acquisition Date	7/4/2024 11:26:57 AM	
Analysis Name	D:\Data\MS\data\202407\chenyi_4D28_pos_23_01_17609.d			
Method	LC_Direct Infusion_pos_70-500mz.m	Operator	SCSIO	
Sample Name	chenyi_4D28_pos	Instrument	maXis	255552.00029
Comment				

Acquisition Parameter

Source Type	ESI	Ion Polarity	Positive	Set Nebulizer	0.4 Bar
Focus	Active	Set Capillary	4500 V	Set Dry Heater	180 °C
Scan Begin	70 m/z	Set End Plate Offset	-500 V	Set Dry Gas	4.0 l/min
Scan End	1500 m/z	Set Charging Voltage	0 V	Set Divert Valve	Waste
		Set Corona	0 nA	Set APCI Heater	0 °C

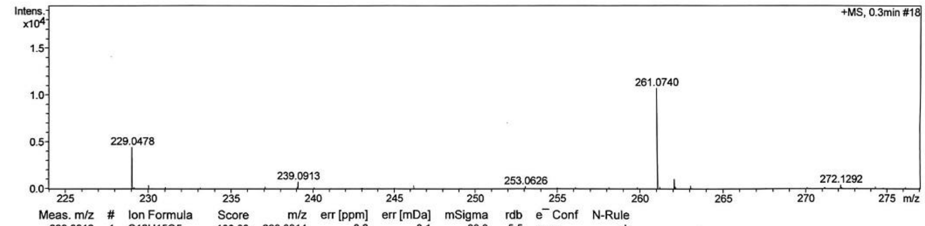

Meas. m/z	#	Ion Formula	Score	m/z	err [ppm]	err [mDa]	mSigma	rdb	e⁻ Conf	N-Rule
239.0913	1	C12H15O5	100.00	239.0914	0.2	0.1	39.9	5.5	even	ok
261.0740	1	C12H14NaO5	100.00	261.0733	2.6	0.7	21.8	5.5	even	ok
499.1594	1	C24H28NaO10	100.00	499.1575	-4.0	-2.0	28.4	10.5	even	ok

chenyi_4D28_pos_23_01_17609.d
Bruker Compass DataAnalysis 4.1 printed: 7/4/2024 11:32:55 AM by: SCSIO Page 1 of 1

Figure S46. HRESIMS spectra of asperisocoumarin K (**6**).

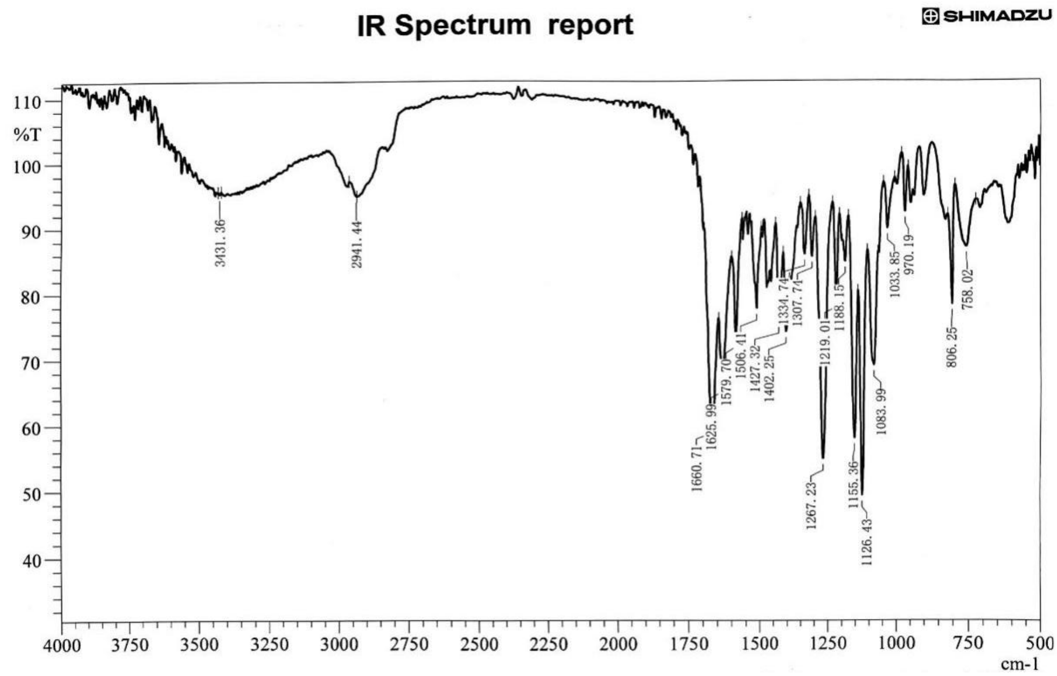

Figure S47. IR spectrum of asperisocoumarin K (6).

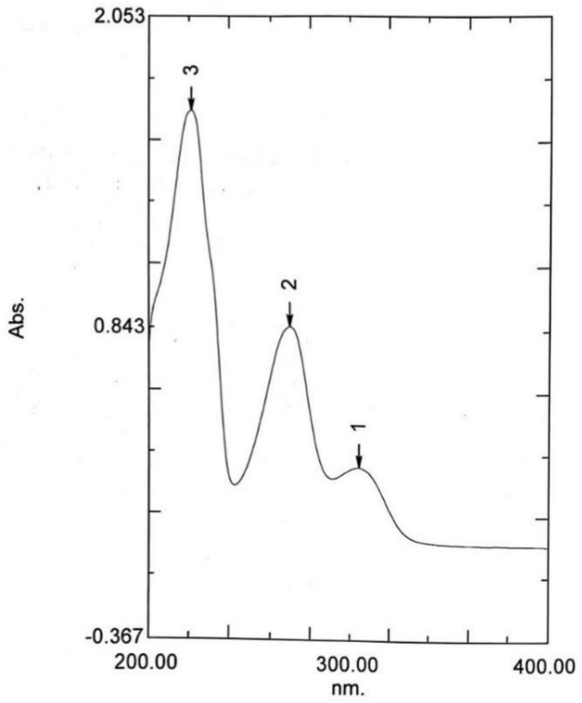

No.	P/V	波长(nm)	Abs.	描述
1		304.20	0.306	
2		270.00	0.852	
3		221.20	1.686	

Figure S48. UV spectrum of asperisocoumarin K (**6**) in CH_3OH.

4 ^{1}H and ^{13}C NMR spectra of compounds 7–12

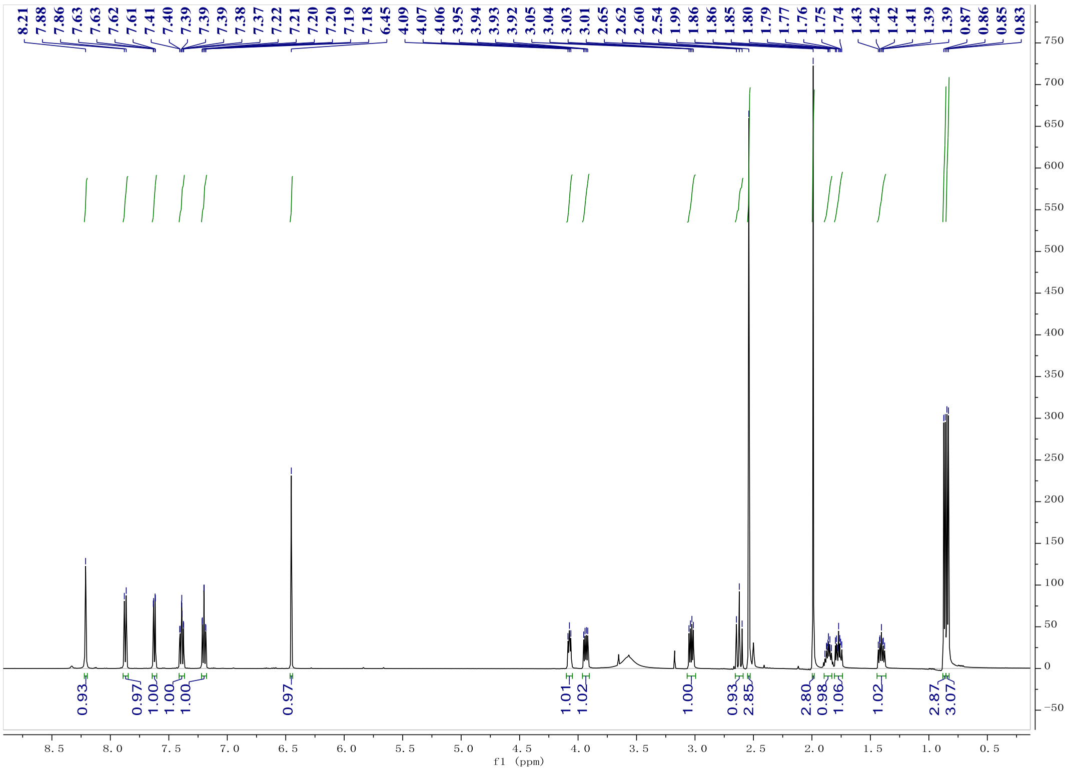

Figure S49. ^{1}H NMR spectrum (500 MHZ) of protubonine B (**7**) in DMSO-d_6.

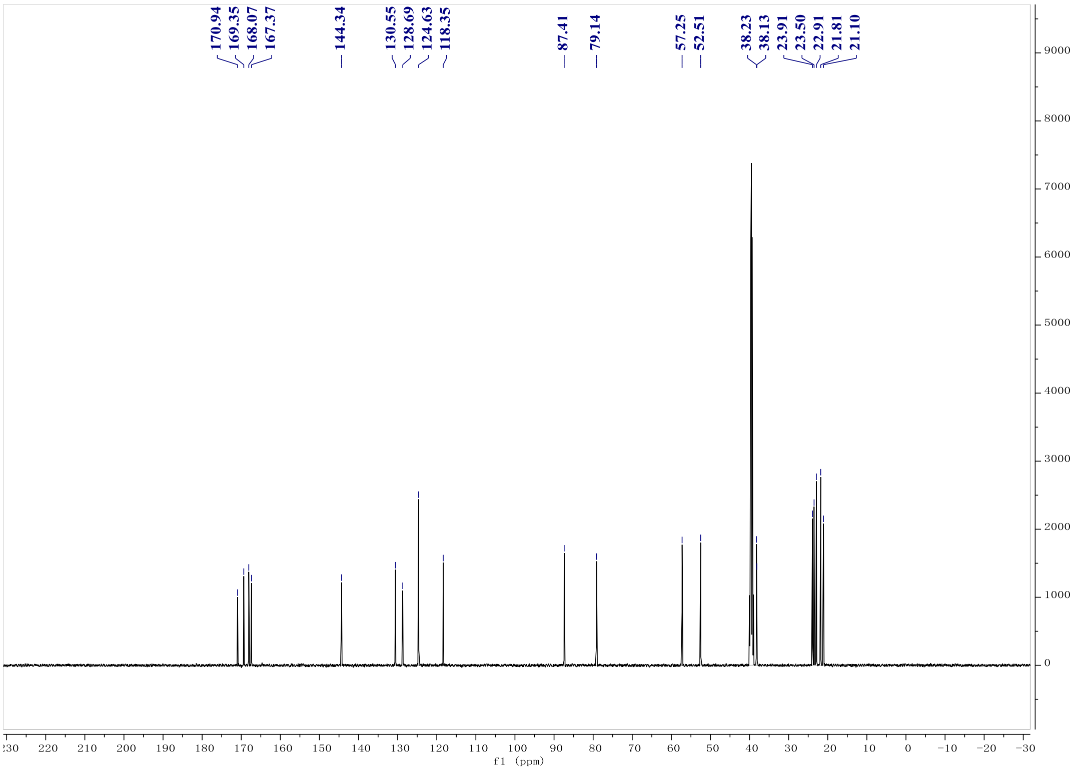

Figure S50. ^{13}C NMR spectrum (125 MHZ) of protubonine B (**7**) in DMSO-d_6.

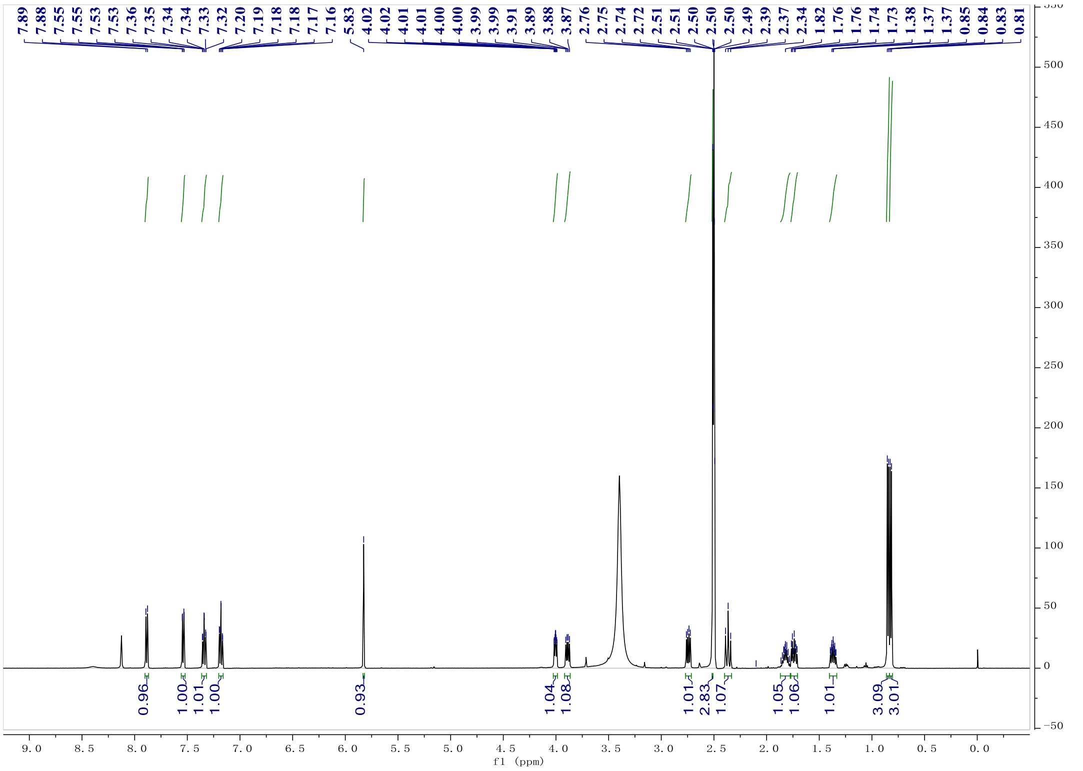

Figure S51. ^{1}H NMR spectrum (500 MHZ) of protubonine A (**8**) in DMSO-d_6.

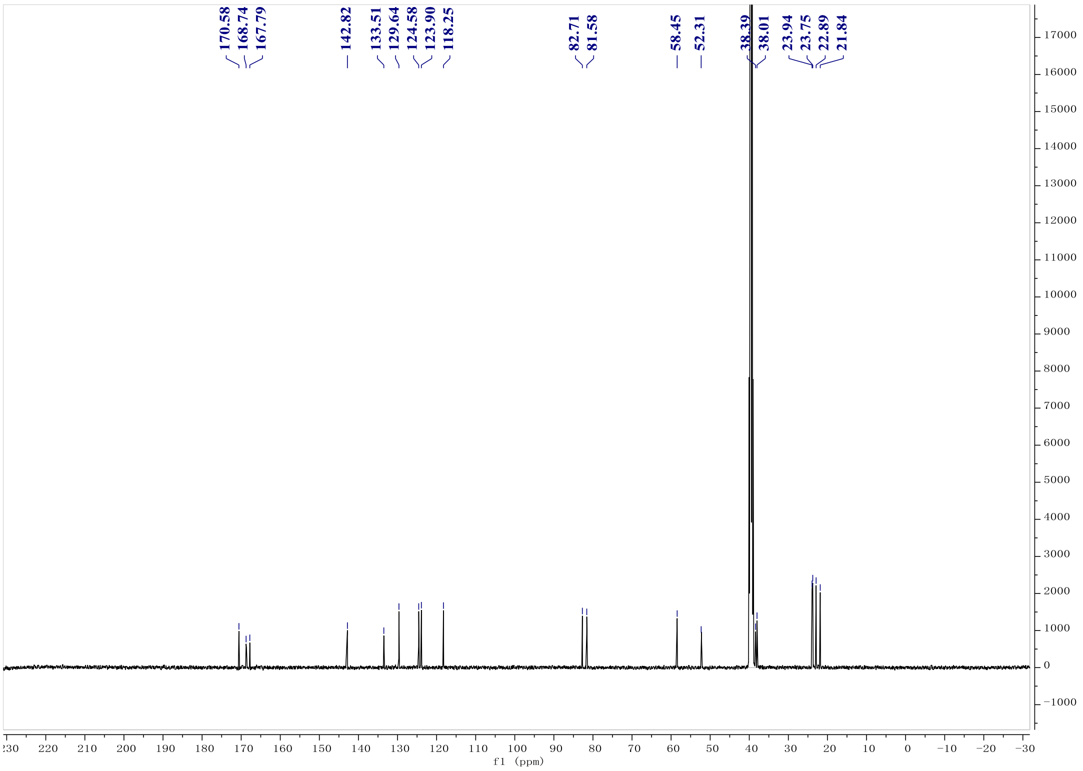

Figure S52. ^{13}C NMR spectrum (125 MHZ) of protubonine A (**8**) in DMSO-d_6.

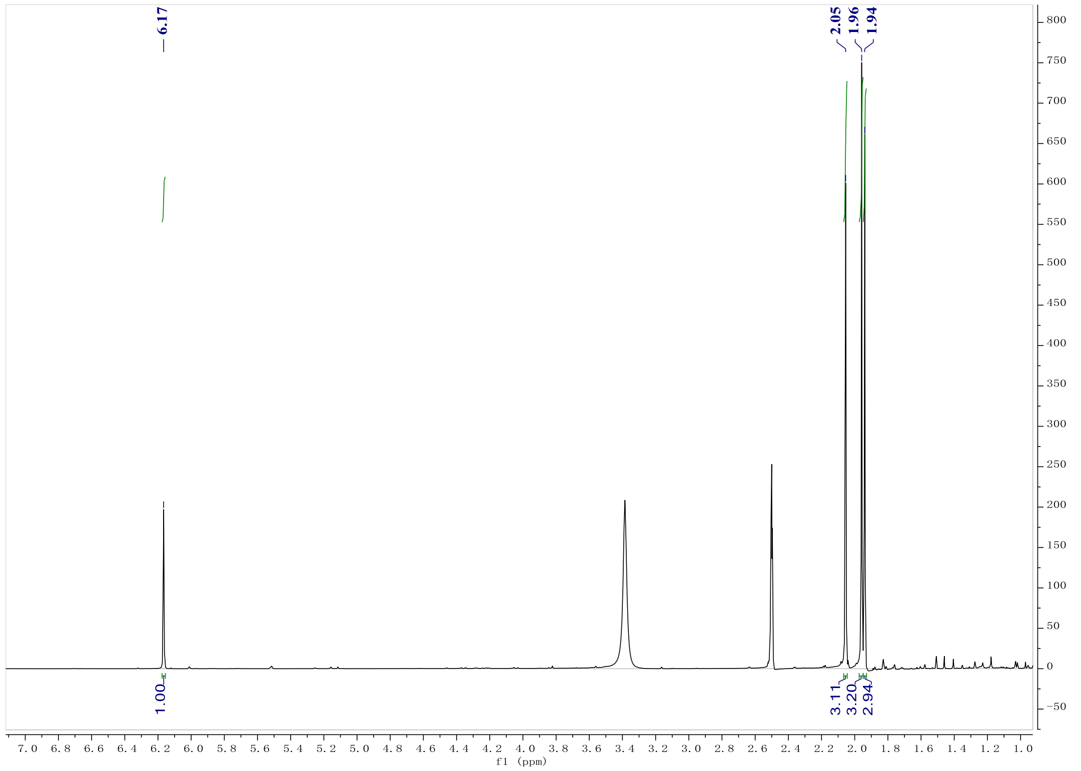

Figure S53. ^{1}H NMR spectrum (500 MHZ) of 2,4-dihydroxy-3,5,6-trimethylbenzene (**9**) in DMSO-d_6.

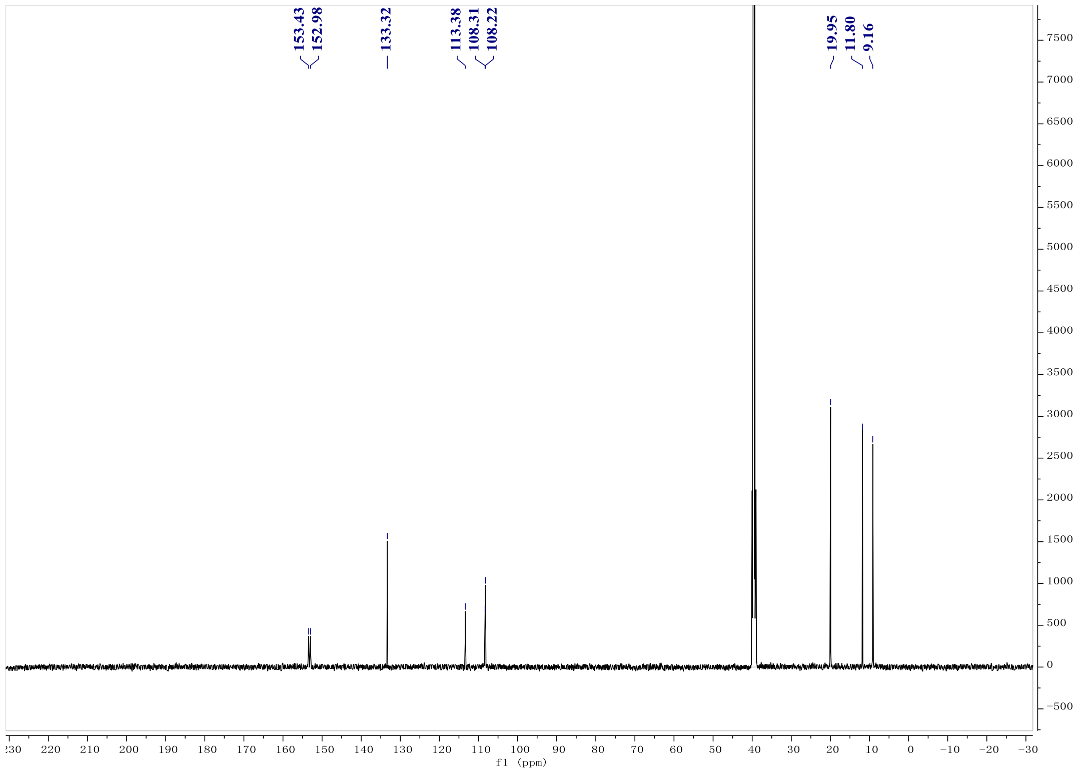

Figure S54. ^{13}C NMR spectrum (125 MHZ) of 2,4-dihydroxy-3,5,6-trimethylbenzene (**9**) in DMSO-d_6.

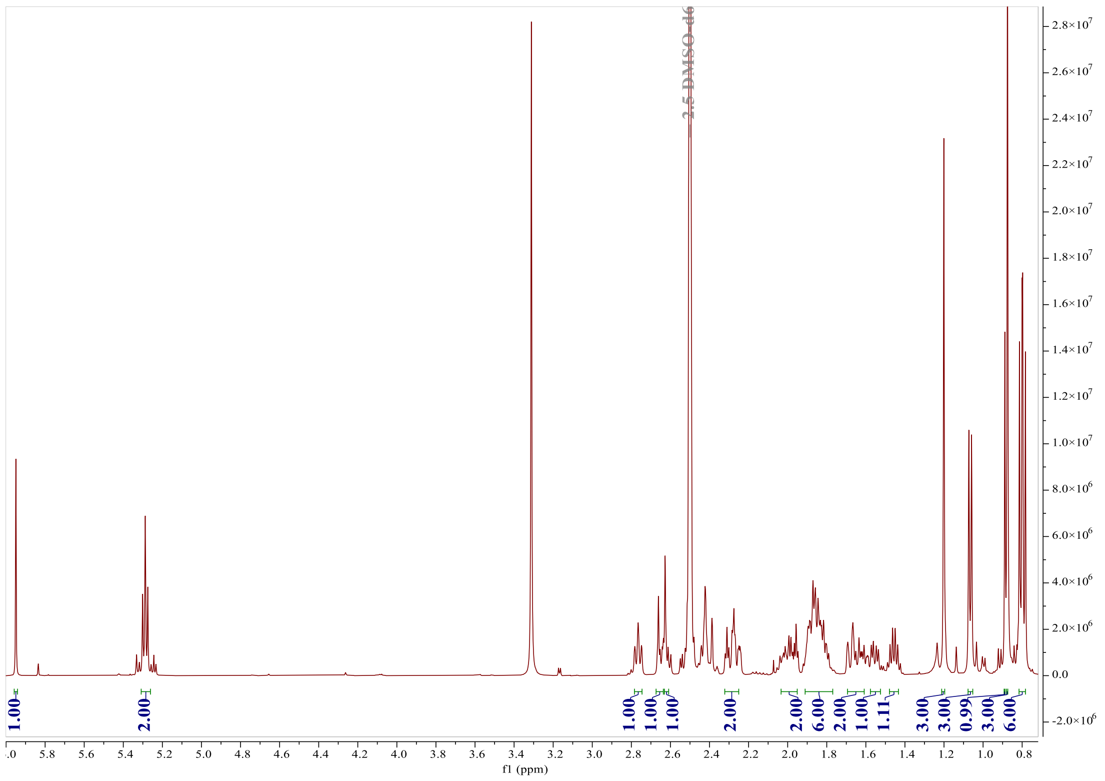

Figure S55. ^{1}H NMR spectrum (500 MHZ) of dankasterones A (**10**) in DMSO-*d*$_6$.

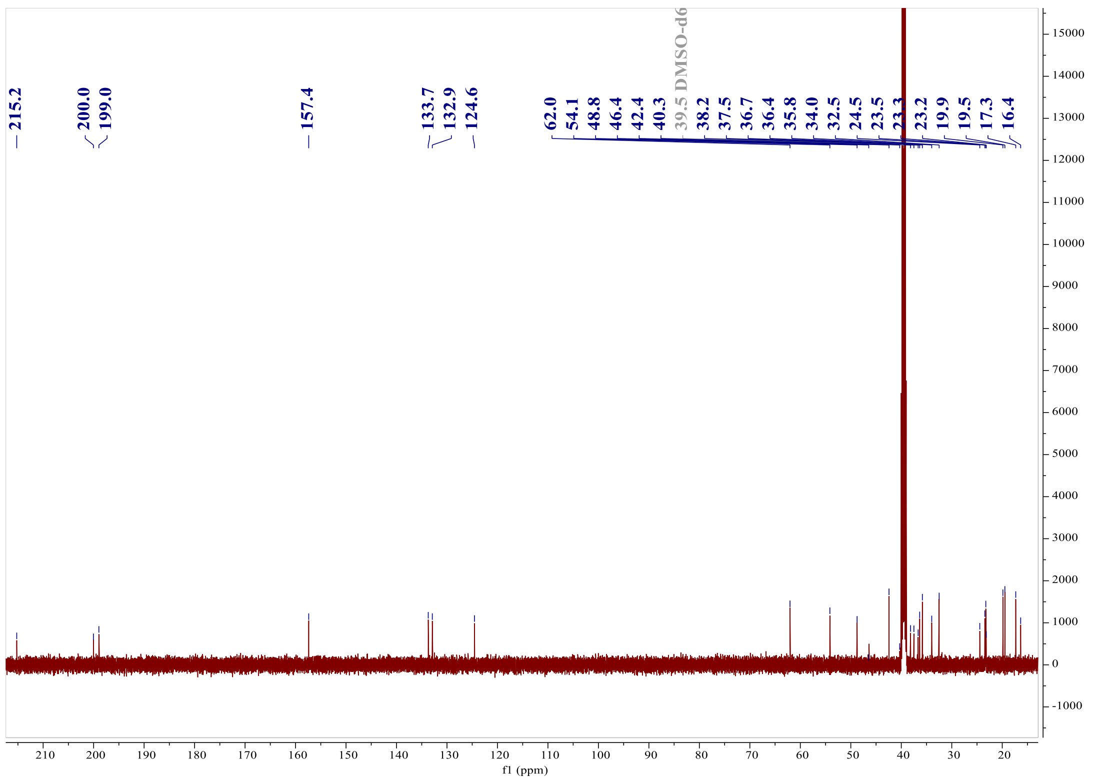

Figure S56. ^{13}C NMR spectrum (125 MHZ) of dankasterones A (**10**) in DMSO-*d*$_6$.

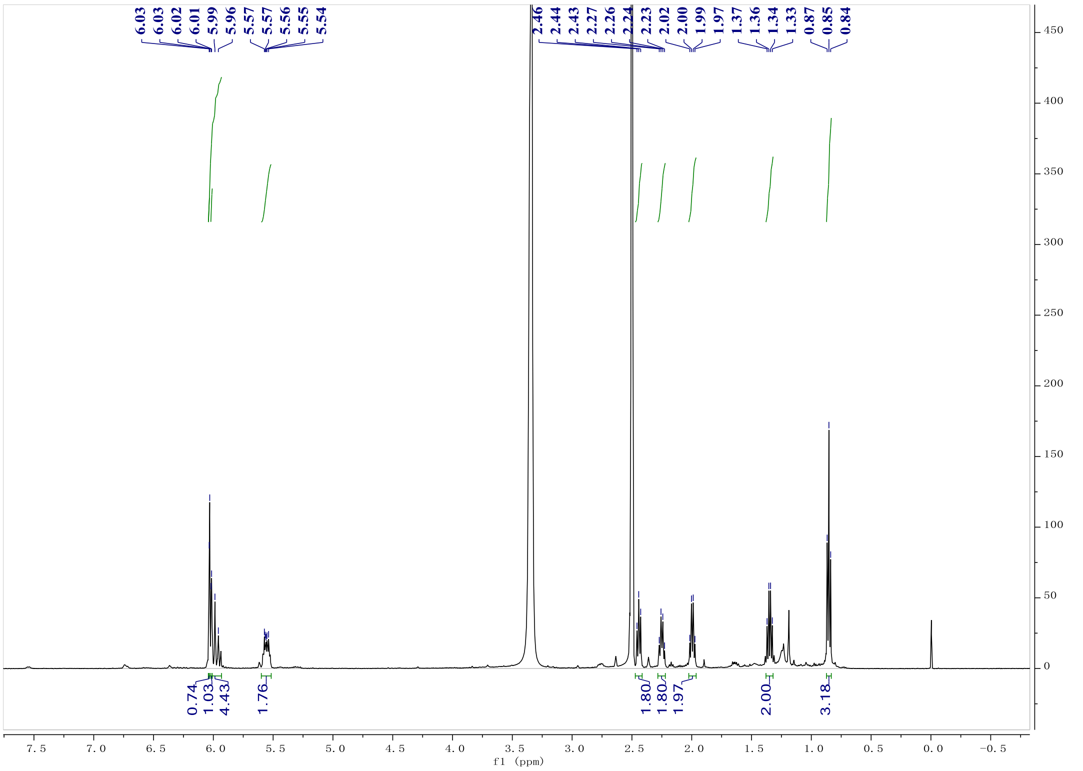

Figure S57. ^{1}H NMR spectrum (500 MHZ) of 5-(Undeca-3',5',7'-trien-1'-yl) furan-2-ol (**11**) in DMSO-d_6.

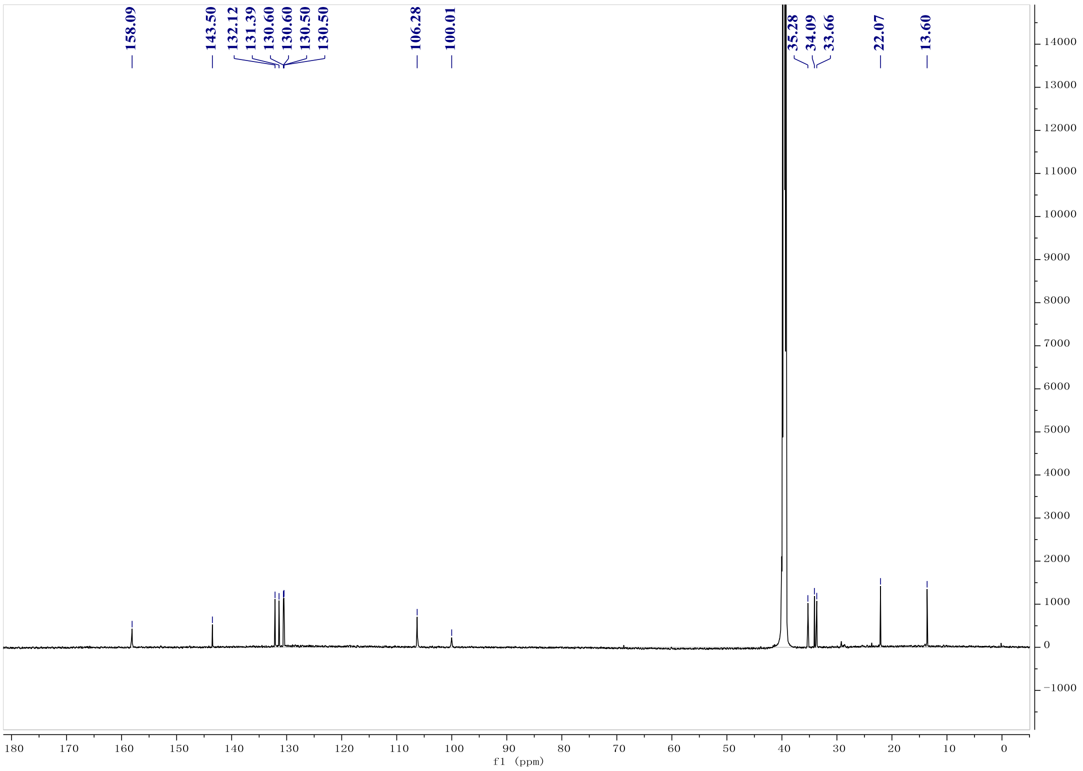

Figure S58. ^{13}C NMR spectrum (125 MHZ) of 5-(Undeca-3',5',7'-trien-1'-yl) furan-2-ol (**11**) in DMSO-d_6.

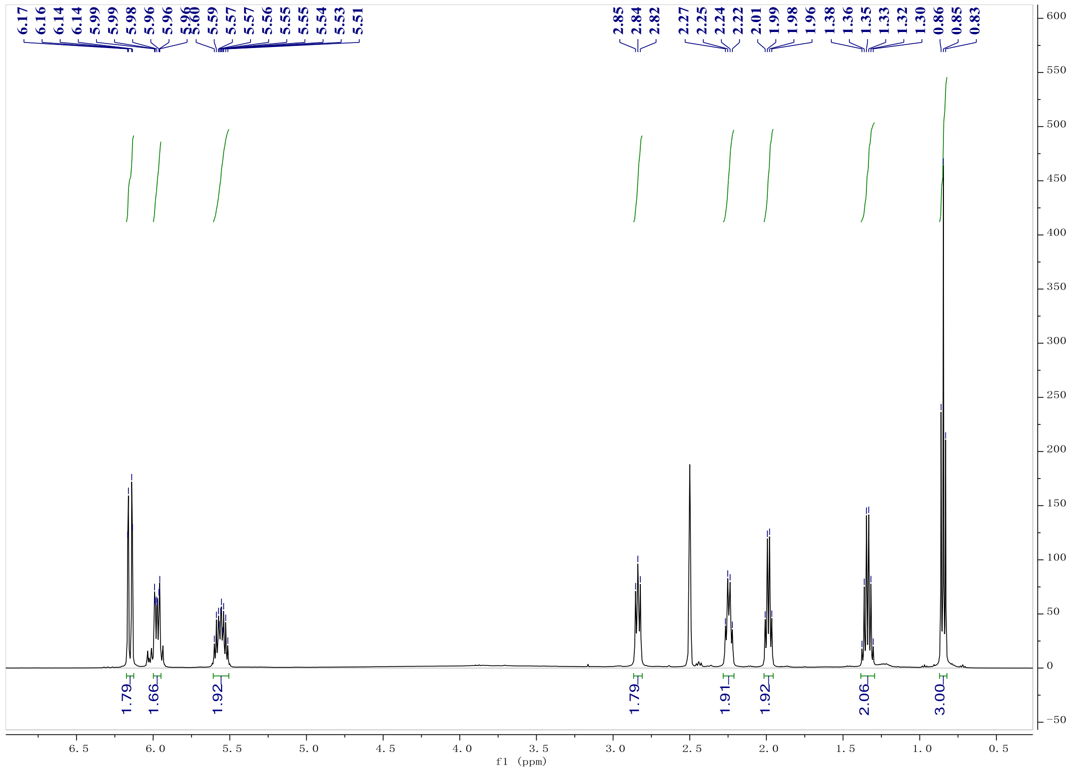

Figure S59. ^{1}H NMR spectrum (500 MHZ) of 2,4-dihydroxy-6-((3*E*,5*E*)-nona-3,5-dien-1-yl) benzoic acid (**12**) in DMSO-*d*6.

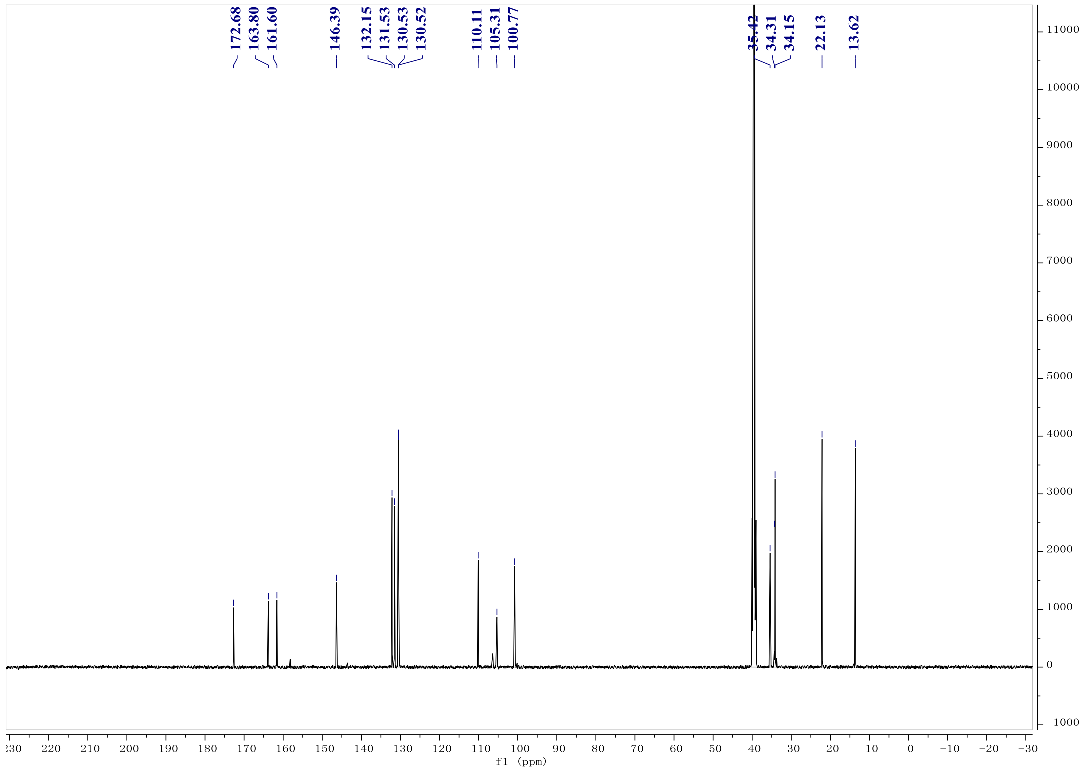

Figure S60. ^{13}C NMR spectrum (125 MHZ) of 2,4-dihydroxy-6-((3*E*,5*E*)-nona-3,5-dien-1-yl) benzoic acid (**12**) in DMSO-*d*6.

Reference for Supporting Information

1. Lee, S. U.; Asami, Y.; Lee, D.; Jang, J.-H.; Ahn, J. S.; Oh, H. Protuboxepins A and B and Protubonines A and B from the Marine-Derived Fungus *Aspergillus* sp. SF-5044. *J. Nat. Prod.* **2011,** *74* (5), 1284-1287. https://doi.org/10.1021/np100880b.
2. Wang, L.; Qin, D.; Zhang, K.; Huang, Q.; Liu, S.; Han, M. J.; Dong, J. Y. Metabolites from the co-culture of nigranoic acid and Umbelopsis dimorpha SWUKD3.1410, an endophytic fungus from *Kadsura angustifolia*. *Nat. Prod. Res.* **2017,** *31* (12), 1414-1421. https://doi.org/10.1080/14786419.2016.1255891.
3. Amagata, T.; Tanaka, M.; Yamada, T.; Doi, M.; Minoura, K.; Ohishi, H.; Yamori, T.; Numata, A. Variation in Cytostatic Constituents of a Sponge-Derived *Gymnascella dankaliensis* by Manipulating the Carbon Source. *J. Nat. Prod.* **2007,** *70* (11), 1731-1740. https://doi.org/10.1021/np070165m.
4. Wu, X.; Pang, X. J.; Xu, L. L.; Zhao, T.; Long, X. Y.; Zhang, Q. Y.; Qin, H. L.; Yang, D. F.; Yang, X. L. Two new alkylated furan derivatives with antifungal and antibacterial activities from the plant endophytic fungus *Emericella* sp. XL029. *Nat. Prod. Res.* **2018,** *32* (22), 2625-2631. https://doi.org/10.1080/14786419.2017.1374269.
5. Sun, W. L.; Zhuang, C. L.; Li, X.; Zhang, B. W.; Lu, X. H.; Zheng, Z. H.; Dong, Y. S. Varic acid analogues from fungus as PTP1B inhibitors: Biological evaluation and structure–activity relationships. *Bioorg. Med. Chem. Lett.* **2017,** *27* (15), 3382-3385. https://doi.org/10.1016/j.bmcl.2017.06.001.
6. Bringmann, G.; Lang, G.; Steffens, S.; Günther, E.; Schaumann, K. Evariquinone, isoemericellin, and stromemycin from a sponge derived strain of the fungus *Emericella variecolor*. *Phytochemistry* **2003,** *63* (4), 437-443. https://doi.org/10.1016/S0031-9422(03)00189-4.
